# Integrated analysis of 34 microarray datasets reveals *CBX3* as a diagnostic and prognostic biomarker in glioblastoma

**DOI:** 10.1186/s12967-019-1930-3

**Published:** 2019-05-28

**Authors:** Siqi Wang, Fang Liu, Yuhui Wang, Wenliang Fan, Hongyang Zhao, Liying Liu, Chunyuan Cen, Xiaobin Jiang, Min Sun, Ping Han

**Affiliations:** 1 Department of Radiology, Union Hospital, Tongji Medical College, Huazhong University of Science and Technology, Wuhan, 430022 China; 2 Hubei Province Key Laboratory of Molecular Imaging, Wuhan, 430022 China; 3 Department of General Surgery, Taihe Hospital, Hubei University of Medicine, Shiyan, 442000 China; 4 Department of Neurosurgery, Union Hospital, Tongji Medical College, Huazhong University of Science and Technology, Wuhan, 430022 China; 5 Department of Nuclear Medicine, Union Hospital, Tongji Medical College, Huazhong University of Science and Technology, Wuhan, 430022 China

**Keywords:** Glioblastoma, Biomarker, Diagnosis, Prognosis, Differentially expressed gene

## Abstract

**Background:**

Glioblastomas have a high degree of malignancy, high recurrence rate, high mortality rate, and low cure rate. Searching for new markers of glioblastomas is of great significance for improving the diagnosis, prognosis and treatment of glioma.

**Methods:**

Using the GEO public database, we combined 34 glioma microarray datasets containing 1893 glioma samples and conducted genetic data mining through statistical analysis, bioclustering, and pathway analysis. The results were validated in TCGA, CGGA, and internal cohorts. We further selected a gene for subsequent experiments and conducted cell proliferation and cell cycle analyses to verify the biological function of this gene.

**Results:**

Eight glioblastoma-specific differentially expressed genes were screened using GEO. In the TCGA and CGGA cohorts, patients with high *CBX3*, *BARD1*, *EGFR*, or *IFRD1* expression had significantly shorter survival but patients with high *GUCY1A3* or *MOBP* expression had significantly longer survival than patients with lower expression of these genes. After reviewing the literature, we selected the *CBX3* gene for further experiments. We confirmed that *CBX3* was overexpressed in glioblastoma by immunohistochemical analysis of tissue microarrays and qPCR analysis of surgical specimens. The functional assay results showed that silencing *CBX3* arrests the cell cycle in the G2/M phase, thereby weakening the cell proliferation ability.

**Conclusions:**

We used a multidisciplinary approach to analyze glioblastoma samples in 34 microarray datasets, revealing novel diagnostic and prognostic biomarkers in patients with glioblastoma and providing a new direction for screening tumor markers.

**Electronic supplementary material:**

The online version of this article (10.1186/s12967-019-1930-3) contains supplementary material, which is available to authorized users.

## Background

Gliomas are the most common primary tumors in the central nervous system. According to the WHO criteria published in 2007 [1] and 2016 [2], gliomas are graded from I to IV, mainly including grade I–IV astrocytomas and grade II–IV oligodendrogliomas. Grade IV astrocytomas are known as glioblastoma (GBM), which is the most malignant and lethal glioma. GBM is characterized by high proliferation, infiltrative growth behavior, intratumoral heterogeneity and tumor recurrence. Despite improvements in GBM therapy that involve surgical resection, radiation and chemotherapy, a cure for GBM appears elusive. Additionally, the median survival is only 12–15 months for patients with glioblastomas [3]. The emergence of genomic and proteomic profiling has provided more insight into the oncogenesis, characterization, and therapy of gliomas.

The integration of molecular biomarkers with histological assessment has yielded new insights into gliomas [4–6]. Some molecular biological markers are important for determining molecular subtypes, individualized treatment, and predicting prognosis, such as MGMT [7], EGFR [8], IDH [9], 1p19q [10], ATRX [11], MGMT promoter methylation levels, 1p/19q-codeleted and IDH1 mutations can predict the prognosis of GBM, oligodendroglioma (OD) and low grade glioma [12]. IDH1 or IDH2 mutations can exist in glioblastomas, especially evolved from lower-grade gliomas, and patients with such tumors had a better outcome than those with wild-type IDH genes [13, 14]. G-CIMP-positive status appears in most WHO grade II and III gliomas and secondary glioblastomas and is correlated with improved patient survival [15]. Other studies used EGFR, NF1, and PDGFRA/IDH1 to classify GBM into pro-neural, neural, classical, and mesenchymal subtypes [16, 17]. Although emerging evidence supports mRNAs as potential biomarkers of glioblastomas, gene expression studies analyzed in isolation usually have inconsistent or discrepant results. Various factors, such as limited sample sizes, different profiling platforms, and diverse methods for data collection and analysis, lead to these discrepancies. Furthermore, approximately half of all patients do not harbor known “driver” genes and cannot be treated with targeted agents. The NCBI Gene Expression Omnibus (GEO) contains numerous human microarray datasets from various types of tissue biopsies, which can be used to discover disease-associated biomarkers. These datasets represent a large and incompletely exploited resource for discovering novel biomarkers. However, the existence of biological (cohort selection) and technical (treatment protocol and microarray technology) differences in individual studies hindered the broader application of these findings and ultimately limited their translation into clinical practice. Thus, new approaches for the identification of novel biomarkers of gliomas are needed.

To overcome these limitations, we need an integrated and unbiased method of analyzing results and obtaining mRNAs with greater statistical significance. Through integrated analysis approaches, such confounding factors can be controlled by increasing the statistical power, thus allowing the detection of consistent biomarkers across multiple studies; such methods have been applied in analyses in breast cancer [18], prostate cancer [19], diffuse low-grade glioma [20], and lung cancer [21].

Among the gene expression signatures identified in our study, chromobox homolog 3 (CBX3/heterochromatin protein 1γ [HP1γ]), a member of the heterochromatin protein 1 (HP1) family, has the ability to regulate the structure of both heterochromatin and euchromatin, suggesting that it may participate in both transcriptional repression and activation [22]. CBX3 is associated with the epigenetic regulation of cell differentiation and cancer development [23]. Recently, *CBX3* was revealed to be associated with lung cancer [24], osteosarcoma [25], gastric cardia adenocarcinoma [26] and colorectal cancer [27]. However, the precise role of *CBX3* in glioma remains unclear, although Holmberg et al. [28] reported that HP1γ is associated with *NPM1*, which functions in the spatial organization of nucleolus-associated heterochromatin in glioma.

We performed integrated analyses of 34 gene expression datasets consisting of 1893 glioma samples, which enabled the discovery and validation of eight differentially expressed genes (DEGs) consistently and specifically expressed in GBM. We further validated aberrant expression patterns of eight DEGs in datasets from The Cancer Genome Atlas (TCGA) and the Chinese Glioma Genome Atlas (CGGA) and revealed that six DEGs are prognostic biomarkers of glioma. CBX3 was distinguished as a novel and clinically noteworthy mRNA in GBM. Further experiments showed that *CBX3* was overexpressed in glioblastoma tissues and is a potential diagnostic and prognostic biomarker. Silencing *CBX3* can arrest the cell cycle in the G2/M phase in U373 cells, thereby weakening the cell proliferation ability. We attempt to provide novel and critical biomarkers that might be beneficial for the precise diagnosis and prognostic prediction of GBM and have broader application for translation into clinical practice.

## Materials and methods

### Data sources

**Fig. 1:**
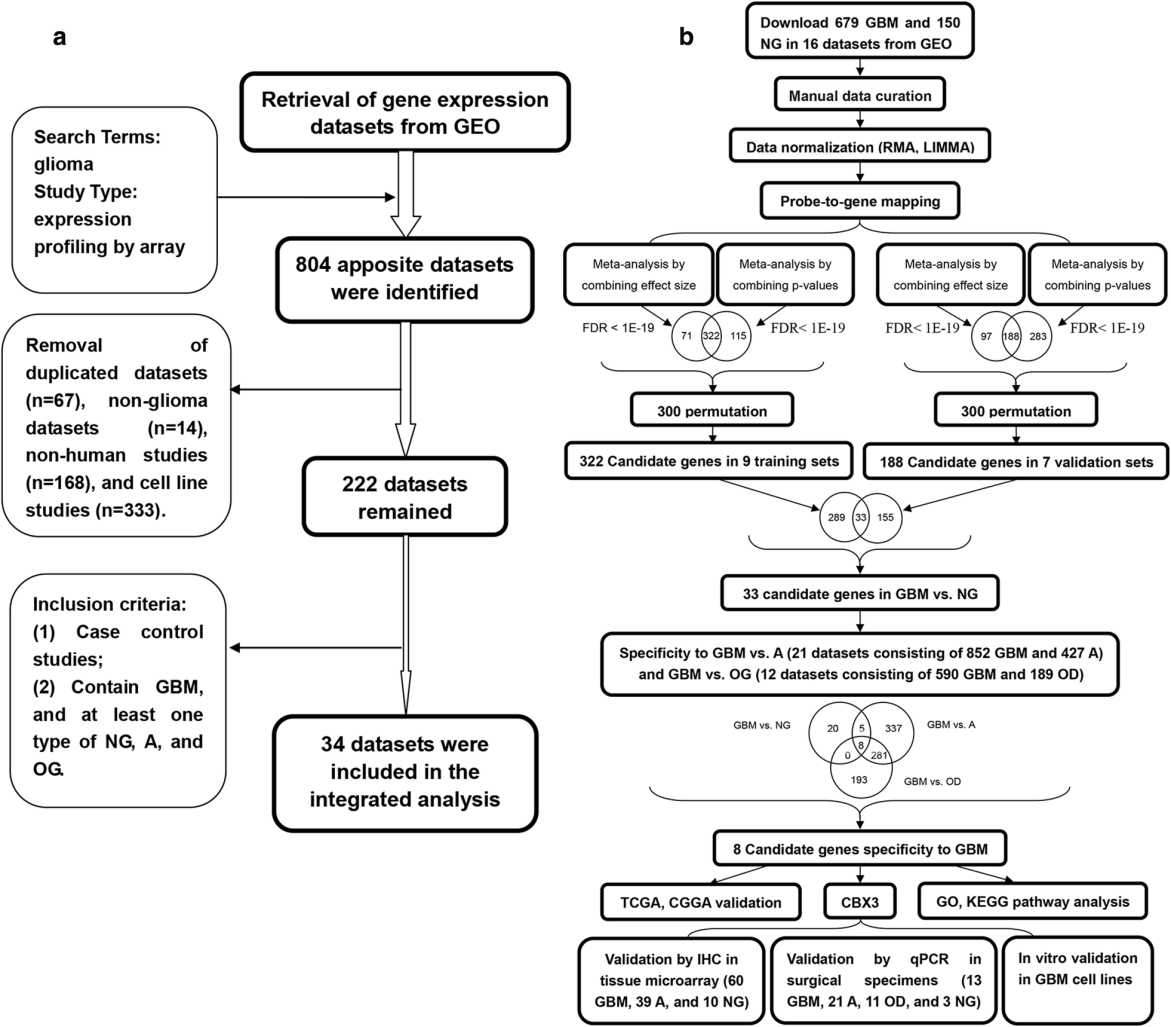
Study workflow. **a** Workflow of the process for identifying microarray datasets for integrated analysis. **b** Overall steps in the integrated microarray analysis. GBM: glioblastoma; A: astrocytoma; OD: oligodendroglioma; NG: nonglioma; GEO: Gene Expression Omnibus; TCGA: The Cancer Genome Atlas; CGGA: the Chinese Glioma Genome Atlas; GO: Gene Ontology; KEGG: Kyoto Encyclopedia of Genes and Genomes

We searched the NCBI database (http://www.ncbi.nlm.nih.gov/geo/) for glioma gene expression profiling studies published through December 2016. The inclusion criteria were as follows: human case/control studies, studies with untreated samples, studies with available raw or processed data, studies including GBM, and studies including at least one type of nonglioma (NG), astrocytoma (A), and OD sample. Figure 1a shows the workflow for identifying eligible datasets. Gene expression data for 31 human glioma studies were downloaded (GSE4058, GSE2223, GSE4290, GSE4271, GSE4412, GSE9885, GSE1993, GSE12657, GSE13276, GSE19728, GSE16011, GSE24072, GSE30563, GSE15824, GSE22866, GSE38330, GSE50161, GSE43289, GSE45921, GSE52009, GSE43911, GSE54004, GSE62802, GSE68848, GSE68015, GSE66354, GSE70231, GSE68928, GSE74462, GSE43378, and GSE82009; see Table 1). The histological subtypes were defined according to the original publications. The datasets were curated to include only GBM, A, OD and NG. Oligoastrocytoma (OA) was excluded because it is not recognized as a separate tumor entity in the 2016 CNS tumor classification system [2]. All datasets were normalized individually using robust multiarray averaging [29]. The microarray probes in each dataset were mapped to gene symbols to facilitate meta-analysis.

**Table 1:** Characteristics of datasets included in the integrated analysis

Year	Source accession	First author	Country	Assay type	Platform	PMID
2006	GSE4058	Diehn M	USA	SHV	GPL182	15827123
2006	GSE2223	Bredel M	USA	SHFK	GPL1833	16204036
2006	GSE4290	Fine HA	USA	Affymetrix Human Genome U133 Plus 2.0 Array	GPL570	16616334
2006	GSE4271	Phillips HS	USA	Affymetrix Human Genome U133A Array; Affymetrix Human Genome U133B Array	GPL96; GPL97	16530701
2006	GSE4412	Nelson SF	USA	Affymetrix Human Genome U133A Array; Affymetrix Human Genome U133B Array	GPL96; GPL97	15374961
2007	GSE9885	Marucci G	Italy	Agilent-011521 Human 1A Microarray G4110A; Agilent-012097 Human 1A Microarray (V2) G4110B	GPL885; GPL887	18953566
2007	GSE1993	Petalidis L	United Kingdom	Affymetrix Human Genome U133A Array	GPL96	18445660
2008	GSE12657	Moran LB	United Kingdom	Affymetrix Human Genome U95 Version 2 Array	GPL8300	NA
2009	GSE13276	Saulnier N	Italy	Affymetrix Human Genome U133A Array	GPL96	23472076
2010	GSE19728	Liu Z	China	Affymetrix Human Genome U133 Plus 2.0 Array	GPL570	21836821
2010	GSE16011	Gravendeel LA	Netherlands	Affymetrix GeneChip Human Genome U133 Plus 2.0 Arra	GPL8542	19920198
2010	GSE24072	Garcia JL	Spain	Affymetrix Human Genome U133A Array	GPL96	NA
2011	GSE30563	Lee M	South Korea	Affymetrix Human Genome U133 Plus 2.0 Array	GPL570	NA
2011	GSE15824	Morin PJ	Switzerland	Affymetrix Human Genome U133 Plus 2.0 Array	GPL570	21406405
2011	GSE22866	Etcheverry A	France	Agilent-014850 Whole Human Genome Microarray 4x44K G4112F	GPL4133	21156036
2012	GSE38330	Engler JR	USA	Agilent-014850 Whole Human Genome Microarray 4x44K G4112F; Agilent-020087 human whole genome 4x44K	GPL4133	22937035
2013	GSE50161	Donson AM	USA	Affymetrix Human Genome U133 Plus 2.0 Array	GPL570	24078694
2013	GSE43289	Tabernero MD	Spain	Affymetrix Human Genome U133 Plus 2.0 Array	GPL570	20484145
2013	GSE45921	Lu Y	China	Affymetrix Human Genome U133 Plus 2.0 Array	GPL570	23869222
2013	GSE52009	Jiang T	China	Agilent-014850 Whole Human Genome Microarray 4x44K	GPL6480	NA
2014	GSE43911	Hackermüller J	Germany	Agilent-021412 nONCOchip_1.0 021253	GPL13648	24594072
2014	GSE54004	deGroot J	USA	Illumina HumanHT-12 WG-DASL V4.0 R2 expression beadchip	GPL18281	NA
2015	GSE62802	Marc Remke	Canada	Affymetrix Human Genome U133 Plus 2.0 Array	GPL570	NA
2015	GSE68848	Fine H	USA	Affymetrix Human Genome U133 Plus 2.0 Array	GPL570	19208739
2015	GSE68015	Donson AM	USA	Affymetrix Human Genome U133 Plus 2.0 Array	GPL570	25990246
2015	GSE66354	Donson AM	USA	Affymetrix Human Genome U133 Plus 2.0 Array	GPL570	25968456
2015	GSE70231	Mervi Heiskanen	USA	Affymetrix Human Full Length HuGeneFL Array	GPL80	11559565
2015	GSE68928	Mervi Heiskanen	USA	Affymetrix Human Full Length HuGeneFL Array; Affymetrix Human 35 K SubA Array	GPL80; GPL98	11742071
2015	GSE74462	Fan X	China	Affymetrix Human Gene 1.0 ST Array [transcript (gene) version]	GPL6244	NA
2016	GSE43378	Ryuya yamanaka	Japan	Affymetrix Human Genome U133 Plus 2.0 Array	GPL570	23745793
2016	GSE82009	Mervi Heiskanen	USA	Affymetrix Human Genome U95 Version 2 Array	GPL8300	12670911

GSE: gene expression omnibus; GPL: gene platform

The transcriptome and methylation expression profiles, corresponding clinical parameters, and follow-up information for the patients with glioma were also downloaded from TCGA (https://tcga-data.nci.Nih.gov/tcga/) and the CGGA (http://www.cgcg.org.cn/) [30]. From the TCGA-GBMLGG dataset, we collected RNAseq data from 674 glioma samples, including 158 GBM, 193 A, 188 OD, 130 OA, and 5 NG samples. In the CGGA, transcriptome data for 225 samples, including 89 GBM, 66 A, 28 OD, 37 OA, and 5 NG samples, were available.

### Integrated analysis procedures

For the integrated analysis, the microarray datasets were subjected to quality control using the MetaQC package of R software (version 3.4.0). The mean and standard deviation filter thresholds were set at 10%. The datasets were analyzed using two different meta-analyses with the MetaDE package of R software (http://www.pitt.edu/~tsengweb/MetaOmicsHome.htm) [31]: (1) combining p-values and (2) combining effect sizes.

Four different meta-analysis methods in the package were used for combining p-values: fisher, maxP, roP, and AW. Using detection competency curves, the numbers of detected DEGs from four methods were compared to find the optimal methods. Fisher’s sum of logs method was performed in the meta-analysis, and the modified t test and permutation method were used [32]. Briefly, for each gene, we summed the logarithms of the p-values for one-sided hypothesis testing across all datasets. The study-specific effect sizes were combined to obtain the pooled effect size and the associated standard error using the random effects inverse variance model. After computing the meta-effect size, significant genes were identified using the z-statistic, and p-values were corrected for multiple hypothesis testing using the Benjamini–Hochberg false discovery rate (FDR) correction [33]. Considering the heterogeneity of gene expression across all samples and datasets, we used a very stringent threshold (FDR < 1 × 10^−19^) for both integrated analysis methods to identify DEGs in GBM vs. NG tissues. Figure 1b shows the overall steps in the integrated microarray analysis and functional validation pipeline.

### Functional analysis

To interpret the biological functions of the DEGs, Gene Ontology (GO) and Kyoto Encyclopedia of Genes and Genomes (KEGG) pathway analyses were performed using Enrichr tool [34]. The GO analyses covered three domains: biological process (BP), cellular component (CC) and molecular function (MF). Finally, the significant GO terms and KEGG pathways were filtered at a threshold of FDR < 0.05.

### Survival analysis

Log-rank tests for significance were conducted and Kaplan–Meier curves were plotted using GraphPad Prism 5. According to the median expression level of each DEG, patients with glioma were divided into low and high DEG expression groups; *p* < 0.05 was considered statistically significant. Considering GBM patients characterized by G-CIMP signature have a better survival. We conducted survival analysis excluding the G-CIMP positive patients.

### Tissue microarray analysis

We performed immunohistochemical staining for *CBX3* on two tissue microarrays, namely, a glioblastoma tissue microarray (GLC-1601; Servicebio, consisting of 60 GBM and 10 NG tissues) and an astrocytoma tissue microarray (ASC-1501; Servicebio, consisting of 36 A and 27 matched adjacent normal tissues; see Additional file 1: Table S1).

### Immunohistochemical staining

Immunohistochemistry was performed as previously described [35] with a mouse antibody against CBX3 (1:100; Santa Cruz; sc-398562). CBX3 staining was evaluated by two pathologists who were blinded to the sample types. CBX3 staining in the tissue sections was assessed using a widely accepted German semiquantitative scoring system [36]. Each sample was assigned a score according to the nuclear staining intensity (no staining = 0; weak staining = 1, moderate staining = 2, and strong staining = 3) and the extent of positive-stained cells (0–5% = 0, 5–25% = 1, 26–50% = 2, 51–75% = 3, and 76–100% = 4). The final immunoreactivity score was obtained by multiplying the intensity score by the extent score and ranged from 0 to 12. The samples were divided into three expression groups based on the final immunoreactivity score, as follows: low (0–7), medium (8–10), and high (11, 12).

### Cell culture

The human glioblastoma cell lines A172, U-118MG, and U-87MG were purchased from the cell bank of the Chinese Academy of Sciences in Shanghai, and SF-268 was purchased from American Tissue Culture Collection. U373 and U251 were obtained as gifts from Prof. Yiping Li (Institute of Human Virology, Zhongshan School of Medicine, Sun Yat-sen University North Campus). All cell lines were maintained in DMEM supplemented with 10% FBS, 100 μg/ml penicillin, and 100 μg/ml streptomycin, except SF-268, which was maintained in RPMI 1640 medium. Cells were incubated in a humidified atmosphere containing 5% CO_2_ at 37 °C.

### Tissue collection

A total of 45 glioma surgical specimens and 3 NG tissues (from brain trauma decompression) were collected from patients undergoing surgical procedures at the Union Hospital of Tongji Medical College, China (Additional file 1: Table S1).

### Quantitative polymerase chain reaction (qPCR) of tissues and cell lines

Total RNA was extracted by Trizol reagent (Aidlab) according to the manufacturer’s instructions. cDNA samples were reverse transcribed from total RNA of glioma surgical specimens and glioblastoma cell lines. The amplification program used was as follows: 50 °C for 2 min, 95 °C for 10 min, followed by 40 cycles at 95 °C for 30 s and 60 °C for 30 s. The relative expression of *CBX3* was determined by the 2^−ΔΔCt^ method with *GAPDH* as an internal control. The primer sequences are listed in Additional file 1: Table S2.

### Small interfering RNA transfection

The lentiviral vector containing *CBX3* siRNA was synthesized by Genechem (Shanghai, China). siRNA target sequences (shCBX3-1, 5′-ACGTGTAGTGAATGGGAAA-3′ and shCBX3-2, 5′-TGAAGAATTTGTCGTGGAA-3′) for the CBX3 gene (NM_016587) were designed, and a nonsilencing siRNA sequence (5′-TTCTCCGAACGTGTCACGT-3′) was adopted as a negative control (NC, shCtrl).

U373 cells were seeded in six-well culture plates and transfected with lentivirus according to the manufacturer’s instructions (MOI = 5). The culture medium was replaced after 10 h, and mCherry expression was observed under a fluorescence microscope (Olympus) 3 days after infection.

### Western blot analysis

Western blotting was performed as previously described [37]. Blots were probed with anti-CBX3 (Santa Cruz, USA) and anti-β-actin (Servicebio, China) antibodies.

### CCK8 assay

U373 cells were seeded in 96-well plates at a density of 3000 cells/well and incubated overnight. Cell proliferation was determined at 24, 48, 96, and 120 h by measuring the absorbance at 450 nm according to the manufacturer’s protocol.

### Cell cycle assay

Cells were fixed in precooled 70% ethanol for 4 h and then resuspended by adding 400 µl 7-amino-actinomycin D (7-AAD) (50 μg/ml) and 100 μl RNase (50 μg/ml). The DNA content was analyzed by flow cytometry using a FACSCalibur (BD Biosciences). The percentage of cells in each phase of the cell cycle was determined using the ModFit LT program (Verity Software House, USA).

### Statistical analysis

The significance of the differences between the groups was determined with a Kruskal–Wallis *H* test or Student’s t test, and p-values less than 0.05 were considered statistically significant. The measurement data are expressed as the means ± standard deviations. The results were repeated in at least three independent experiments. The Kaplan–Meier survival curves were plotted using GraphPad Prism 5, which enables the interactive exploration of survival correlations using a log-rank test. Receiver operating characteristic (ROC) curve analysis was performed to evaluate the diagnostic efficiency. SPSS v19.0 was used for statistical analysis.

## Results

### Dataset characteristics

A total of 31 studies satisfying the inclusion criteria and containing 1277 GBM, 427 A, 189 OD, and 150 NG samples were analyzed. The detailed characteristics of the four comparison groups (GBM vs. NG training set, GBM vs. NG validation set, GBM vs. A set, and GBM vs. OD set) are summarized in Table 1. Since GSE68928, GSE4271, and GSE4412 datasets have two different platforms, we devided each dataset into two datasets respectively.

### Integrated analysis of nine training datasets identifies 322 DEGs in GBM vs. NG

**Fig. 2:**
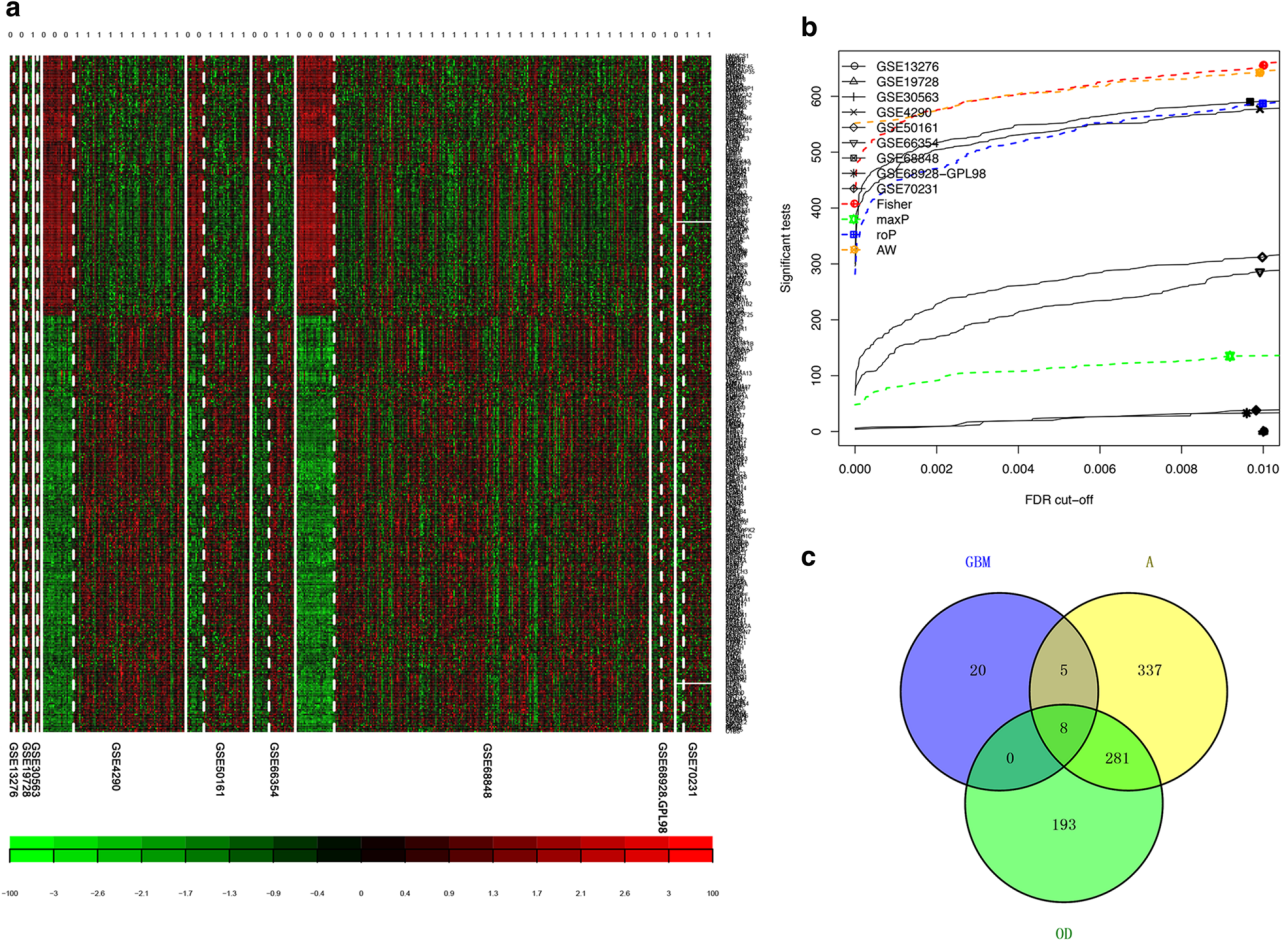
Identification of DEGs specifically expressed in GBM. **a** Clustering analyses were initially performed with the nine datasets in GBM vs. NG tissues. Each row represents the expression level of a mRNA, and each column represents a sample. “1” = glioblastoma, “0” = NG tissues. **b** The number of DEGs with the four different meta-analysis algorithms (maxP, fisher, roP and AW). **c** Venn diagram depicting the number of mRNAs that overlapped in GBM vs. NG, GBM vs. A, and GBM vs. NG tissues. GBM: glioblastoma; A: astrocytoma; OD: oligodendroglioma; NG: nonglioma; DEG: differentially expressed gene

We applied two meta-analysis methods to identify DEGs in GBM as described in “Materials and methods” section. By combining p-values, 437 DEGs were detected using Fisher’s sum of logs method (Fig. 2a, b). To further refine the list of DEGs in GBM, we conducted a random effects model to estimate the differences in gene expression across all datasets by combining the individual effect sizes into a meta-effect size [38, 39].

This method identified 393 DEGs at an FDR threshold of 1 × 10^−19^. Finally, the DEGs obtained from both methods were plotted in a Venn diagram, revealing 322 overlapping DEGs when combining both p-values and effect sizes. This overlapped group contained genes that not only had an overall large effect size across all datasets but also were significantly differentially expressed. To reveal biological functions differentially regulated in GBM, all 322 DEGs were analyzed by using Enrichr. Results for enriched biological pathways and gene ontology are shown in Additional file 1: Table S3. Pathways in cancer, MAPK signaling pathway, Wnt signaling pathway, apoptosis, and cell cycle were enriched pathway terms in GBM vs. NG.

### Integrated analysis of the training and validation datasets identifies 33 DEGs in GBM vs. NG

A total of 471 DEGs were detected by combining p-values using Fisher’s sum of logs method (Additional file 2: Figure S1A). By using a random effects model to combine effect sizes, 285 DEGs were identified. Finally, the DEGs obtained from both methods were plotted in a Venn diagram, revealing 188 overlapping DEG when combining p-values and effect sizes.

In our study, we adopted the training-validation approach [10], using the larger dataset (nine datasets) as the training set and the smaller dataset (seven datasets) as the validation set. Venn diagram analysis showed that 33 DEGs, including 28 upregulated DEGs and 5 downregulated DEGs, were significantly expressed in both the training and validation datasets.

However, because only GBM samples were included in this meta-analysis, these 33 genes possibly also abnormally expressed in other subtypes of glioma relative to their expression in normal tissue. Therefore, we sought to determine whether this 33-DEG signature was specific for GBM or whether this set of genes was also significantly differentially expressed in other glioma subtypes.

### Integrated analysis of GBM vs. NG, GBM vs. A, and GBM vs. OD sets identifies 8 DEGs significantly and specifically expressed in GBM

We analyzed additional validation datasets, including 21 datasets containing a total of 852 GBM and 427 A samples (Additional file 2: Figure S1B). Finally, we found that a total of 920 genes were significantly differentially expressed as assessed by Fisher’s sum of logs method (FDR < 0.05).

We analyzed additional validation datasets, including 12 datasets containing a total of 590 GBM and 189 OD samples (Additional file 2: Figure S1C). Finally, we found that a total of 482 genes were significantly differentially expressed as assessed by Fisher’s sum of logs method (FDR < 0.05).

In summary, the Venn diagrams of the three integrated analyses revealed eight genes that are significantly and consistently differentially expressed in GBM (six upregulated and two downregulated) and could distinguish GBM samples from NG tissues or tissues of other glioma subtypes (Fig. 2c, Additional file 1: Table S4). Thus, these genes may be potential diagnostic and therapeutic targets in GBM.

### Validation in the TCGA-GBMLGG and CGGA cohorts

**Fig. 3:**
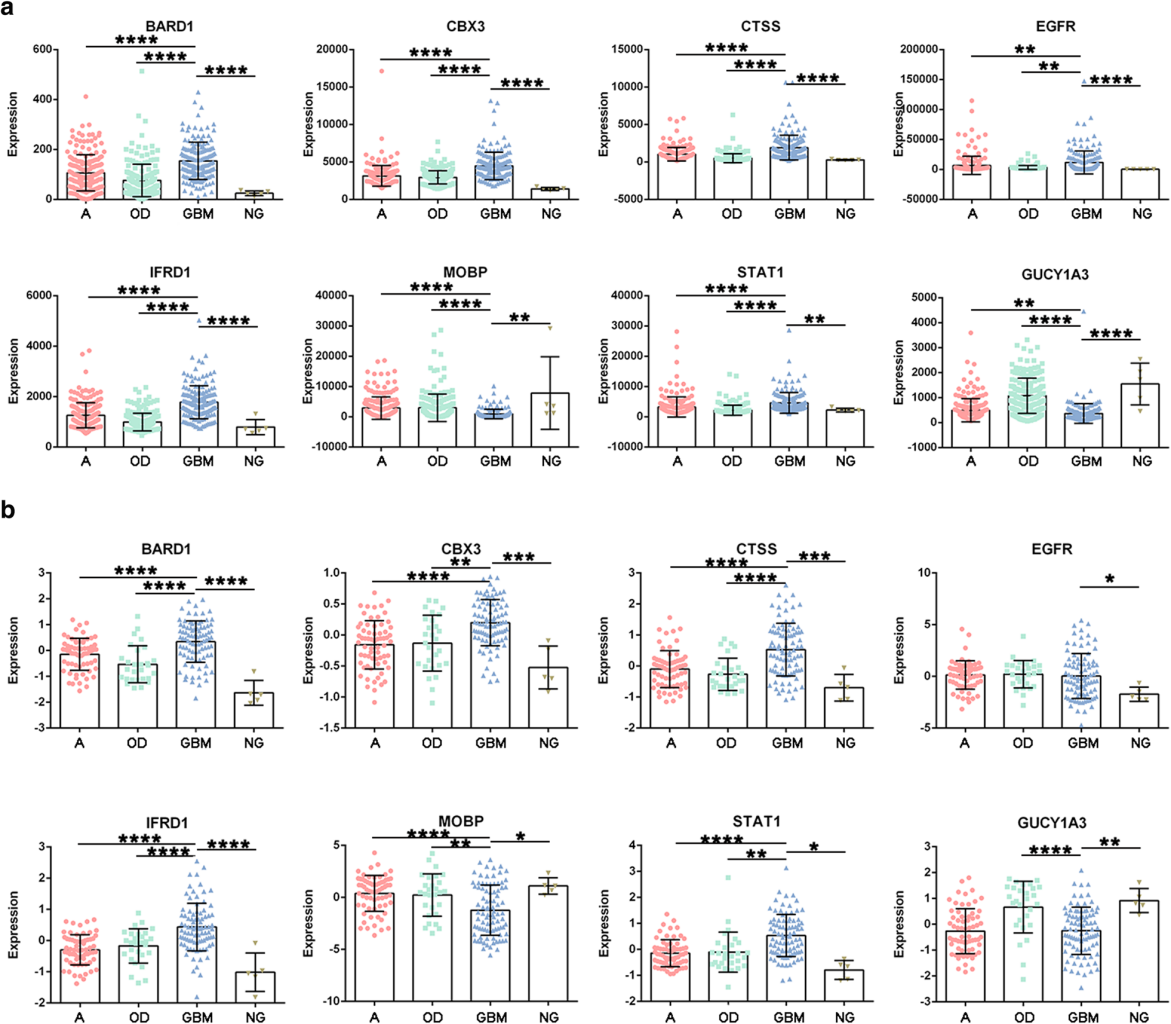
Validation of the TCGA-GBMLGG and CGGA datasets. Expression levels of the eight DEGs in GBM, A, OD, and NG tissues in the TCGA-GBMLGG (**a**) and CGGA (**b**) cohorts. GBM: glioblastoma; A: astrocytoma; OD: oligodendroglioma; NG: nonglioma; DEG: differentially expressed gene; TCGA: The Cancer Genome Atlas; CGGA: the Chinese Glioma Genome Atlas. ****Indicates a p-value of < 0.0001; **indicates a p-value of < 0.01; *indicates a p-value of < 0.05

We further validated the eight DEGs in the TCGA-GBMLGG (158 GBM, 193 A, 188 OD, and 5 NG) and CGGA cohorts (89 GBM, 66 A, 28 OD, and 5 NG). In these two validation cohorts, all eight DEGs, including six upregulated and two downregulated genes, were significantly differentially expressed in GBM vs. NG (Fig. 3, Additional file 1: Tables S5 and S6). BRCA1 associated RING domain 1 (*BARD1*), *CBX3*, cathepsin S (*CTSS*), interferon-related developmental regulator 1 (*IFRD1*), signal transducer and activator of transcription 1 (*STAT1*), and myelin-associated oligodendrocytic basic protein (*MOBP*) were significantly and specifically differentially expressed in GBM.

Further ROC curve analysis based on the upregulated DEGs in the TCGA-GBMLGG cohort revealed that *BARD1*, *CBX3*, *CTSS*, *IFRD1*, and *STAT1* had high accuracy in differentiating GBM samples from NG, A, and OD samples. In the CGGA cohort, *BARD1*, *CBX3*, *CTSS*, *IFRD1*, and *STAT1* were similarly shown to have high accuracy in differentiating GBM samples from NG, A, and OD samples (Additional file 3: Figure S2).

### Survival analysis

**Fig. 4:**
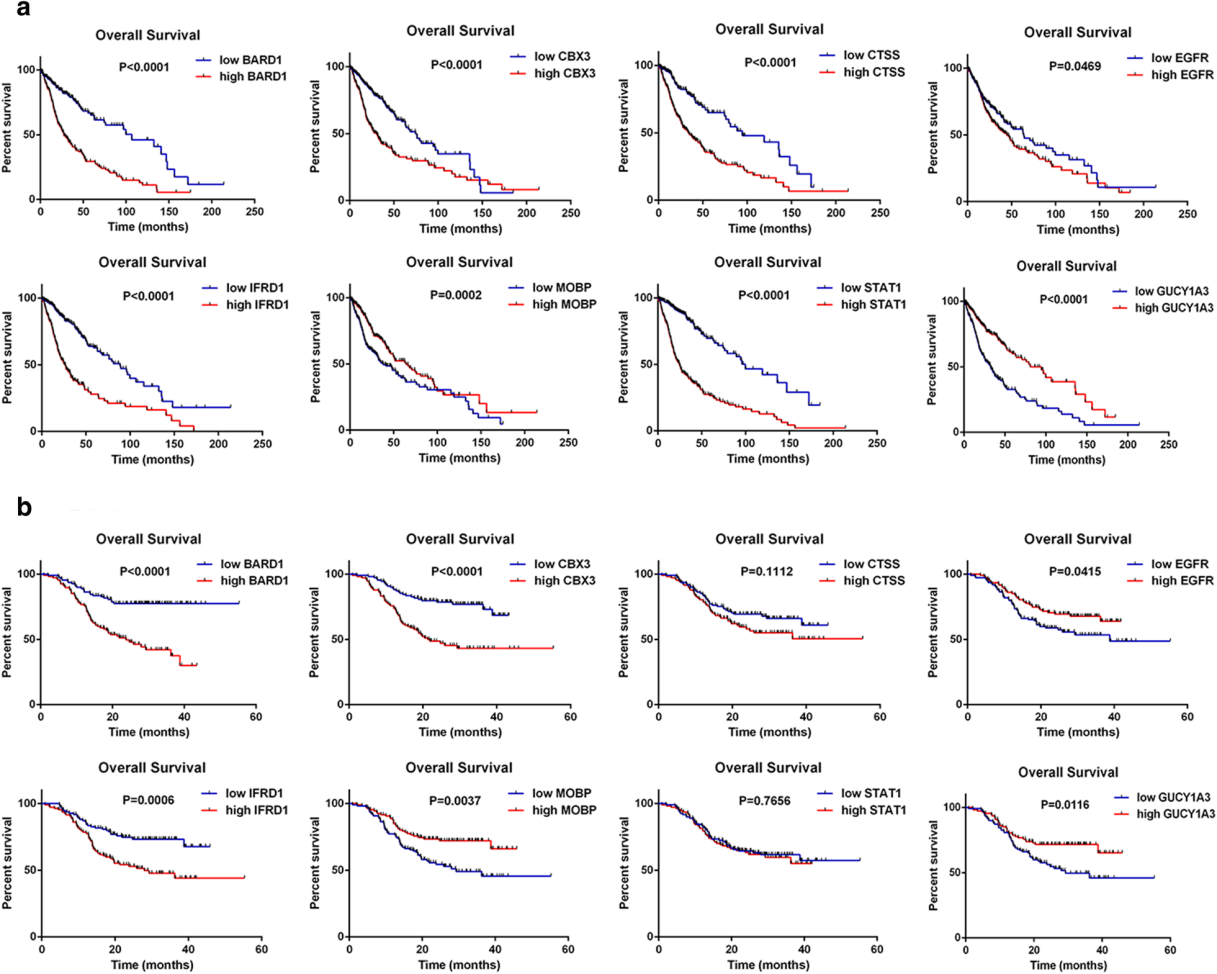
Survival analysis of patients with glioma. Kaplan–Meier analyses were performed based on the median expression levels of the eight DEGs in the TCGA-GBMLGG (**a**) and CGGA (**b**) cohorts. The tick marks on the Kaplan–Meier survival curves represent the censored subjects. TCGA: The Cancer Genome Atlas; CGGA: the Chinese Glioma Genome Atlas

Further, to investigate the clinical relevance of CBX3 expression in glioma, Kaplan–Meier analysis was conducted to explore whether these eight genes play roles in the survival of patients with glioma. In the TCGA-GBMLGG cohort, the results showed that patients with high *CBX3*, *BARD1*, *CTSS*, epidermal growth factor receptor (*EGFR*), *IFRD1*, or *STAT1* expression had significantly shorter survival but patients with high guanylate cyclase 1 soluble subunit alpha 3 (*GUCY1A3*) or *MOBP* expression had significantly longer survival than patients with lower expression of these genes. In the CGGA cohort, the results showed that patients with high *CBX3*, *BARD1*, *EGFR*, or *IFRD1* expression had significantly shorter survival but patients with high *GUCY1A3* or *MOBP* expression had significantly longer survival than patients with lower expression of these genes (Fig. 4). After excluding G-CIMP positive patients in TCGA-GBMLGG, survival analysis showed that patients with high CBX3, BARD1, CTSS, STAT1, or IFRD1 expression had significantly shorter survival but patients with high GUCY1A3 or MOBP expression had significantly longer survival than patients with lower expression of these genes we conducted Kaplan–Meier analysis (Additional file 4: Figure S3).

### *CBX3* overexpression in human GBM samples was shown by immunohistochemical staining and qPCR

**Fig. 5:**
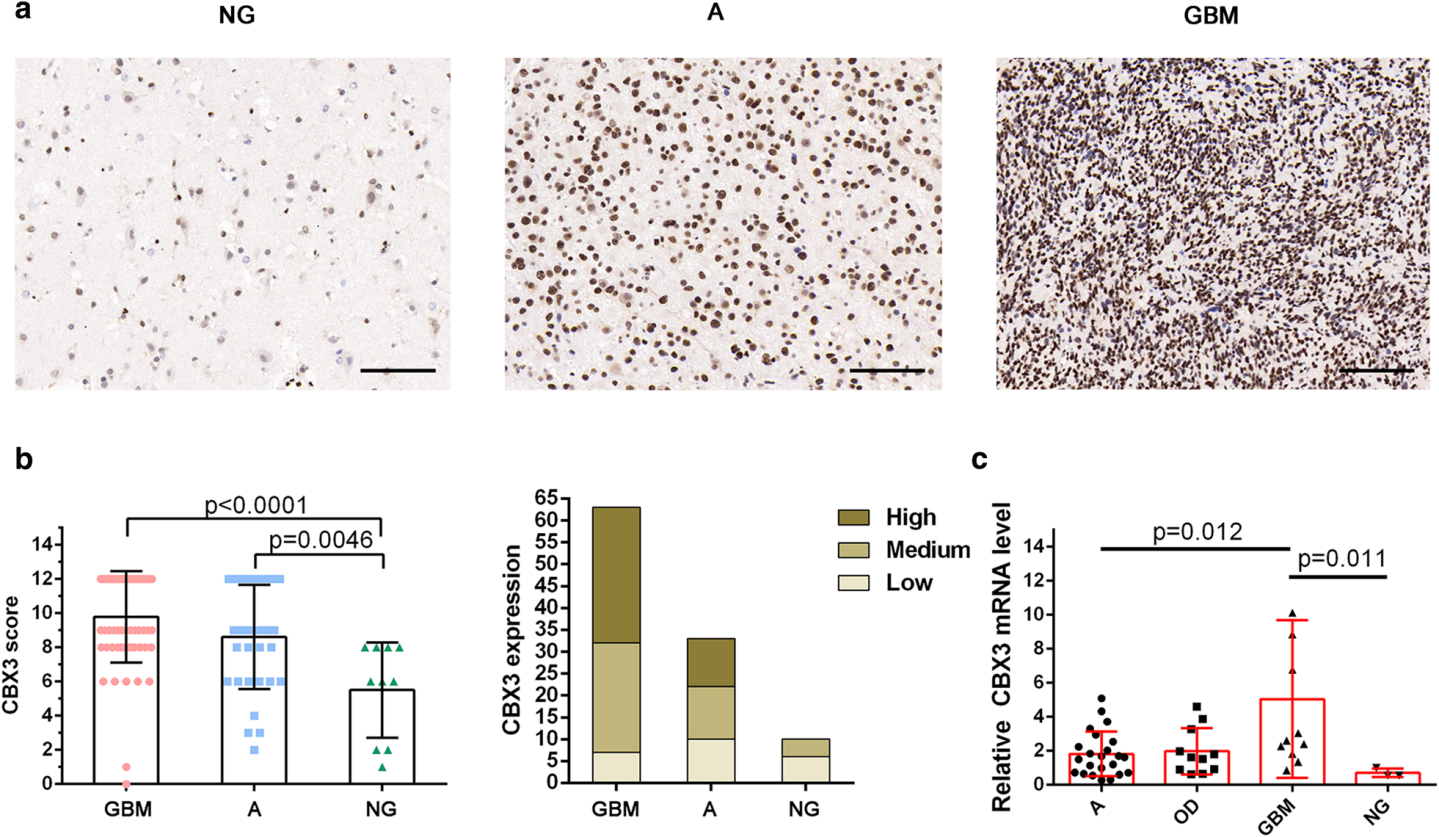
CBX3 is overexpressed in GBM tissues. **a** Representative images of CBX3 staining in NG, A, and GBM tissues (Magnification bar = 100 μm). **b** CBX3 scores for GBM, A, and NG in tissue microarrays and the distribution of high, medium, and low CBX3 expression in the three tissues. **c** CBX3 mRNA expression in GBM, A, and NG tissues from surgical specimens. GBM: glioblastoma; A: astrocytoma; OD: oligodendroglioma; NG: nonglioma

Among these eight DEGs, *CBX3* has been revealed to be linked with cancers; however, the precise role of *CBX3* in glioma remains unclear, so we focused further investigation on *CBX3*. CBX3 expression was assessed by immunohistochemical staining. Two tissue microarrays consisting of 60 GBM, 36 A, and 10 NG samples were used to determine CBX3 expression. Immunohistochemical analysis revealed that CBX3 expression was significantly upregulated in the nuclei in GBM and A tissues compared with that in normal brain tissue. However, no significant difference was observed between GBM and A samples, possibly due to the limited sample size. The samples were divided into three groups according to the CBX3 expression score, representing low (0–7), medium (8–10), and high (11, 12) expression levels of CBX3 (Fig. 5a, b).

Furthermore, the expression of *CBX3* mRNA in surgical specimens was detected by qPCR. The qPCR results in the surgical specimens showed that *CBX3* mRNA expression was highest in GBM tissue and that *CBX3* mRNA expression in GBM tissue was different from that in A or NG brain tissue. However, no statistically significant difference was observed between GBM and OD tissues (Fig. 5c).

**Fig. 6:**
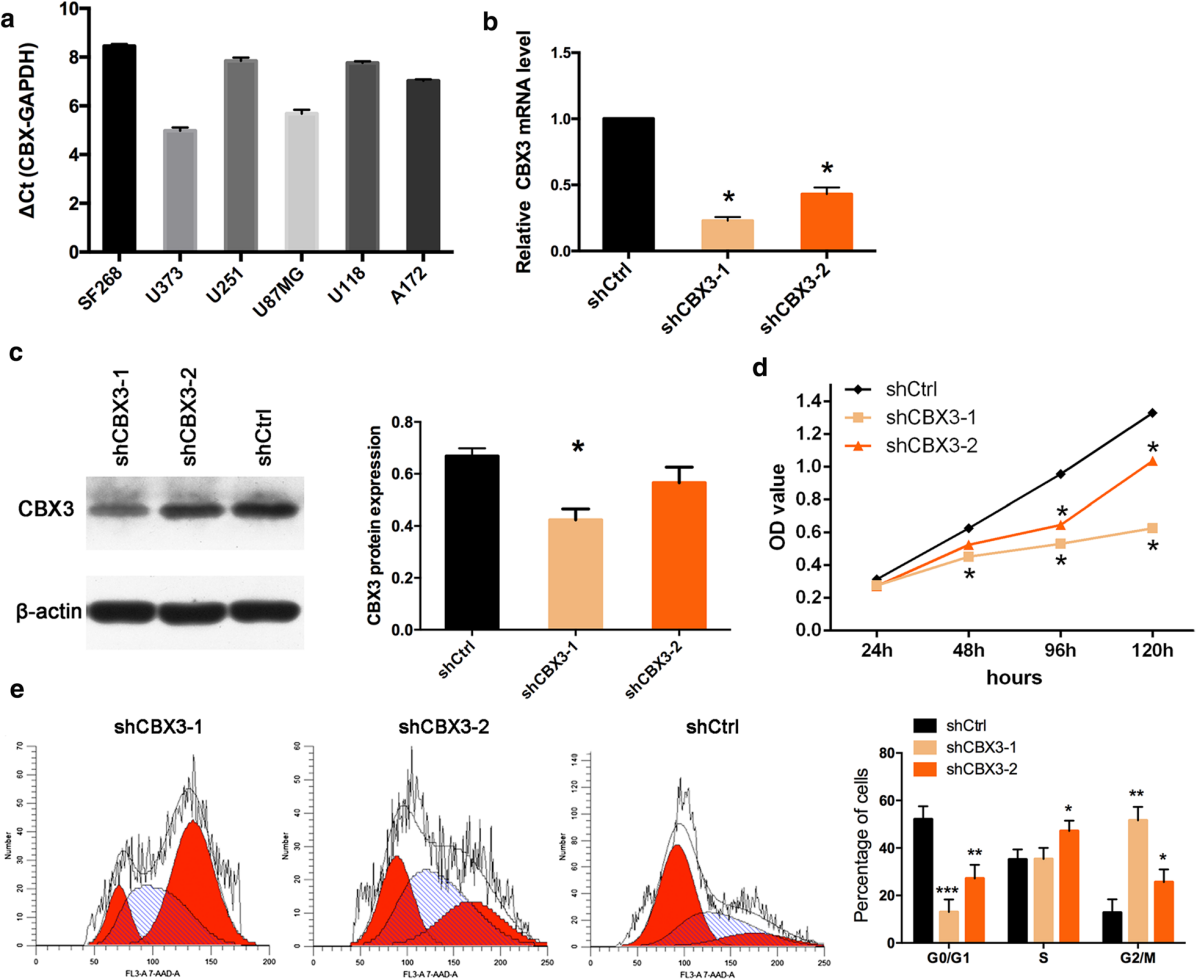
Knockdown of CBX3 inhibits cell growth and leads to G2/M cell cycle arrest in U373 cells. **a** qPCR analysis of CBX3 mRNA levels in SF268, U373, U251, U87MG, U118, and A172 cells. qPCR (**b**) and Western blot (**c**) validation of CBX3 expression in U373 cells transfected with shCBX3-1, shCBX3-2, and shCtrl. **d** CCK8 proliferation curve of U373 cells transfected with shCBX3-1, shCBX3-2, and shCtrl. **e** The cell cycle was analyzed by flow cytometry in U373 cells transfected with shCtrl, shCBX3-1, and shCBX3-2. **Indicates a p-value of < 0.01; *indicates a p-value of < 0.05

CBX3 mRNA expression was evaluated by qPCR in glioblastoma cell lines, including SF268, U373, U251, U87MG, U118, and A172 cells (Fig. 6a). Among these cell lines, U373 cells displayed the highest expression of *CBX3* and were thus selected for the following studies.

### Knockdown of *CBX3* inhibits U373 cell growth

To explore the role of *CBX3* in glioblastoma, either siRNA targeting *CBX3* or nonsilencing RNA sequences were transfected into U373 cells. Approximately 72 h after virus transfection, 90% of the U373 cells exhibited red fluorescence under fluorescence microscopy, indicating CBX3 expression. qPCR analysis showed that the expression of *CBX3* mRNA was reduced by approximately 77% in the shCBX3-1 group compared with that in the shCtrl group (Fig. 6b). Moreover, Western blot analysis suggested that the expression of the CBX3 protein was downregulated in the shCBX3-1 group compared to that in cells transfected with the control lentivirus (Fig. 6c).

To determine the effects of *CBX3* on glioblastoma cell growth, we monitored proliferation using the CCK8 assay. The proliferation rate of U373 cells transfected with shCBX3-1 was markedly lower than that of cells transfected with shCtrl (Fig. 6d).

### Knockdown of *CBX3* induced G2/M cell cycle arrest in U373 cells

The cell cycle distribution in cells infected with either shCBX3 or shCtrl lentivirus was explored in an attempt to explain the CBX3-mediated suppression of proliferation. The number of CBX3 knockdown U373 cells in the G0/G1 phase was significantly lower than the number of control cells in the G0/G1 phase, while the number of CBX3 knockdown U373 cells in the G2/M phase was markedly higher than the corresponding number of control cells. Thus, U373 cells exhibited G2/M cell cycle arrest after transfection with shCBX3-1 (Fig. 6e).

## Discussion

Many transcriptional studies in glioma have been performed; however, most used limited sample sizes, variable platforms and different sample types (cell or tissue), making it challenging to characterize stable and reliable molecular biomarkers of glioma. To our knowledge, our study is a very large integrated analysis to date of gene expression in glioblastoma. Eight genes were consistently expressed between GBM tissues and NG, A or OD tissues with high significance, a finding that may ultimately translate into clinical practice. To further assess our results, we investigated the role of these eight genes in the survival of patients with glioma using TCGA-GBMLGG and CGGA cohorts. Patients with high *CBX3*, *BARD1*, *EGFR*, or *IFRD1* expression had significantly shorter survival and patients with high *GUCY1A3* or *MOBP* expression had significantly longer survival than patients with lower expression of these genes.

Some of these eight genes, such as *EGFR*, *STAT1*, and *BARD1*, have been confirmed to be involved in cancer by numerous studies. Furthermore, some of these genes, such as *MOBP* and *CTSS*, have been confirmed to be related to glioma. *CBX3*, *GUCY1A3*, and *IFRD1* have been reported in relation to some tumors but have been studied little in glioma.

*MOBP* is specifically overexpressed in oligodendrocytes [40]. Our study found that in the GBM vs. OD comparison, *MOBP* was overexpressed in OD, consistent with the literature. Thomas et al. [41] showed by an ELISA that CTSS was highly expressed in glioblastoma but was expressed at relatively low levels in grade I-III astrocytoma. In addition, high *CTSS* expression in glioblastoma shows a poor prognosis. This conclusion is completely consistent with our findings. *GUCY1A3* is an upstream regulatory gene of *VEGF* and may be a molecular target for antiangiogenic therapy in glioma [42]. *IFRD1* is expressed in various cells and may play a role in promoting tissue proliferation or regeneration [43].

However, research on *IFRD1* in glioma has not been reported. Studies by Lewis and others showed that *IFRD1* is highly expressed in colon cancer and is negatively correlated with the 5-year survival time. In our study, *IFRD1* is specifically overexpressed in glioblastoma and suggests a poor prognosis in glioma.

In our study, *CBX3* was upregulated in glioblastoma, and patients with high *CBX3* expression had shorter survival. Considering that there is little research about *CBX3* in gliomas, we selected *CBX3* for further study. The mammalian HP1 family contains three isoforms, HP1a (CBX5), HP1b (CBX1), and HP1γ (CBX3). The CBX3-encoded protein HP1γ, a member of the heterochromatin family, is a highly conserved nonhistone chromatin protein containing two highly conserved domains. Current studies have confirmed that *CBX3* is involved in transcriptional silencing, DNA repair, and RNA splicing. Moreover, the mechanisms of action of *CBX3* in cancer remain obscure.

Han et al. [24] found that *CBX3* was positively expressed in 90.3% of non-small cell lung cancer tissues, whereas only 2 of 7 normal lung tissues were positive for *CBX3* expression. Saini et al. [25] identified that *CBX3* can be used as a marker for tumor stem cells in osteosarcoma and that *CBX3* was overexpressed in osteosarcoma and osteosarcoma metastases to the lung compared with its expression in primary osteoblasts. Liu et al. [27] found that *CBX3* is overexpressed in colorectal cancer, while miR-30a is downregulated and inversely correlated with high *CBX3* expression. Furthermore, *CBX3* promotes colorectal cancer cell proliferation and tumorigenesis. p21 is a cyclin-dependent kinase inhibitor that can interrupt cell cycle progression, leading to cell cycle arrest [44, 45]. Knockdown of *CBX3* increased p21 expression, resulting in slower proliferation of colorectal cancer cells. The miR-30a/CBX3/p21 axis is proposed to regulate the development of colorectal cancer and to be a prognostic and therapeutic target. Fan et al. [46] demonstrated that *CBX3* can promote proliferation and cell cycle progression both in vivo and in vitro in colon cancer cells. *CBX3* can promote the formation of colon cancer by inhibiting the expression of *CDK6/p21*, which are cell cycle (G1 phase to S phase) related genes. Several studies have also found that HP1 proteins interact with transcriptional regulators of key cell cycle genes, including *cyclin E*, *E2F1*, and *p53* [47–49]. In our study, *CBX3* knockdown inhibited the proliferation of glioblastoma cells and led to cell cycle arrest at the G2/M phase to G0/G1 phase boundary, partly in accordance with the findings in the above studies.

However, there were also some limitations that should be strengthened in this study. First, since datasets in this study were from 15 different platforms, the batch effect is large, but we did quality control before DEG analysis to reduce the effect. Second, the functional analysis in wet experiment only explored CBX3, and further analysis in other gene were still needed.

## Conclusions

In summary, this study is a global analysis identifying glioblastoma-specific mRNAs in such a large sample size through integrated analysis. Our analysis uses a “prevalidation” integrated analysis to identify signatures and wet lab experiments to validate the identified CBX3 gene, which may accelerate translational research and will provide insight into new strategies to seek tumor biomarkers for precision oncology.

## Additional files

**Additional file 1: Table S1.** Baseline characteristics of patients with glioma from tissue microarray and Union hospital cohort. **Table S2.** Primer sequences used for qPCR. **Table S3.** Functional analysis results. **Table S4.** The overlapped 8 DEGs identified in the integrated analysis of GBM vs. NG, GBM vs. A, and GBM vs. OD tissues. **Table S5.** Eight differentially expressed genes validation results in TCGA-GBMLGG dataset. **Table S6.** Eight differentially expressed genes validation results in CGGA dataset.

**Additional file 2: Figure S1.** Clustering analyses. (A) Clustering analyses performed with 7 datasets in the validation cohorts of GBM vs NG. (B) Clustering analyses performed with 21 datasets in the validation cohorts of GBM vs A. (C) Clustering analyses performed with 12 datasets in the validation cohorts of GBM vs OD. Abbreviations: GBM, glioblastoma; NG, nonglioma; A, astrocytoma; OD, oligodendroglioma.

**Additional file 3: Figure S2.** ROC analysis in the TCGA-GBMLGG and CGGA datasets. Expression levels of the six upregulated DEGs in GBM vs. NG (A), GBM vs. A (B), and GBM vs. OD (C) tissues in the TCGA-GBMLGG cohort. Expression levels of the six upregulated DEGs in GBM vs. NG (D), GBM vs. A (E), and GBM vs. OD (F) tissues in the CGGA cohort. Abbreviations: TCGA, The Cancer Genome Atlas; CGGA, the Chinese Glioma Genome Atlas; GBM, glioblastoma; NG, nonglioma; A, astrocytoma; OD, oligodendroglioma.

**Additional file 4: Figure S3.** Survival analysis results in TCGA-GBMLGG and CGGA datasets excluding G-CIMP positive patients. Kaplan-Meier analyses were performed based on the median expression levels of the eight DEGs in the TCGA-GBMLGG (A) and CGGA (B) cohorts. The tick marks on the Kaplan-Meier survival curves represent the censored subjects. Abbreviations: TCGA, The Cancer Genome Atlas; CGGA, the Chinese Glioma Genome Atlas.

## Data Availability

The data supporting our findings can be found in the additional data.

## Abbreviations

GBM: glioblastoma
GEO: Gene Expression Omnibus
CBX3: chromobox homolog 3
HP1: heterochromatin protein 1
DEG: differentially expressed gene
TCGA: The Cancer Genome Atlas
CGGA: Chinese Glioma Genome Atlas
NG: nonglioma
A: astrocytoma
OD: oligodendroglioma
OA: oligoastrocytoma
FDR: false discovery rate
GO: Gene Ontology
KEGG: Kyoto Encyclopedia of Genes and Genomes
DAVID: Database for Annotation, Visualization, and Integrated Discovery
BP: biological process
CC: cellular component
MF: molecular function
qPCR: quantitative polymerase chain reaction
7-AAD: 7-amino-actinomycin D
ROC: receiver operating characteristic
BARD1: BRCA1 associated RING domain 1
CTSS: cathepsin S
IFRD1: interferon-related developmental regulator 1
STAT1: signal transducer and activator of transcription 1
MOBP: myelin-associated oligodendrocytic basic protein
EGFR: epidermal growth factor receptor
GUCY1A3: guanylate cyclase 1 soluble subunit alpha 3
