# Supplementary material for: Integrated analysis of 34 microarray datasets reveals CBX3 as a diagnostic and prognostic biomarker in glioblastoma
Source: J Transl Med. 2019 May 28;17:179. doi: 10.1186/s12967-019-1930-3 (PMC6540543; doi:10.1186/s12967-019-1930-3)
Supplement: Supplementary file 1 — Additional file 1: Table S1. Baseline characteristics of patients with glioma from tissue microarray and Union hospital cohort. Table S2. Primer sequences used for qPCR. Table S3. Functional analysis results. Table S4. The overlapped 8 DEGs identified in the integrated analysis of GBM vs. NG, GBM vs. A, and GBM vs. OD tissues. Table S5. Eight differentially expressed genes validation results in TCGA-GBMLGG dataset. Table S6. Eight differentially expressed genes validation results in CGGA dataset. [file 12967_2019_1930_MOESM1_ESM.docx]

**Additional Material**

**Integrated analysis of 33 microarray datasets reveals CBX3 as a diagnostic and prognostic biomarker in glioblastoma**

**Authors:** Siqi Wang, Fang Liu, Yuhui Wang, Wenliang Fan, Hongyang Zhao, Liying Liu, Chunyuan Cen, Xiaobin Jiang, Min Sun*, Ping Han*

**Corresponding Author:**

E-mail addresses: Email: [cjr.hanping@vip.163.com](mailto:cjr.hanping@vip.163.com) (P. Han), [sunmin-0715@163.com](mailto:sunmin-0715@163.com) (M. Sun).

* Contributed equally.

**Table S1.** Baseline characteristics of patients with glioma from tissue microarray and Union hospital cohort.

|  | **tissue microarray (n=106)** | **cohort from Union hospital (n=48)** |
| --- | --- | --- |
| Gender |  |  |
| Male | 40 | 25 |
| Female | 66 | 23 |
| Age |  |  |
| 50 years or younger | 47 | 27 |
| older than 50 years | 59 | 21 |
| histological type |  |  |
| A | 33 | 21 |
| OD | 0 | 11 |
| GBM | 63 | 13 |
| NG | 10 | 3 |
| stage |  |  |
| I | 0 | 1 |
| II | 18 | 12 |
| III | 15 | 19 |
| IV | 63 | 13 |
| NG | 10 | 3 |

**Table S2.** Primer sequences used for qPCR.

| Primer | Sequence |
| --- | --- |
| GAPDH-F | TCAAGAAGGTGGTGAAGCAGG |
| GAPDH-R | TCAAAGGTGGAGGAGTGGGT |
| CBX3-F | GAGATGCTGCTGACAAACCA |
| CBX3-R | TATTTGCCTCTTTCGCCAGC |

**Table S3**. **Functional analysis results.**

| **Term** | **P-value** | **FDR** | **Z-score** | **Combined Score** |
| --- | --- | --- | --- | --- |
| **GO Biological Process** | | | | |
| positive regulation of transcription, DNA-templated (GO:0045893) | 3.00E-14 | 7.07E-11 | -1.716055 | 53.432301 |
| positive regulation of gene expression (GO:0010628) | 6.93E-14 | 8.17E-11 | -1.669825 | 50.595088 |
| regulation of transcription, DNA-templated (GO:0006355) | 2.28E-11 | 1.79E-08 | -1.835439 | 44.977451 |
| positive regulation of transcription from RNA polymerase II promoter (GO:0045944) | 9.87E-11 | 5.81E-08 | -1.893466 | 43.624272 |
| regulation of transcription from RNA polymerase II promoter (GO:0006357) | 2.00E-10 | 9.42E-08 | -1.373197 | 30.667602 |
| positive regulation of nucleic acid-templated transcription (GO:1903508) | 7.94E-10 | 3.12E-07 | -1.998726 | 41.881412 |
| anterograde trans-synaptic signaling (GO:0098916) | 1.43E-08 | 4.81E-06 | -1.20416 | 21.751689 |
| protein phosphorylation (GO:0006468) | 5.35E-08 | 1.58E-05 | -1.092443 | 18.290503 |
| chemical synaptic transmission (GO:0007268) | 2.68E-07 | 7.01E-05 | -1.344805 | 20.350954 |
| response to cytokine (GO:0034097) | 4.05E-07 | 9.03E-05 | -1.275354 | 18.771262 |
| nervous system development (GO:0007399) | 4.22E-07 | 9.03E-05 | -1.316865 | 19.330429 |
| regulation of cell proliferation (GO:0042127) | 1.30E-06 | 0.0002554 | -1.123012 | 15.219538 |
| positive regulation of cell proliferation (GO:0008284) | 1.70E-06 | 0.000308 | -1.548352 | 20.569986 |
| transmembrane receptor protein tyrosine kinase signaling pathway (GO:0007169) | 2.09E-06 | 0.0003284 | -1.721575 | 22.514831 |
| regulation of gene expression (GO:0010468) | 2.05E-06 | 0.0003284 | -1.855234 | 24.302527 |
| regulation of apoptotic process (GO:0042981) | 3.42E-06 | 0.0005035 | -1.767232 | 22.242343 |
| positive regulation of protein phosphorylation (GO:0001934) | 3.88E-06 | 0.0005378 | -1.881035 | 23.436885 |
| phosphorylation (GO:0016310) | 5.20E-06 | 0.0006803 | -1.697481 | 20.653932 |
| transcription, DNA-templated (GO:0006351) | 5.90E-06 | 0.0007311 | -1.853267 | 22.315644 |
| positive regulation of phosphorylation (GO:0042327) | 7.90E-06 | 0.0008864 | -1.915052 | 22.499165 |
| negative regulation of cell proliferation (GO:0008285) | 7.78E-06 | 0.0008864 | -1.838435 | 21.627952 |
| cytokine-mediated signaling pathway (GO:0019221) | 1.37E-05 | 0.0014626 | -1.326294 | 14.856139 |
| transcription from RNA polymerase II promoter (GO:0006366) | 1.44E-05 | 0.0014717 | -1.184687 | 13.209928 |
| interferon-gamma-mediated signaling pathway (GO:0060333) | 1.82E-05 | 0.001653 | -1.295706 | 14.138439 |
| cellular response to cytokine stimulus (GO:0071345) | 1.81E-05 | 0.001653 | -1.147343 | 12.531576 |
| negative regulation of transcription from RNA polymerase II promoter (GO:0000122) | 1.72E-05 | 0.001653 | -1.069312 | 11.73084 |
| negative regulation of transcription, DNA-templated (GO:0045892) | 2.30E-05 | 0.0020049 | -1.722769 | 18.400935 |
| activation of phospholipase C activity (GO:0007202) | 2.78E-05 | 0.0021425 | -2.210505 | 23.188924 |
| regulation of MAPK cascade (GO:0043408) | 2.82E-05 | 0.0021425 | -1.351536 | 14.159356 |
| regulation of signal transduction (GO:0009966) | 2.81E-05 | 0.0021425 | -1.314602 | 13.77441 |
| negative regulation of gene expression (GO:0010629) | 2.59E-05 | 0.0021425 | -1.233018 | 13.024424 |
| positive regulation of neuron differentiation (GO:0045666) | 3.16E-05 | 0.0023258 | -1.150222 | 11.919359 |
| negative regulation of nucleic acid-templated transcription (GO:1903507) | 3.91E-05 | 0.002789 | -1.216475 | 12.347567 |
| regulation of ERK1 and ERK2 cascade (GO:0070372) | 5.31E-05 | 0.0036809 | -1.045779 | 10.293548 |
| axon guidance (GO:0007411) | 5.53E-05 | 0.0037255 | -1.536175 | 15.057458 |
| negative regulation of cellular process (GO:0048523) | 6.10E-05 | 0.0039928 | -1.352424 | 13.124541 |
| positive regulation of protein modification process (GO:0031401) | 7.33E-05 | 0.0046656 | -1.050003 | 9.9974292 |
| cellular protein modification process (GO:0006464) | 8.16E-05 | 0.0050585 | -1.694281 | 15.949661 |
| type I interferon signaling pathway (GO:0060337) | 9.07E-05 | 0.0052929 | -2.257752 | 21.016126 |
| cellular response to type I interferon (GO:0071357) | 9.07E-05 | 0.0052929 | -1.364194 | 12.698499 |
| neuron differentiation (GO:0030182) | 9.21E-05 | 0.0052929 | -1.715623 | 15.942492 |
| positive regulation of intracellular signal transduction (GO:1902533) | 0.000110364 | 0.0061909 | -1.545507 | 14.082244 |
| positive regulation of cellular process (GO:0048522) | 0.000115337 | 0.0063194 | -1.316434 | 11.936967 |
| positive regulation of phospholipase C activity (GO:0010863) | 0.000126328 | 0.0067643 | -1.598936 | 14.353054 |
| neural tube development (GO:0021915) | 0.00013299 | 0.0069627 | -2.347221 | 20.949507 |
| regulation of cortisol biosynthetic process (GO:2000064) | 0.000137933 | 0.0070646 | -2.920383 | 25.958524 |
| regulation of angiogenesis (GO:0045765) | 0.000152029 | 0.0074661 | -1.716893 | 15.093969 |
| positive regulation of apoptotic process (GO:0043065) | 0.000152111 | 0.0074661 | -1.630244 | 14.331305 |
| positive regulation of macromolecule metabolic process (GO:0010604) | 0.000171438 | 0.008243 | -1.779585 | 15.4313 |
| positive regulation of neuron projection development (GO:0010976) | 0.000185403 | 0.0087362 | -1.19602 | 10.277377 |
| glycosylceramide metabolic process (GO:0006677) | 0.000218061 | 0.0100736 | -2.58894 | 21.826671 |
| positive regulation of osteoblast differentiation (GO:0045669) | 0.000262434 | 0.0115804 | -1.921216 | 15.841402 |
| response to interferon-beta (GO:0035456) | 0.000260598 | 0.0115804 | -2.090739 | 17.253888 |
| negative regulation of cellular macromolecule biosynthetic process (GO:2000113) | 0.000265424 | 0.0115804 | -1.028475 | 8.4686523 |
| positive regulation of MAPK cascade (GO:0043410) | 0.000274542 | 0.0117604 | -1.075661 | 8.8208598 |
| positive regulation of programmed cell death (GO:0043068) | 0.000299502 | 0.0126005 | -1.249832 | 10.140369 |
| regulation of endothelial cell proliferation (GO:0001936) | 0.000305249 | 0.0126169 | -1.308441 | 10.591023 |
| negative regulation of MAPK cascade (GO:0043409) | 0.000329584 | 0.013161 | -1.680124 | 13.470694 |
| cellular response to organic substance (GO:0071310) | 0.000326026 | 0.013161 | -1.098851 | 8.8221604 |
| mRNA splicing, via spliceosome (GO:0000398) | 0.000347152 | 0.0136315 | -1.675372 | 13.345586 |
| negative regulation of cell death (GO:0060548) | 0.000355451 | 0.0137286 | -1.419824 | 11.276413 |
| modulation of chemical synaptic transmission (GO:0050804) | 0.000382919 | 0.0145509 | -1.364281 | 10.733735 |
| positive regulation of transferase activity (GO:0051347) | 0.000405025 | 0.0151467 | -1.171776 | 9.1534009 |
| mitral valve morphogenesis (GO:0003183) | 0.000456206 | 0.0165357 | -2.661374 | 20.472793 |
| cellular response to growth factor stimulus (GO:0071363) | 0.000450072 | 0.0165357 | -1.377237 | 10.613131 |
| negative regulation of ERK1 and ERK2 cascade (GO:0070373) | 0.0004885 | 0.017224 | -1.429541 | 10.899065 |
| nuclear-transcribed mRNA catabolic process, nonsense-mediated decay (GO:0000184) | 0.000489815 | 0.017224 | -1.918687 | 14.623241 |
| negative regulation of proteolysis (GO:0045861) | 0.000547282 | 0.0189617 | -1.783207 | 13.392855 |
| negative regulation of neuron differentiation (GO:0045665) | 0.000618087 | 0.0194706 | -2.015981 | 14.895839 |
| neuron projection fasciculation (GO:0106030) | 0.000619819 | 0.0194706 | -2.482845 | 18.338497 |
| glycerolipid metabolic process (GO:0046486) | 0.000619819 | 0.0194706 | -2.161796 | 15.967202 |
| regulation of mRNA 3'-end processing (GO:0031440) | 0.000619819 | 0.0194706 | -1.63704 | 12.091312 |
| cellular response to interferon-gamma (GO:0071346) | 0.000617629 | 0.0194706 | -1.885492 | 13.933076 |
| positive regulation of peptidyl-tyrosine phosphorylation (GO:0050731) | 0.000617629 | 0.0194706 | -1.207536 | 8.9232336 |
| peptidyl-serine phosphorylation (GO:0018105) | 0.000610551 | 0.0194706 | -1.396944 | 10.338989 |
| positive regulation of protein localization to plasma membrane (GO:1903078) | 0.000680596 | 0.0210985 | -1.487605 | 10.848418 |
| enzyme linked receptor protein signaling pathway (GO:0007167) | 0.000771113 | 0.023594 | -1.671667 | 11.981967 |
| regulation of protein localization to plasma membrane (GO:1903076) | 0.000786721 | 0.0236223 | -1.735339 | 12.40357 |
| Fc-epsilon receptor signaling pathway (GO:0038095) | 0.000792089 | 0.0236223 | -1.153308 | 8.2355816 |
| calcineurin-NFAT signaling cascade (GO:0033173) | 0.000816599 | 0.0237305 | -2.241923 | 15.940888 |
| phosphate-containing compound metabolic process (GO:0006796) | 0.000825933 | 0.0237305 | -1.293697 | 9.1839531 |
| Fc receptor signaling pathway (GO:0038093) | 0.000825933 | 0.0237305 | -1.270473 | 9.0190813 |
| positive regulation of protein localization to cell periphery (GO:1904377) | 0.000924249 | 0.025618 | -1.413576 | 9.8759863 |
| regulation of cellular component movement (GO:0051270) | 0.000924249 | 0.025618 | -1.385884 | 9.6825178 |
| regulation of osteoblast differentiation (GO:0045667) | 0.000917487 | 0.025618 | -1.413563 | 9.8862782 |
| positive regulation of cell migration (GO:0030335) | 0.000987112 | 0.0270423 | -1.791626 | 12.399353 |
| regulation of cell communication (GO:0010646) | 0.001064371 | 0.0272572 | -1.58512 | 10.850735 |
| mRNA metabolic process (GO:0016071) | 0.001039733 | 0.0272572 | -1.589492 | 10.917893 |
| calcineurin-mediated signaling (GO:0097720) | 0.001048966 | 0.0272572 | -2.267201 | 15.552888 |
| axonal fasciculation (GO:0007413) | 0.001048966 | 0.0272572 | -2.175593 | 14.924461 |
| axonogenesis (GO:0007409) | 0.00106156 | 0.0272572 | -1.582001 | 10.833566 |
| protein deubiquitination (GO:0016579) | 0.001022634 | 0.0272572 | -1.672854 | 11.518224 |
| negative regulation of cell cycle (GO:0045786) | 0.001144258 | 0.0286361 | -1.590737 | 10.774063 |
| regulation of fat cell differentiation (GO:0045598) | 0.001144258 | 0.0286361 | -1.213245 | 8.2173044 |
| protein modification by small protein removal (GO:0070646) | 0.001166838 | 0.0286361 | -1.157307 | 7.8158244 |
| regulation of protein phosphorylation (GO:0001932) | 0.001166838 | 0.0286361 | -1.096817 | 7.4073086 |
| positive regulation of cell differentiation (GO:0045597) | 0.001283205 | 0.0311673 | -1.150224 | 7.6586437 |
| modulation by virus of host process (GO:0019054) | 0.001319199 | 0.0317146 | -2.503353 | 16.599058 |
| peptidyl-tyrosine modification (GO:0018212) | 0.001466786 | 0.0345917 | -1.741612 | 11.363464 |
| regulation of intracellular signal transduction (GO:1902531) | 0.001468236 | 0.0345917 | -1.812334 | 11.82311 |
| activation of protein kinase activity (GO:0032147) | 0.001506739 | 0.0351473 | -1.183344 | 7.6891395 |
| positive regulation of ossification (GO:0045778) | 0.001598547 | 0.0369233 | -1.420178 | 9.1440426 |
| regulation of monocyte differentiation (GO:0045655) | 0.001629439 | 0.0372714 | -2.077219 | 13.334745 |
| positive regulation of ERK1 and ERK2 cascade (GO:0070374) | 0.001669008 | 0.0374494 | -1.555571 | 9.9486963 |
| RNA splicing, via transesterification reactions with bulged adenosine as nucleophile (GO:0000377) | 0.001666731 | 0.0374494 | -1.183003 | 7.5675433 |
| positive regulation of protein import into nucleus (GO:0042307) | 0.001738799 | 0.0382861 | -1.420832 | 9.0287642 |
| RNA splicing (GO:0008380) | 0.001733872 | 0.0382861 | -1.742387 | 11.077046 |
| positive regulation of epithelial cell proliferation (GO:0050679) | 0.001829015 | 0.0398996 | -1.24321 | 7.8371665 |
| regulation of erythrocyte differentiation (GO:0045646) | 0.001866996 | 0.0399877 | -1.335535 | 8.3917319 |
| peptidyl-serine modification (GO:0018209) | 0.001856184 | 0.0399877 | -1.355628 | 8.5258612 |
| regulation of phosphatidylinositol 3-kinase signaling (GO:0014066) | 0.001960108 | 0.0416037 | -1.227233 | 7.6515005 |
| atrioventricular valve morphogenesis (GO:0003181) | 0.001981694 | 0.0416864 | -2.143003 | 13.33763 |
| positive regulation of protein kinase activity (GO:0045860) | 0.002009559 | 0.0418984 | -1.25776 | 7.8104914 |
| nuclear-transcribed mRNA catabolic process (GO:0000956) | 0.002172884 | 0.0449063 | -1.158843 | 7.1056779 |
| chordate embryonic development (GO:0043009) | 0.002213728 | 0.04508 | -1.376178 | 8.4126809 |
| positive regulation of transmembrane receptor protein serine/threonine kinase signaling pathway (GO:0090100) | 0.00221956 | 0.04508 | -1.118109 | 6.8321462 |
| mRNA processing (GO:0006397) | 0.002294127 | 0.0461963 | -1.357703 | 8.2513063 |
| regulation of interferon-beta production (GO:0032648) | 0.002329469 | 0.0465104 | -1.929102 | 11.694435 |
| chloride transmembrane transport (GO:1902476) | 0.002391179 | 0.0469468 | -1.644332 | 9.9251372 |
| positive regulation of cardiac muscle tissue growth (GO:0055023) | 0.002377845 | 0.0469468 | -2.209359 | 13.347974 |
| neuron development (GO:0048666) | 0.002451949 | 0.0477421 | -1.080019 | 6.4918586 |
| regulation of neuron differentiation (GO:0045664) | 0.002504092 | 0.0479646 | -1.164596 | 6.9757337 |
| positive regulation of kinase activity (GO:0033674) | 0.002487883 | 0.0479646 | -1.762749 | 10.570012 |
| **GO Cellular Component** | | | | |
| main axon (GO:0044304) | 0.000198867 | 0.0209612 | -1.662059 | 14.165517 |
| tertiary granule (GO:0070820) | 0.00035229 | 0.0209612 | -1.771404 | 14.084531 |
| chromatin (GO:0000785) | 0.000349442 | 0.0209612 | -1.670321 | 13.294376 |
| tertiary granule membrane (GO:0070821) | 0.000187723 | 0.0209612 | -1.434626 | 12.309869 |
| nuclear chromatin (GO:0000790) | 0.000893574 | 0.0425341 | -1.953548 | 13.714453 |
| nuclear body (GO:0016604) | 0.001139545 | 0.045202 | -1.387424 | 9.4027438 |
| nuclear chromosome part (GO:0044454) | 0.001874927 | 0.0637475 | -1.194169 | 7.4984104 |
| caveola (GO:0005901) | 0.002213728 | 0.0658584 | -1.398846 | 8.5512512 |
| GABA-A receptor complex (GO:1902711) | 0.003846652 | 0.0760471 | -2.096713 | 11.658882 |
| ionotropic glutamate receptor complex (GO:0008328) | 0.003817978 | 0.0760471 | -1.829938 | 10.189158 |
| PML body (GO:0016605) | 0.003436712 | 0.0760471 | -1.287541 | 7.3045289 |
| ficolin-1-rich granule (GO:0101002) | 0.003154958 | 0.0760471 | -1.223992 | 7.0486998 |
| chromosome, telomeric region (GO:0000781) | 0.004153833 | 0.0760471 | -1.161574 | 6.3697514 |
| focal adhesion (GO:0005925) | 0.005443522 | 0.0925399 | -1.037312 | 5.4078472 |
| nuclear transcription factor complex (GO:0044798) | 0.006086362 | 0.0965703 | -2.190712 | 11.176365 |
| HFE-transferrin receptor complex (GO:1990712) | 0.008632761 | 0.1121688 | -3.643963 | 17.316808 |
| nuclear chromosome, telomeric region (GO:0000784) | 0.008070113 | 0.1121688 | -1.290641 | 6.2203574 |
| endocytic vesicle (GO:0030139) | 0.008427897 | 0.1121688 | -1.275064 | 6.089971 |
| nuclear periphery (GO:0034399) | 0.008954651 | 0.1121688 | -1.247226 | 5.8813963 |
| RNA polymerase II transcription factor complex (GO:0090575) | 0.010207298 | 0.1128234 | -1.837355 | 8.4236339 |
| early endosome (GO:0005769) | 0.010378501 | 0.1128234 | -1.549263 | 7.0770644 |
| clathrin-coated vesicle membrane (GO:0030665) | 0.010429055 | 0.1128234 | -1.374217 | 6.2707707 |
| phagocytic vesicle (GO:0045335) | 0.0114994 | 0.1189938 | -1.317409 | 5.8828363 |
| integral component of plasma membrane (GO:0005887) | 0.012347981 | 0.1224508 | -1.315023 | 5.7785548 |
| CD40 receptor complex (GO:0035631) | 0.015330849 | 0.1303122 | -2.540139 | 10.612418 |
| beta-catenin-TCF complex (GO:1990907) | 0.015330849 | 0.1303122 | -2.186783 | 9.1361367 |
| ficolin-1-rich granule lumen (GO:1904813) | 0.015245928 | 0.1303122 | -1.814006 | 7.5887892 |
| intercalated disc (GO:0014704) | 0.014523032 | 0.1303122 | -1.558012 | 6.5935361 |
| pericentric heterochromatin (GO:0005721) | 0.017927554 | 0.1333362 | -2.527961 | 10.165983 |
| methylosome (GO:0034709) | 0.017927554 | 0.1333362 | -2.420506 | 9.7338627 |
| neurotransmitter receptor complex (GO:0098878) | 0.017927554 | 0.1333362 | -2.382636 | 9.581572 |
| node of Ranvier (GO:0033268) | 0.017927554 | 0.1333362 | -2.355216 | 9.4713047 |
| cytoplasmic vesicle lumen (GO:0060205) | 0.018815242 | 0.1356978 | -1.235128 | 4.9072707 |
| AP-2 adaptor complex (GO:0030122) | 0.020695614 | 0.136821 | -2.311167 | 8.9623227 |
| clathrin coat of endocytic vesicle (GO:0030128) | 0.020695614 | 0.136821 | -2.306465 | 8.9440868 |
| perinuclear region of cytoplasm (GO:0048471) | 0.020420295 | 0.136821 | -1.274315 | 4.9586475 |
| cation channel complex (GO:0034703) | 0.02177193 | 0.1400465 | -1.104423 | 4.2267734 |
| nuclear speck (GO:0016607) | 0.022639572 | 0.1417952 | -1.022949 | 3.8749869 |
| dendrite (GO:0030425) | 0.024116642 | 0.1430943 | -1.407158 | 5.2414575 |
| phagocytic vesicle membrane (GO:0030670) | 0.024650699 | 0.1430943 | -1.339494 | 4.960079 |
| clathrin-coated vesicle (GO:0030136) | 0.023716474 | 0.1430943 | -1.284846 | 4.8073618 |
| cytoplasmic stress granule (GO:0010494) | 0.029899416 | 0.1581347 | -1.636031 | 5.7423324 |
| heterochromatin (GO:0000792) | 0.029899416 | 0.1581347 | -1.355039 | 4.7560743 |
| anchored component of plasma membrane (GO:0046658) | 0.028089464 | 0.1581347 | -1.283231 | 4.5841628 |
| coated vesicle (GO:0030135) | 0.028858802 | 0.1581347 | -1.144696 | 4.0583378 |
| early endosome membrane (GO:0031901) | 0.031491008 | 0.1626564 | -1.228506 | 4.2482387 |
| spindle (GO:0005819) | 0.032121219 | 0.1626564 | -0.883452 | 3.0375198 |
| clathrin adaptor complex (GO:0030131) | 0.033360556 | 0.1630767 | -2.046514 | 6.9589288 |
| COPII-coated ER to Golgi transport vesicle (GO:0030134) | 0.034259818 | 0.1630767 | -1.26991 | 4.2843996 |
| secretory granule lumen (GO:0034774) | 0.033928764 | 0.1630767 | -0.932265 | 3.1543117 |
| nuclear euchromatin (GO:0005719) | 0.036896064 | 0.1656842 | -1.869812 | 6.1697248 |
| anchored component of external side of plasma membrane (GO:0031362) | 0.036896064 | 0.1656842 | -1.690433 | 5.5778376 |
| AMPA glutamate receptor complex (GO:0032281) | 0.036896064 | 0.1656842 | -1.615872 | 5.3318128 |
| ribonucleoprotein granule (GO:0035770) | 0.041782926 | 0.1841544 | -1.434458 | 4.5547867 |
| nuclear heterochromatin (GO:0005720) | 0.04437174 | 0.1920086 | -1.821887 | 5.6754543 |
| specific granule (GO:0042581) | 0.046332396 | 0.1969127 | -1.025559 | 3.1504279 |
| U1 snRNP (GO:0005685) | 0.048301469 | 0.1982026 | -1.913942 | 5.7998064 |
| euchromatin (GO:0000791) | 0.048301469 | 0.1982026 | -1.816398 | 5.5042176 |
| intrinsic component of external side of plasma membrane (GO:0031233) | 0.052352386 | 0.2076645 | -1.439905 | 4.2473708 |
| lysosomal lumen (GO:0043202) | 0.051945635 | 0.2076645 | -1.151645 | 3.4060571 |
| cortical actin cytoskeleton (GO:0030864) | 0.053703021 | 0.2095298 | -1.122361 | 3.2821033 |
| microtubule (GO:0005874) | 0.055161522 | 0.2117491 | -0.727076 | 2.1066965 |
| specific granule membrane (GO:0035579) | 0.061352853 | 0.218061 | -1.088314 | 3.0376086 |
| tertiary granule lumen (GO:1904724) | 0.061386911 | 0.218061 | -1.048797 | 2.9267301 |
| ER to Golgi transport vesicle membrane (GO:0012507) | 0.058771257 | 0.218061 | -1.002466 | 2.8410926 |
| cytoplasmic ribonucleoprotein granule (GO:0036464) | 0.0587591 | 0.218061 | -0.874307 | 2.4780565 |
| cytoplasmic vesicle (GO:0031410) | 0.060991232 | 0.218061 | -0.742788 | 2.0775967 |
| Golgi-associated vesicle (GO:0005798) | 0.069552084 | 0.2331464 | -0.956536 | 2.5498172 |
| microtubule organizing center (GO:0005815) | 0.06860885 | 0.2331464 | -0.916418 | 2.4553899 |
| endoplasmic reticulum-Golgi intermediate compartment (GO:0005793) | 0.069482464 | 0.2331464 | -0.751867 | 2.004989 |
| polymeric cytoskeletal fiber (GO:0099513) | 0.068467783 | 0.2331464 | -0.676086 | 1.8128524 |
| U12-type spliceosomal complex (GO:0005689) | 0.074257828 | 0.2345463 | -1.418013 | 3.6871343 |
| polysomal ribosome (GO:0042788) | 0.078937389 | 0.2345463 | -1.368622 | 3.4750679 |
| brush border membrane (GO:0031526) | 0.078937389 | 0.2345463 | -1.159751 | 2.9447249 |
| nuclear matrix (GO:0016363) | 0.072377558 | 0.2345463 | -0.974617 | 2.559208 |
| chromosome (GO:0005694) | 0.073744943 | 0.2345463 | -0.867914 | 2.2627756 |
| ficolin-1-rich granule membrane (GO:0101003) | 0.078179767 | 0.2345463 | -0.886935 | 2.2605703 |
| filopodium (GO:0030175) | 0.075253702 | 0.2345463 | -0.82136 | 2.1247686 |
| centrosome (GO:0005813) | 0.071687499 | 0.2345463 | -0.672323 | 1.7718672 |
| axon (GO:0030424) | 0.079824566 | 0.2345463 | -0.65749 | 1.6620844 |
| lytic vacuole (GO:0000323) | 0.077622083 | 0.2345463 | -0.624542 | 1.5962698 |
| lysosome (GO:0005764) | 0.081701452 | 0.2371335 | -0.529326 | 1.3257936 |
| sarcoplasmic reticulum (GO:0016529) | 0.083706419 | 0.2400256 | -1.321519 | 3.2779473 |
| sarcoplasm (GO:0016528) | 0.093496697 | 0.2649073 | -1.34549 | 3.1885819 |
| platelet alpha granule lumen (GO:0031093) | 0.096740904 | 0.2708745 | -0.946492 | 2.2107404 |
| nucleolar part (GO:0044452) | 0.103670789 | 0.2869029 | -0.622388 | 1.4106644 |
| axolemma (GO:0030673) | 0.107415665 | 0.2905105 | -2.040317 | 4.5520487 |
| box C/D snoRNP complex (GO:0031428) | 0.107415665 | 0.2905105 | -1.411376 | 3.1488504 |
| extrinsic component of external side of plasma membrane (GO:0031232) | 0.121791305 | 0.3051193 | -2.673181 | 5.6282394 |
| NURF complex (GO:0016589) | 0.121791305 | 0.3051193 | -2.420369 | 5.0959579 |
| cyclin-dependent protein kinase activating kinase holoenzyme complex (GO:0019907) | 0.121791305 | 0.3051193 | -2.325636 | 4.8965022 |
| actin cortical patch (GO:0030479) | 0.121791305 | 0.3051193 | -2.160742 | 4.5493254 |
| endocytic patch (GO:0061645) | 0.121791305 | 0.3051193 | -1.458864 | 3.0715607 |
| U7 snRNP (GO:0005683) | 0.121791305 | 0.3051193 | -1.444872 | 3.0421002 |
| vacuolar lumen (GO:0005775) | 0.121313602 | 0.3051193 | -0.665082 | 1.4029076 |
| commitment complex (GO:0000243) | 0.135936123 | 0.3081219 | -2.5218 | 5.0324298 |
| clathrin-sculpted gamma-aminobutyric acid transport vesicle membrane (GO:0061202) | 0.135936123 | 0.3081219 | -2.413986 | 4.8172795 |
| death-inducing signaling complex (GO:0031264) | 0.135936123 | 0.3081219 | -2.40553 | 4.8004042 |
| spectrin-associated cytoskeleton (GO:0014731) | 0.135936123 | 0.3081219 | -2.328328 | 4.646341 |
| chromatoid body (GO:0033391) | 0.135936123 | 0.3081219 | -2.151507 | 4.2934829 |
| COPI-coated vesicle (GO:0030137) | 0.135936123 | 0.3081219 | -1.805066 | 3.6021353 |
| clathrin-sculpted gamma-aminobutyric acid transport vesicle (GO:0061200) | 0.135936123 | 0.3081219 | -1.617927 | 3.2286871 |
| membrane raft (GO:0045121) | 0.128732343 | 0.3081219 | -0.673998 | 1.3817084 |
| ribosome (GO:0005840) | 0.127516053 | 0.3081219 | -0.571794 | 1.1776165 |
| nucleoplasm part (GO:0044451) | 0.124633511 | 0.3081219 | -0.400807 | 0.8346322 |
| integral component of Golgi membrane (GO:0030173) | 0.140979536 | 0.316539 | -0.800652 | 1.5685901 |
| cytosolic ribosome (GO:0022626) | 0.143101385 | 0.3183003 | -0.621462 | 1.2082484 |
| aggresome (GO:0016235) | 0.146533542 | 0.3229165 | -0.992341 | 1.9057927 |
| junctional sarcoplasmic reticulum membrane (GO:0014701) | 0.149853814 | 0.3242292 | -2.258864 | 4.2875377 |
| juxtaparanode region of axon (GO:0044224) | 0.149853814 | 0.3242292 | -1.257755 | 2.387338 |
| microvillus (GO:0005902) | 0.152131788 | 0.3261925 | -0.819372 | 1.5428844 |
| sodium channel complex (GO:0034706) | 0.163548015 | 0.3340406 | -1.785934 | 3.2336995 |
| CatSper complex (GO:0036128) | 0.163548015 | 0.3340406 | -1.206228 | 2.1840552 |
| filopodium tip (GO:0032433) | 0.163548015 | 0.3340406 | -1.002441 | 1.815068 |
| mitotic spindle (GO:0072686) | 0.157378962 | 0.3340406 | -0.525761 | 0.9721845 |
| trans-Golgi network membrane (GO:0032588) | 0.161254124 | 0.3340406 | -0.29095 | 0.5309183 |
| fibrillar center (GO:0001650) | 0.164213236 | 0.3340406 | -0.266513 | 0.4814787 |
| small nucleolar ribonucleoprotein complex (GO:0005732) | 0.190280198 | 0.347739 | -1.786079 | 2.9635657 |
| U4 snRNP (GO:0005687) | 0.190280198 | 0.347739 | -1.568876 | 2.6031698 |
| nuclear exosome (RNase complex) (GO:0000176) | 0.190280198 | 0.347739 | -1.532759 | 2.5432417 |
| ISWI-type complex (GO:0031010) | 0.177022302 | 0.347739 | -1.44181 | 2.4964641 |
| npBAF complex (GO:0071564) | 0.177022302 | 0.347739 | -1.155606 | 2.0009087 |
| cytosolic small ribosomal subunit (GO:0022627) | 0.192328585 | 0.347739 | -0.67768 | 1.1171895 |
| histone methyltransferase complex (GO:0035097) | 0.192328585 | 0.347739 | -0.583583 | 0.962066 |
| azurophil granule lumen (GO:0035578) | 0.181043341 | 0.347739 | -0.559546 | 0.9562747 |
| platelet alpha granule (GO:0031091) | 0.181043341 | 0.347739 | -0.473724 | 0.8096029 |
| chromosome, centromeric region (GO:0000775) | 0.192328585 | 0.347739 | -0.417204 | 0.6877817 |
| peroxisome (GO:0005777) | 0.189135934 | 0.347739 | -0.19884 | 0.3311269 |
| microbody (GO:0042579) | 0.189135934 | 0.347739 | -0.144176 | 0.2400947 |
| endoplasmic reticulum-Golgi intermediate compartment membrane (GO:0033116) | 0.180689595 | 0.347739 | 0.0131712 | -0.022536 |
| microtubule cytoskeleton (GO:0015630) | 0.177537338 | 0.347739 | 0.0462218 | -0.079898 |
| late endosome (GO:0005770) | 0.192863666 | 0.347739 | 0.0780705 | -0.128486 |
| actin cytoskeleton (GO:0015629) | 0.199462574 | 0.356933 | -0.157747 | 0.2543084 |
| Sin3 complex (GO:0016580) | 0.203325166 | 0.3607043 | -1.040535 | 1.6575186 |
| nucleolus (GO:0005730) | 0.204601196 | 0.3607043 | 0.3490765 | -0.553877 |
| cortical cytoskeleton (GO:0030863) | 0.209958339 | 0.3647451 | -0.537814 | 0.8394456 |
| nuclear chromosome (GO:0000228) | 0.209958339 | 0.3647451 | -0.172383 | 0.2690627 |
| endoplasmic reticulum subcompartment (GO:0098827) | 0.216160616 | 0.3701167 | -1.029505 | 1.5769276 |
| small ribosomal subunit (GO:0015935) | 0.215871747 | 0.3701167 | -0.598116 | 0.9169548 |
| nuclear replisome (GO:0043601) | 0.241216326 | 0.3727889 | -1.272671 | 1.8098157 |
| BLOC-1 complex (GO:0031083) | 0.241216326 | 0.3727889 | -1.20529 | 1.7139964 |
| SWI/SNF complex (GO:0016514) | 0.228789902 | 0.3727889 | -1.148492 | 1.6939696 |
| CHD-type complex (GO:0090545) | 0.241216326 | 0.3727889 | -1.094714 | 1.5567504 |
| synaptonemal structure (GO:0099086) | 0.228789902 | 0.3727889 | -1.010131 | 1.4898934 |
| keratin filament (GO:0045095) | 0.228789902 | 0.3727889 | -0.910794 | 1.3433763 |
| secretory vesicle (GO:0099503) | 0.241216326 | 0.3727889 | -0.87909 | 1.2501201 |
| nuclear cyclin-dependent protein kinase holoenzyme complex (GO:0019908) | 0.241216326 | 0.3727889 | -0.711723 | 1.0121135 |
| NuRD complex (GO:0016581) | 0.241216326 | 0.3727889 | -0.534361 | 0.7598943 |
| cytoplasmic exosome (RNase complex) (GO:0000177) | 0.228789902 | 0.3727889 | -0.407286 | 0.6007269 |
| Sin3-type complex (GO:0070822) | 0.241216326 | 0.3727889 | -0.339292 | 0.4824946 |
| exocytic vesicle (GO:0070382) | 0.241216326 | 0.3727889 | -0.248661 | 0.3536113 |
| Cul2-RING ubiquitin ligase complex (GO:0031462) | 0.228789902 | 0.3727889 | -0.17331 | 0.2556232 |
| T cell receptor complex (GO:0042101) | 0.241216326 | 0.3727889 | 0.1176979 | -0.167374 |
| mitochondrion (GO:0005739) | 0.220303636 | 0.3727889 | 0.4189623 | -0.633785 |
| BAF-type complex (GO:0090544) | 0.253443135 | 0.3769967 | -1.086879 | 1.4918677 |
| platelet alpha granule membrane (GO:0031092) | 0.253443135 | 0.3769967 | -1.042278 | 1.4306471 |
| endoplasmic reticulum tubular network (GO:0071782) | 0.253443135 | 0.3769967 | -0.951503 | 1.3060481 |
| mRNA cleavage and polyadenylation specificity factor complex (GO:0005847) | 0.253443135 | 0.3769967 | -0.739108 | 1.0145107 |
| spliceosomal snRNP complex (GO:0097525) | 0.251597257 | 0.3769967 | -0.106134 | 0.146457 |
| spindle pole (GO:0000922) | 0.252278601 | 0.3769967 | 0.2026919 | -0.279152 |
| cytosolic part (GO:0044445) | 0.257644599 | 0.3808659 | 0.139181 | -0.188754 |
| ESC/E(Z) complex (GO:0035098) | 0.265473528 | 0.3829255 | -1.193301 | 1.5826042 |
| MHC protein complex (GO:0042611) | 0.265473528 | 0.3829255 | -0.600396 | 0.796269 |
| endocytic vesicle lumen (GO:0071682) | 0.265473528 | 0.3829255 | -0.582146 | 0.7720655 |
| DNA-directed RNA polymerase III complex (GO:0005666) | 0.265473528 | 0.3829255 | -0.42347 | 0.5616234 |
| vacuole (GO:0005773) | 0.269535169 | 0.384128 | 0.0222568 | -0.02918 |
| specific granule lumen (GO:0035580) | 0.269535169 | 0.384128 | 0.5966142 | -0.782195 |
| polysome (GO:0005844) | 0.275514413 | 0.3903121 | -0.145441 | 0.1874898 |
| small nuclear ribonucleoprotein complex (GO:0030532) | 0.27731065 | 0.3905322 | -0.905871 | 1.1618855 |
| autophagosome (GO:0005776) | 0.281491068 | 0.3912387 | -0.267719 | 0.339375 |
| spliceosomal complex (GO:0005681) | 0.282743943 | 0.3912387 | -0.1216 | 0.1536071 |
| lipid droplet (GO:0005811) | 0.281491068 | 0.3912387 | -0.053166 | 0.0673963 |
| endolysosome (GO:0036019) | 0.288957595 | 0.3952408 | -1.085317 | 1.3473946 |
| dendrite membrane (GO:0032590) | 0.288957595 | 0.3952408 | 0.4901523 | -0.608512 |
| pigment granule (GO:0048770) | 0.300417411 | 0.4039511 | -0.85638 | 1.0298679 |
| U2 snRNP (GO:0005686) | 0.300417411 | 0.4039511 | -0.631218 | 0.7590919 |
| melanosome (GO:0042470) | 0.300417411 | 0.4039511 | -0.101228 | 0.1217348 |
| recycling endosome (GO:0055037) | 0.304699368 | 0.407407 | 0.098123 | -0.116612 |
| telomerase holoenzyme complex (GO:0005697) | 0.311693094 | 0.4121275 | -0.60732 | 0.7079746 |
| cytosolic large ribosomal subunit (GO:0022625) | 0.311285951 | 0.4121275 | 0.8460669 | -0.987397 |
| actin-based cell projection (GO:0098858) | 0.31721798 | 0.4171154 | 0.827975 | -0.950653 |
| U5 snRNP (GO:0005682) | 0.322787594 | 0.4198003 | -1.067228 | 1.2067797 |
| prespliceosome (GO:0071010) | 0.322787594 | 0.4198003 | -0.855599 | 0.9674773 |
| endosome lumen (GO:0031904) | 0.333703813 | 0.4247139 | -0.801532 | 0.8796822 |
| calcium channel complex (GO:0034704) | 0.333703813 | 0.4247139 | -0.475759 | 0.5221464 |
| large ribosomal subunit (GO:0015934) | 0.329045282 | 0.4247139 | 0.0814352 | -0.09052 |
| cytoskeleton (GO:0005856) | 0.330808879 | 0.4247139 | 1.0455386 | -1.15659 |
| serine/threonine protein kinase complex (GO:1902554) | 0.344444607 | 0.4337451 | -0.549157 | 0.5853036 |
| nuclear inner membrane (GO:0005637) | 0.344444607 | 0.4337451 | -0.101489 | 0.1081692 |
| preribosome (GO:0030684) | 0.346677638 | 0.4342594 | -0.053171 | 0.0563275 |
| chromosomal region (GO:0098687) | 0.352521947 | 0.4355312 | 0.0236487 | -0.024657 |
| preribosome, large subunit precursor (GO:0030687) | 0.355012788 | 0.4355312 | 0.0913111 | -0.094562 |
| condensed chromosome, centromeric region (GO:0000779) | 0.355012788 | 0.4355312 | 0.1615652 | -0.167317 |
| trans-Golgi network (GO:0005802) | 0.35151917 | 0.4355312 | 0.4239717 | -0.443259 |
| synaptonemal complex (GO:0000795) | 0.375642324 | 0.4538217 | -0.356936 | 0.3494822 |
| catenin complex (GO:0016342) | 0.375642324 | 0.4538217 | -0.305672 | 0.299289 |
| rough endoplasmic reticulum (GO:0005791) | 0.375642324 | 0.4538217 | 0.1270946 | -0.124441 |
| mitochondrial matrix (GO:0005759) | 0.379535018 | 0.4562088 | 0.2393336 | -0.231868 |
| integral component of lumenal side of endoplasmic reticulum membrane (GO:0071556) | 0.38570908 | 0.4589938 | 0.3240823 | -0.308744 |
| U2-type catalytic step 2 spliceosome (GO:0071007) | 0.38570908 | 0.4589938 | 0.4667208 | -0.444632 |
| spindle midzone (GO:0051233) | 0.395614021 | 0.4684385 | -0.236154 | 0.2189895 |
| endosomal part (GO:0044440) | 0.405359741 | 0.4752494 | -0.258003 | 0.2329719 |
| spliceosomal tri-snRNP complex (GO:0097526) | 0.405359741 | 0.4752494 | 0.2821543 | -0.25478 |
| precatalytic spliceosome (GO:0071011) | 0.424383684 | 0.4903074 | -0.382032 | 0.3274462 |
| clathrin-coated endocytic vesicle membrane (GO:0030669) | 0.424383684 | 0.4903074 | 1.1605583 | -0.994735 |
| Golgi membrane (GO:0000139) | 0.42133872 | 0.4903074 | 1.673223 | -1.446197 |
| site of double-strand break (GO:0035861) | 0.433666888 | 0.496215 | 0.4050622 | -0.338421 |
| Cajal body (GO:0015030) | 0.433666888 | 0.496215 | 0.4080246 | -0.340896 |
| small-subunit processome (GO:0032040) | 0.442800835 | 0.5018409 | 0.4133296 | -0.336713 |
| endoplasmic reticulum lumen (GO:0005788) | 0.442752716 | 0.5018409 | 1.1944874 | -0.973201 |
| site of DNA damage (GO:0090734) | 0.460630492 | 0.5146951 | -0.35207 | 0.2729102 |
| azurophil granule (GO:0042582) | 0.456546348 | 0.5146951 | 0.1755436 | -0.137638 |
| centriole (GO:0005814) | 0.459201033 | 0.5146951 | 0.3438227 | -0.267586 |
| condensed chromosome (GO:0000793) | 0.486314133 | 0.540854 | 1.0270182 | -0.740378 |
| nuclear ubiquitin ligase complex (GO:0000152) | 0.494601464 | 0.5475123 | -0.123058 | 0.0866329 |
| microbody lumen (GO:0031907) | 0.502755504 | 0.5514093 | -0.34027 | 0.2339869 |
| peroxisomal matrix (GO:0005782) | 0.502755504 | 0.5514093 | 1.0127247 | -0.696401 |
| lytic vacuole membrane (GO:0098852) | 0.522024752 | 0.5699169 | 0.3179729 | -0.206695 |
| intermediate filament (GO:0005882) | 0.534080984 | 0.5804168 | -0.153448 | 0.0962439 |
| recycling endosome membrane (GO:0055038) | 0.54159995 | 0.5859127 | -0.063738 | 0.0390859 |
| late endosome membrane (GO:0031902) | 0.548997946 | 0.5912286 | 1.2047366 | -0.722433 |
| clathrin-coated endocytic vesicle (GO:0045334) | 0.563438759 | 0.595993 | 0.0057791 | -0.003315 |
| mitochondrial outer membrane (GO:0005741) | 0.559725599 | 0.595993 | 0.6316786 | -0.366569 |
| spindle microtubule (GO:0005876) | 0.556276912 | 0.595993 | 0.9772802 | -0.573164 |
| kinesin complex (GO:0005871) | 0.563438759 | 0.595993 | 1.0904415 | -0.625583 |
| nuclear DNA-directed RNA polymerase complex (GO:0055029) | 0.577418575 | 0.608078 | 1.3857257 | -0.761024 |
| SCF ubiquitin ligase complex (GO:0019005) | 0.590952068 | 0.6195885 | 0.2458085 | -0.1293 |
| actin filament (GO:0005884) | 0.5975559 | 0.6237645 | 0.0099966 | -0.005147 |
| microtubule organizing center part (GO:0044450) | 0.609075177 | 0.6330126 | 0.5019591 | -0.248878 |
| U2-type spliceosomal complex (GO:0005684) | 0.629014428 | 0.6508932 | 0.7778603 | -0.360617 |
| Golgi subcompartment (GO:0098791) | 0.65684948 | 0.676754 | 0.7940042 | -0.33372 |
| intermediate filament cytoskeleton (GO:0045111) | 0.689859305 | 0.7067487 | 0.2986312 | -0.110872 |
| bicellular tight junction (GO:0005923) | 0.694870611 | 0.7067487 | 0.4946846 | -0.18008 |
| lysosomal membrane (GO:0005765) | 0.69463862 | 0.7067487 | 1.0579792 | -0.385489 |
| peroxisomal part (GO:0044439) | 0.709425337 | 0.7184818 | 1.4307987 | -0.491193 |
| cullin-RING ubiquitin ligase complex (GO:0031461) | 0.793996273 | 0.8007251 | 0.9473951 | -0.218542 |
| integral component of endoplasmic reticulum membrane (GO:0030176) | 0.877615168 | 0.8813182 | 1.3624881 | -0.177869 |
| mitochondrial inner membrane (GO:0005743) | 0.996299434 | 0.9962994 | 1.7880803 | -0.006629 |
| **GO Molecular Function** | | | | |
| 1-phosphatidylinositol-4-phosphate 5-kinase activity (GO:0016308) | 0.000137933 | 0.0046208 | -3.907877 | 34.736102 |
| protein homodimerization activity (GO:0042803) | 4.10642E-07 | 8.018E-05 | -2.200881 | 32.36515 |
| transcription factor activity, RNA polymerase II core promoter proximal region sequence-specific binding (GO:0000982) | 3.31585E-08 | 1.555E-05 | -1.627016 | 28.020425 |
| RNA polymerase II regulatory region sequence-specific DNA binding (GO:0000977) | 5.12856E-07 | 8.018E-05 | -1.84847 | 26.771891 |
| transcriptional activator activity, RNA polymerase II core promoter proximal region sequence-specific binding (GO:0001077) | 2.94945E-05 | 0.0017291 | -1.894709 | 19.764297 |
| calcium-dependent protein serine/threonine phosphatase activity (GO:0004723) | 0.000218061 | 0.0063919 | -2.328185 | 19.628311 |
| core promoter proximal region sequence-specific DNA binding (GO:0000987) | 1.28989E-05 | 0.0010083 | -1.564109 | 17.609316 |
| GABA-gated chloride ion channel activity (GO:0022851) | 0.001048966 | 0.0213898 | -2.434968 | 16.703761 |
| core promoter sequence-specific DNA binding (GO:0001046) | 2.86709E-06 | 0.0003362 | -1.290415 | 16.468549 |
| extracellular ligand-gated ion channel activity (GO:0005230) | 0.001598547 | 0.0267757 | -2.514332 | 16.188933 |
| core promoter binding (GO:0001047) | 4.07428E-06 | 0.0003822 | -1.26801 | 15.737042 |
| phosphatidylinositol phosphate kinase activity (GO:0016307) | 0.001981694 | 0.0299811 | -2.440366 | 15.188355 |
| transmitter-gated ion channel activity (GO:0022824) | 0.000198867 | 0.0062179 | -1.712647 | 14.596675 |
| transcriptional activator activity, RNA polymerase II transcription regulatory region sequence-specific binding (GO:0001228) | 6.42684E-05 | 0.0028614 | -1.415945 | 13.667324 |
| cAMP-dependent protein kinase inhibitor activity (GO:0004862) | 0.0106769 | 0.0848723 | -2.982188 | 13.538158 |
| neurotrophin TRKA receptor binding (GO:0005168) | 0.005144158 | 0.052448 | -2.547104 | 13.422965 |
| protein heterodimerization activity (GO:0046982) | 0.000112249 | 0.0043871 | -1.465576 | 13.329099 |
| transmembrane receptor protein kinase activity (GO:0019199) | 6.71124E-05 | 0.0028614 | -1.369095 | 13.15583 |
| transcriptional activator activity, RNA polymerase II transcription factor binding (GO:0001190) | 0.001343196 | 0.0233318 | -1.963146 | 12.981702 |
| syntaxin-1 binding (GO:0017075) | 0.001981694 | 0.0299811 | -2.081436 | 12.95445 |
| diacylglycerol kinase activity (GO:0004143) | 0.012911807 | 0.0992727 | -2.955497 | 12.85527 |
| RNA polymerase II core promoter proximal region sequence-specific DNA binding (GO:0000978) | 2.56127E-05 | 0.0017161 | -1.171013 | 12.380439 |
| G-protein beta/gamma-subunit complex binding (GO:0031683) | 0.002819649 | 0.038394 | -2.097655 | 12.315633 |
| galactosidase activity (GO:0015925) | 0.008632761 | 0.0774647 | -2.586753 | 12.292742 |
| ligand-gated anion channel activity (GO:0099095) | 0.003308744 | 0.0413368 | -2.063137 | 11.782958 |
| ubiquitin-like protein ligase binding (GO:0044389) | 0.001149649 | 0.0219726 | -1.717078 | 11.621695 |
| cadherin binding (GO:0045296) | 0.001831994 | 0.0296278 | -1.814625 | 11.436401 |
| transcription regulatory region DNA binding (GO:0044212) | 0.00013357 | 0.0046208 | -1.28018 | 11.420332 |
| nucleotide kinase activity (GO:0019201) | 0.006513069 | 0.0649921 | -2.19929 | 11.071102 |
| transcriptional repressor activity, RNA polymerase II transcription regulatory region sequence-specific binding (GO:0001227) | 0.001171246 | 0.0219726 | -1.633684 | 11.026855 |
| GABA-A receptor activity (GO:0004890) | 0.003846652 | 0.0440019 | -1.982868 | 11.025842 |
| GABA receptor activity (GO:0016917) | 0.005074448 | 0.052448 | -2.085638 | 11.019548 |
| phospholipase C activity (GO:0004629) | 0.000995085 | 0.0212134 | -1.559685 | 10.781603 |
| endopeptidase inhibitor activity (GO:0004866) | 0.002250733 | 0.0329873 | -1.759525 | 10.726944 |
| RNA 7-methylguanosine cap binding (GO:0000340) | 0.0106769 | 0.0848723 | -2.354657 | 10.68937 |
| RNA polymerase II transcription coactivator activity (GO:0001105) | 0.003817978 | 0.0440019 | -1.897174 | 10.563528 |
| protein kinase binding (GO:0019901) | 5.93735E-05 | 0.0028614 | -1.082949 | 10.538896 |
| cyclin-dependent protein kinase activity (GO:0097472) | 0.002329469 | 0.0331067 | -1.732704 | 10.503853 |
| thioesterase binding (GO:0031996) | 0.015330849 | 0.110618 | -2.508001 | 10.478147 |
| MAP kinase phosphatase activity (GO:0033549) | 0.0106769 | 0.0848723 | -2.301719 | 10.449053 |
| neurotrophin TRK receptor binding (GO:0005167) | 0.008632761 | 0.0774647 | -2.189644 | 10.405607 |
| neurotransmitter receptor activity involved in regulation of postsynaptic membrane potential (GO:0099529) | 0.005074448 | 0.052448 | -1.902679 | 10.052878 |
| snRNA binding (GO:0017069) | 0.002865227 | 0.038394 | -1.696403 | 9.9326198 |
| protein serine/threonine kinase inhibitor activity (GO:0030291) | 0.013317266 | 0.1007387 | -2.279728 | 9.8454493 |
| BMP receptor binding (GO:0070700) | 0.017927554 | 0.1151784 | -2.438946 | 9.8080189 |
| glutamate receptor binding (GO:0035254) | 0.004434785 | 0.0495218 | -1.803573 | 9.7722582 |
| DNA binding (GO:0003677) | 0.000287015 | 0.0079182 | -1.139399 | 9.2929086 |
| amyloid-beta binding (GO:0001540) | 0.001227461 | 0.0221415 | -1.373058 | 9.2033408 |
| metalloendopeptidase inhibitor activity (GO:0008191) | 0.017927554 | 0.1151784 | -2.245597 | 9.0304819 |
| tumor necrosis factor receptor binding (GO:0005164) | 0.015330849 | 0.110618 | -2.149042 | 8.9784567 |
| calcium-transporting ATPase activity (GO:0005388) | 0.023628871 | 0.1246529 | -2.388955 | 8.9473186 |
| enhancer binding (GO:0035326) | 0.009084348 | 0.0774647 | -1.902591 | 8.9444662 |
| mitogen-activated protein kinase kinase binding (GO:0031434) | 0.000670831 | 0.0165589 | -1.217926 | 8.8993748 |
| RNA binding (GO:0003723) | 0.000947326 | 0.0211569 | -1.26895 | 8.8342613 |
| phosphatidylinositol phospholipase C activity (GO:0004435) | 0.007314135 | 0.0700067 | -1.783304 | 8.7701915 |
| MAP kinase kinase kinase activity (GO:0004709) | 0.008954651 | 0.0774647 | -1.834835 | 8.6523142 |
| transmembrane receptor protein tyrosine kinase activity (GO:0004714) | 0.003205407 | 0.0413368 | -1.491033 | 8.5628765 |
| protein kinase A catalytic subunit binding (GO:0034236) | 0.020695614 | 0.1229054 | -2.195568 | 8.5140469 |
| transcription regulatory region sequence-specific DNA binding (GO:0000976) | 0.000304754 | 0.0079405 | -1.050628 | 8.5058906 |
| RNA polymerase II core promoter sequence-specific DNA binding (GO:0000979) | 0.01005506 | 0.0842111 | -1.833998 | 8.435804 |
| transmembrane receptor protein serine/threonine kinase activity (GO:0004675) | 0.023628871 | 0.1246529 | -2.197623 | 8.2307261 |
| nuclear localization sequence binding (GO:0008139) | 0.011083744 | 0.0866379 | -1.813721 | 8.1658703 |
| ligand-gated ion channel activity (GO:0015276) | 0.005082784 | 0.052448 | -1.54149 | 8.1419886 |
| ionotropic glutamate receptor binding (GO:0035255) | 0.020695614 | 0.1229054 | -2.036992 | 7.8991167 |
| kinase binding (GO:0019900) | 0.003437389 | 0.0413368 | -1.377629 | 7.8153516 |
| protein tyrosine phosphatase activity (GO:0004725) | 0.003436712 | 0.0413368 | -1.33804 | 7.5910202 |
| guanylate kinase activity (GO:0004385) | 0.020695614 | 0.1229054 | -1.942875 | 7.5341444 |
| protein kinase activity (GO:0004672) | 0.000717459 | 0.0168244 | -1.033648 | 7.4834003 |
| cAMP-dependent protein kinase regulator activity (GO:0008603) | 0.121791305 | 0.3038304 | -3.539537 | 7.4523041 |
| insulin-like growth factor I binding (GO:0031994) | 0.020695614 | 0.1229054 | -1.911675 | 7.4131558 |
| MAP kinase activity (GO:0004707) | 0.023628871 | 0.1246529 | -1.962045 | 7.3484207 |
| hydrolase activity, hydrolyzing O-glycosyl compounds (GO:0004553) | 0.015788635 | 0.112195 | -1.765303 | 7.3232965 |
| tumor necrosis factor receptor superfamily binding (GO:0032813) | 0.009084348 | 0.0774647 | -1.543646 | 7.2569919 |
| ligand-gated channel activity (GO:0022834) | 0.014523032 | 0.1081159 | -1.700091 | 7.1948168 |
| insulin-like growth factor binding (GO:0005520) | 0.023628871 | 0.1246529 | -1.905395 | 7.1362481 |
| proton-transporting ATPase activity, rotational mechanism (GO:0046961) | 0.029967122 | 0.1428691 | -1.976387 | 6.9324837 |
| manganese ion transmembrane transporter activity (GO:0005384) | 0.107415665 | 0.3038304 | -3.029938 | 6.7599414 |
| semaphorin receptor binding (GO:0030215) | 0.036896064 | 0.1660074 | -2.031046 | 6.7017411 |
| ubiquitin protein ligase binding (GO:0031625) | 0.006722709 | 0.0656865 | -1.328839 | 6.6472031 |
| cyclin-dependent protein serine/threonine kinase activity (GO:0004693) | 0.017114367 | 0.1146663 | -1.568771 | 6.3815058 |
| poly(A) binding (GO:0008143) | 0.023628871 | 0.1246529 | -1.702499 | 6.3763453 |
| phosphoric diester hydrolase activity (GO:0008081) | 0.017114367 | 0.1146663 | -1.531544 | 6.2300715 |
| chloride channel activity (GO:0005254) | 0.01770775 | 0.1151784 | -1.529072 | 6.1678979 |
| BMP receptor activity (GO:0098821) | 0.107415665 | 0.3038304 | -2.745243 | 6.1247727 |
| histone methyltransferase binding (GO:1990226) | 0.107415665 | 0.3038304 | -2.715087 | 6.0574924 |
| ionotropic glutamate receptor activity (GO:0004970) | 0.029967122 | 0.1428691 | -1.718263 | 6.0270716 |
| ATPase activity, coupled to transmembrane movement of ions, rotational mechanism (GO:0044769) | 0.065183948 | 0.2321951 | -2.189688 | 5.9790356 |
| hydrogen-exporting ATPase activity (GO:0036442) | 0.048301469 | 0.1974728 | -1.956857 | 5.9298508 |
| poly-purine tract binding (GO:0070717) | 0.04437174 | 0.1858067 | -1.893913 | 5.8998269 |
| RNA cap binding (GO:0000339) | 0.033360556 | 0.1549119 | -1.732247 | 5.8903015 |
| transmitter-gated ion channel activity involved in regulation of postsynaptic membrane potential (GO:1904315) | 0.021454271 | 0.1229054 | -1.511791 | 5.808046 |
| vascular endothelial growth factor receptor 2 binding (GO:0043184) | 0.107415665 | 0.3038304 | -2.55984 | 5.7111282 |
| protein tyrosine kinase binding (GO:1990782) | 0.044235742 | 0.1858067 | -1.798504 | 5.6081336 |
| phosphotransferase activity, phosphate group as acceptor (GO:0016776) | 0.029899416 | 0.1428691 | -1.588751 | 5.5763849 |
| methyl-CpG binding (GO:0008327) | 0.04437174 | 0.1858067 | -1.776338 | 5.5335643 |
| kinase activity (GO:0016301) | 0.016086306 | 0.1126041 | -1.329415 | 5.4902013 |
| activin-activated receptor activity (GO:0017002) | 0.121791305 | 0.3038304 | -2.592637 | 5.4586583 |
| AP-2 adaptor complex binding (GO:0035612) | 0.121791305 | 0.3038304 | -2.583369 | 5.4391454 |
| G-protein coupled receptor binding (GO:0001664) | 0.021488795 | 0.1229054 | -1.398034 | 5.368763 |
| 7SK snRNA binding (GO:0097322) | 0.107415665 | 0.3038304 | -2.39818 | 5.3504567 |
| solute:proton antiporter activity (GO:0015299) | 0.107415665 | 0.3038304 | -2.395333 | 5.3441058 |
| SUMO transferase activity (GO:0019789) | 0.036896064 | 0.1660074 | -1.618893 | 5.3417818 |
| signal sequence binding (GO:0005048) | 0.026339839 | 0.1343906 | -1.466651 | 5.3337298 |
| glutamate receptor activity (GO:0008066) | 0.04437174 | 0.1858067 | -1.695452 | 5.2815908 |
| phosphofructokinase activity (GO:0008443) | 0.121791305 | 0.3038304 | -2.495232 | 5.253576 |
| primary amine oxidase activity (GO:0008131) | 0.107415665 | 0.3038304 | -2.351409 | 5.2461097 |
| phosphotransferase activity, alcohol group as acceptor (GO:0016773) | 0.008590112 | 0.0774647 | -1.098516 | 5.2257986 |
| ATPase activity, coupled to transmembrane movement of ions, phosphorylative mechanism (GO:0015662) | 0.052352386 | 0.2080786 | -1.770438 | 5.2223638 |
| voltage-gated ion channel activity involved in regulation of postsynaptic membrane potential (GO:1905030) | 0.056519597 | 0.2155097 | -1.785644 | 5.1304562 |
| voltage-gated sodium channel activity (GO:0005248) | 0.056519597 | 0.2155097 | -1.771573 | 5.0900265 |
| superoxide-generating NADPH oxidase activity (GO:0016175) | 0.121791305 | 0.3038304 | -2.412679 | 5.0797651 |
| phospholipase binding (GO:0043274) | 0.121791305 | 0.3038304 | -2.397591 | 5.0479991 |
| malate dehydrogenase activity (GO:0016615) | 0.107415665 | 0.3038304 | -2.245671 | 5.0102024 |
| clathrin light chain binding (GO:0032051) | 0.107415665 | 0.3038304 | -2.222781 | 4.9591346 |
| transcription coactivator activity (GO:0003713) | 0.020413835 | 0.1229054 | -1.256083 | 4.8881005 |
| MAP kinase tyrosine/serine/threonine phosphatase activity (GO:0017017) | 0.121791305 | 0.3038304 | -2.315829 | 4.8758535 |
| enhancer sequence-specific DNA binding (GO:0001158) | 0.020707114 | 0.1229054 | -1.24994 | 4.8463655 |
| dolichyl-diphosphooligosaccharide-protein glycotransferase activity (GO:0004579) | 0.149853814 | 0.3143607 | -2.542604 | 4.8261037 |
| NAD binding (GO:0051287) | 0.052352386 | 0.2080786 | -1.620841 | 4.7810887 |
| single-stranded DNA endodeoxyribonuclease activity (GO:0000014) | 0.121791305 | 0.3038304 | -2.260074 | 4.7584635 |
| AU-rich element binding (GO:0017091) | 0.048301469 | 0.1974728 | -1.558243 | 4.7219324 |
| cation-transporting ATPase activity (GO:0019829) | 0.083706419 | 0.2739745 | -1.889745 | 4.6873974 |
| phosphatase activator activity (GO:0019211) | 0.149853814 | 0.3143607 | -2.467019 | 4.682637 |
| chromo shadow domain binding (GO:0070087) | 0.107415665 | 0.3038304 | -2.08104 | 4.6429031 |
| mechanically-gated ion channel activity (GO:0008381) | 0.107415665 | 0.3038304 | -2.068661 | 4.6152852 |
| transmembrane receptor protein serine/threonine kinase binding (GO:0070696) | 0.029967122 | 0.1428691 | -1.311396 | 4.5999256 |
| RNA polymerase II distal enhancer sequence-specific DNA binding (GO:0000980) | 0.053703021 | 0.2098893 | -1.547654 | 4.5257822 |
| acetylcholine receptor regulator activity (GO:0030548) | 0.135936123 | 0.3143607 | -2.23394 | 4.4579831 |
| insulin-like growth factor II binding (GO:0031995) | 0.121791305 | 0.3038304 | -2.107912 | 4.4380958 |
| bHLH transcription factor binding (GO:0043425) | 0.052352386 | 0.2080786 | -1.496929 | 4.4155788 |
| ATPase coupled ion transmembrane transporter activity (GO:0042625) | 0.060798333 | 0.2281153 | -1.574684 | 4.4094181 |
| chitinase activity (GO:0004568) | 0.121791305 | 0.3038304 | -2.089234 | 4.3987691 |
| potassium ion symporter activity (GO:0022820) | 0.149853814 | 0.3143607 | -2.313753 | 4.3917224 |
| potassium:chloride symporter activity (GO:0015379) | 0.149853814 | 0.3143607 | -2.311898 | 4.3882027 |
| sodium channel inhibitor activity (GO:0019871) | 0.107415665 | 0.3038304 | -1.950544 | 4.3517596 |
| MHC class II protein binding (GO:0042289) | 0.107415665 | 0.3038304 | -1.925798 | 4.2965496 |
| exodeoxyribonuclease activity, producing 5'-phosphomonoesters (GO:0016895) | 0.163548015 | 0.3143607 | -2.365494 | 4.283078 |
| metal ion transmembrane transporter activity (GO:0046873) | 0.065183948 | 0.2321951 | -1.562936 | 4.2676612 |
| DNA-dependent ATPase activity (GO:0008094) | 0.030157877 | 0.1428691 | -1.215945 | 4.2573981 |
| DEAD/H-box RNA helicase binding (GO:0017151) | 0.121791305 | 0.3038304 | -2.006353 | 4.2242696 |
| calcium-dependent protein kinase activity (GO:0010857) | 0.135936123 | 0.3143607 | -2.114548 | 4.2197288 |
| rRNA methyltransferase activity (GO:0008649) | 0.135936123 | 0.3143607 | -2.099828 | 4.1903541 |
| histone kinase activity (GO:0035173) | 0.149853814 | 0.3143607 | -2.203795 | 4.1830119 |
| cytoskeletal adaptor activity (GO:0008093) | 0.149853814 | 0.3143607 | -2.200718 | 4.1771721 |
| voltage-gated potassium channel activity involved in ventricular cardiac muscle cell action potential repolarization (GO:1902282) | 0.135936123 | 0.3143607 | -2.089185 | 4.1691163 |
| thyroid hormone receptor binding (GO:0046966) | 0.069671916 | 0.2438517 | -1.563461 | 4.1649951 |
| protein serine/threonine kinase activity (GO:0004674) | 0.016957425 | 0.1146663 | -1.018999 | 4.1545096 |
| phosphatidylinositol phosphate 4-phosphatase activity (GO:0034596) | 0.121791305 | 0.3038304 | -1.967772 | 4.1430381 |
| U1 snRNA binding (GO:0030619) | 0.121791305 | 0.3038304 | -1.958532 | 4.1235831 |
| ribosomal protein S6 kinase activity (GO:0004711) | 0.121791305 | 0.3038304 | -1.937625 | 4.0795654 |
| double-stranded DNA binding (GO:0003690) | 0.022832715 | 0.1246529 | -1.078819 | 4.0774627 |
| histone-arginine N-methyltransferase activity (GO:0008469) | 0.149853814 | 0.3143607 | -2.118301 | 4.0207358 |
| transcriptional repressor activity, RNA polymerase II core promoter proximal region sequence-specific binding (GO:0001078) | 0.021129875 | 0.1229054 | -1.041518 | 4.0172064 |
| calmodulin-dependent protein kinase activity (GO:0004683) | 0.074257828 | 0.2579772 | -1.531496 | 3.9822136 |
| epidermal growth factor receptor binding (GO:0005154) | 0.065183948 | 0.2321951 | -1.454313 | 3.9710618 |
| cation:cation antiporter activity (GO:0015491) | 0.121791305 | 0.3038304 | -1.839165 | 3.8722632 |
| clathrin adaptor activity (GO:0035615) | 0.163548015 | 0.3143607 | -2.124315 | 3.846389 |
| calcium ion binding (GO:0005509) | 0.041941397 | 0.1855709 | -1.203587 | 3.817155 |
| retinoid X receptor binding (GO:0046965) | 0.149853814 | 0.3143607 | -1.995147 | 3.7869795 |
| arginine N-methyltransferase activity (GO:0016273) | 0.163548015 | 0.3143607 | -2.087717 | 3.7801225 |
| transferrin receptor binding (GO:1990459) | 0.135936123 | 0.3143607 | -1.885846 | 3.7633373 |
| oligosaccharyl transferase activity (GO:0004576) | 0.163548015 | 0.3143607 | -2.065387 | 3.7396904 |
| nucleosomal DNA binding (GO:0031492) | 0.083706419 | 0.2739745 | -1.478808 | 3.6680936 |
| kinesin binding (GO:0019894) | 0.078937389 | 0.2682727 | -1.437103 | 3.6489485 |
| phosphoprotein phosphatase activity (GO:0004721) | 0.023654817 | 0.1246529 | -0.970664 | 3.6343492 |
| glucosidase activity (GO:0015926) | 0.135936123 | 0.3143607 | -1.803696 | 3.599401 |
| chloride transmembrane transporter activity (GO:0015108) | 0.064056055 | 0.2321951 | -1.30151 | 3.5765452 |
| protease binding (GO:0002020) | 0.043480114 | 0.1858067 | -1.136278 | 3.5627434 |
| C4-dicarboxylate transmembrane transporter activity (GO:0015556) | 0.121791305 | 0.3038304 | -1.688859 | 3.555801 |
| phospholipase activity (GO:0004620) | 0.026362339 | 0.1343906 | -0.977749 | 3.5549168 |
| activin binding (GO:0048185) | 0.163548015 | 0.3143607 | -1.942427 | 3.5170534 |
| tau-protein kinase activity (GO:0050321) | 0.163548015 | 0.3143607 | -1.940476 | 3.5135203 |
| cation:chloride symporter activity (GO:0015377) | 0.149853814 | 0.3143607 | -1.837341 | 3.487447 |
| syntaxin binding (GO:0019905) | 0.037165847 | 0.1660074 | -1.057115 | 3.480409 |
| armadillo repeat domain binding (GO:0070016) | 0.149853814 | 0.3143607 | -1.830024 | 3.4735594 |
| phosphotyrosine residue binding (GO:0001784) | 0.083706419 | 0.2739745 | -1.399208 | 3.4706512 |
| growth factor receptor binding (GO:0070851) | 0.063335387 | 0.2321951 | -1.250253 | 3.4498383 |
| phosphatase binding (GO:0019902) | 0.025549505 | 0.1331413 | -0.924303 | 3.3895458 |
| interleukin-1 binding (GO:0019966) | 0.107415665 | 0.3038304 | -1.493375 | 3.3317935 |
| RNA polymerase II transcription cofactor activity (GO:0001104) | 0.04842083 | 0.1974728 | -1.091303 | 3.3042746 |
| excitatory extracellular ligand-gated ion channel activity (GO:0005231) | 0.083706419 | 0.2739745 | -1.329537 | 3.2978367 |
| protein-arginine N-methyltransferase activity (GO:0016274) | 0.190280198 | 0.335494 | -1.958961 | 3.2504202 |
| protein tyrosine kinase activity (GO:0004713) | 0.032834515 | 0.1539939 | -0.949336 | 3.2431926 |
| adenylate cyclase binding (GO:0008179) | 0.163548015 | 0.3143607 | -1.777108 | 3.2177184 |
| 3'-5'-exodeoxyribonuclease activity (GO:0008296) | 0.163548015 | 0.3143607 | -1.775657 | 3.2150918 |
| neurotrophin binding (GO:0043121) | 0.149853814 | 0.3143607 | -1.692607 | 3.2127291 |
| nuclear hormone receptor binding (GO:0035257) | 0.064056055 | 0.2321951 | -1.16633 | 3.2050712 |
| histone demethylase activity (H3-K4 specific) (GO:0032453) | 0.135936123 | 0.3143607 | -1.605486 | 3.2038605 |
| vascular endothelial growth factor receptor binding (GO:0005172) | 0.149853814 | 0.3143607 | -1.671083 | 3.1718752 |
| filamin binding (GO:0031005) | 0.163548015 | 0.3143607 | -1.732513 | 3.1369721 |
| collagen binding (GO:0005518) | 0.053703021 | 0.2098893 | -1.070171 | 3.1294869 |
| oxidoreductase activity, acting on the CH-OH group of donors, NAD or NADP as acceptor (GO:0016616) | 0.055606912 | 0.2155097 | -1.057343 | 3.0551369 |
| bioactive lipid receptor activity (GO:0045125) | 0.135936123 | 0.3143607 | -1.522688 | 3.0386309 |
| regulatory region DNA binding (GO:0000975) | 0.029764737 | 0.1428691 | -0.863407 | 3.0343853 |
| hexosaminidase activity (GO:0015929) | 0.177022302 | 0.326864 | -1.748742 | 3.0279109 |
| 2-acylglycerol-3-phosphate O-acyltransferase activity (GO:0047144) | 0.149853814 | 0.3143607 | -1.565012 | 2.9705424 |
| death receptor binding (GO:0005123) | 0.163548015 | 0.3143607 | -1.623113 | 2.9388883 |
| GTPase activity (GO:0003924) | 0.028437805 | 0.1428691 | -0.819042 | 2.9158192 |
| inositol trisphosphate phosphatase activity (GO:0046030) | 0.163548015 | 0.3143607 | -1.599499 | 2.8961314 |
| platelet-derived growth factor binding (GO:0048407) | 0.177022302 | 0.326864 | -1.666752 | 2.8859462 |
| complement component C1q binding (GO:0001849) | 0.149853814 | 0.3143607 | -1.514249 | 2.8741879 |
| mRNA 3'-UTR AU-rich region binding (GO:0035925) | 0.177022302 | 0.326864 | -1.651234 | 2.8590787 |
| sequence-specific double-stranded DNA binding (GO:1990837) | 0.036062208 | 0.1658154 | -0.858082 | 2.8509874 |
| purine ribonucleoside triphosphate binding (GO:0035639) | 0.057560613 | 0.2177091 | -0.993181 | 2.8354489 |
| signal recognition particle binding (GO:0005047) | 0.135936123 | 0.3143607 | -1.4157 | 2.8251284 |
| ATPase activity, coupled (GO:0042623) | 0.067400364 | 0.237675 | -1.036176 | 2.7946744 |
| ion channel activity (GO:0005216) | 0.065351279 | 0.2321951 | -1.024003 | 2.7934576 |
| protein phosphorylated amino acid binding (GO:0045309) | 0.108754743 | 0.3038304 | -1.258293 | 2.7917245 |
| oxidoreductase activity, acting on the CH-NH2 group of donors, oxygen as acceptor (GO:0016641) | 0.228789902 | 0.36622 | -1.860219 | 2.7437316 |
| platelet-derived growth factor receptor binding (GO:0005161) | 0.203325166 | 0.3492885 | -1.675174 | 2.668467 |
| peptidase inhibitor activity (GO:0030414) | 0.119265837 | 0.3038304 | -1.231991 | 2.6197052 |
| voltage-gated potassium channel activity involved in cardiac muscle cell action potential repolarization (GO:0086008) | 0.190280198 | 0.335494 | -1.549809 | 2.5715322 |
| nucleoside-triphosphatase activity (GO:0017111) | 0.043367633 | 0.1858067 | -0.811395 | 2.5461929 |
| calcium-dependent protein serine/threonine kinase activity (GO:0009931) | 0.177022302 | 0.326864 | -1.461792 | 2.5310629 |
| RNA polymerase II basal transcription factor binding (GO:0001091) | 0.190280198 | 0.335494 | -1.522085 | 2.5255309 |
| endopeptidase regulator activity (GO:0061135) | 0.119265837 | 0.3038304 | -1.182005 | 2.5134163 |
| ligand-gated cation channel activity (GO:0099094) | 0.075253702 | 0.2595146 | -0.963852 | 2.4933784 |
| protein serine/threonine/tyrosine kinase activity (GO:0004712) | 0.216160616 | 0.3582308 | -1.625271 | 2.489482 |
| PDZ domain binding (GO:0030165) | 0.081154992 | 0.2738251 | -0.990167 | 2.4866997 |
| voltage-gated chloride channel activity (GO:0005247) | 0.190280198 | 0.335494 | -1.498061 | 2.4856696 |
| endocytic adaptor activity (GO:0098748) | 0.163548015 | 0.3143607 | -1.358843 | 2.4603873 |
| outward rectifier potassium channel activity (GO:0015271) | 0.216160616 | 0.3582308 | -1.601378 | 2.4528845 |
| benzodiazepine receptor activity (GO:0008503) | 0.163548015 | 0.3143607 | -1.350601 | 2.4454646 |
| frizzled binding (GO:0005109) | 0.124612708 | 0.3092241 | -1.156138 | 2.4077082 |
| protein tyrosine/serine/threonine phosphatase activity (GO:0008138) | 0.108754743 | 0.3038304 | -1.083325 | 2.4035296 |
| acylglycerol lipase activity (GO:0047372) | 0.177022302 | 0.326864 | -1.373758 | 2.3786337 |
| E-box binding (GO:0070888) | 0.108754743 | 0.3038304 | -1.071046 | 2.3762861 |
| 3',5'-cyclic-AMP phosphodiesterase activity (GO:0004115) | 0.228789902 | 0.36622 | -1.585599 | 2.3386816 |
| ephrin receptor activity (GO:0005003) | 0.253443135 | 0.3859248 | -1.685532 | 2.3135872 |
| 1-phosphatidylinositol binding (GO:0005545) | 0.216160616 | 0.3582308 | -1.498501 | 2.2953038 |
| receptor tyrosine kinase binding (GO:0030971) | 0.135472703 | 0.3143607 | -1.133012 | 2.2648738 |
| manganese ion binding (GO:0030145) | 0.113978725 | 0.3038304 | -1.035204 | 2.248197 |
| protein serine/threonine phosphatase activity (GO:0004722) | 0.078179767 | 0.2676373 | -0.875377 | 2.2311131 |
| cAMP binding (GO:0030552) | 0.190280198 | 0.335494 | -1.327757 | 2.2030916 |
| transcriptional repressor activity, RNA polymerase II activating transcription factor binding (GO:0098811) | 0.190280198 | 0.335494 | -1.306825 | 2.1683586 |
| peptidyl-prolyl cis-trans isomerase activity (GO:0003755) | 0.13001606 | 0.3143607 | -1.062783 | 2.1681805 |
| carbonate dehydratase activity (GO:0004089) | 0.190280198 | 0.335494 | -1.294695 | 2.1482327 |
| lipoprotein particle receptor activity (GO:0030228) | 0.228789902 | 0.36622 | -1.44417 | 2.1300805 |
| oxidoreductase activity, acting on NAD(P)H, oxygen as acceptor (GO:0050664) | 0.190280198 | 0.335494 | -1.283285 | 2.1293004 |
| 3'-5' exonuclease activity (GO:0008408) | 0.13001606 | 0.3143607 | -1.035116 | 2.1117372 |
| sulfuric ester hydrolase activity (GO:0008484) | 0.241216326 | 0.3771015 | -1.466776 | 2.0858454 |
| sodium channel activity (GO:0005272) | 0.140979536 | 0.3143607 | -1.060785 | 2.0782263 |
| androgen receptor binding (GO:0050681) | 0.152131788 | 0.3143607 | -1.100025 | 2.0713559 |
| RNA polymerase III activity (GO:0001056) | 0.241216326 | 0.3771015 | -1.455694 | 2.0700861 |
| cytokine receptor activity (GO:0004896) | 0.116897066 | 0.3038304 | -0.960607 | 2.0619057 |
| inhibitory extracellular ligand-gated ion channel activity (GO:0005237) | 0.228789902 | 0.36622 | -1.39607 | 2.0591348 |
| RNA stem-loop binding (GO:0035613) | 0.203325166 | 0.3492885 | -1.273416 | 2.0284868 |
| nitric-oxide synthase binding (GO:0050998) | 0.177022302 | 0.326864 | -1.15632 | 2.0021445 |
| acyl-CoA dehydrogenase activity (GO:0003995) | 0.228789902 | 0.36622 | -1.33518 | 1.9693253 |
| DNA helicase activity (GO:0003678) | 0.140979536 | 0.3143607 | -1.003877 | 1.9667367 |
| retinol dehydrogenase activity (GO:0004745) | 0.253443135 | 0.3859248 | -1.431962 | 1.9655338 |
| adenyl ribonucleotide binding (GO:0032559) | 0.084120113 | 0.2739745 | -0.762663 | 1.8879789 |
| aminoacyl-tRNA ligase activity (GO:0004812) | 0.146533542 | 0.3143607 | -0.967571 | 1.8582209 |
| phosphatidylinositol bisphosphate kinase activity (GO:0052813) | 0.110012325 | 0.3038304 | -0.840978 | 1.8561759 |
| GTP binding (GO:0005525) | 0.091343523 | 0.2954491 | -0.774091 | 1.8524999 |
| monovalent inorganic cation transmembrane transporter activity (GO:0015077) | 0.253443135 | 0.3859248 | -1.347981 | 1.8502603 |
| histone deacetylase activity (GO:0004407) | 0.140979536 | 0.3143607 | -0.943017 | 1.847502 |
| cytokine activity (GO:0005125) | 0.107954654 | 0.3038304 | -0.810605 | 1.8044435 |
| transaminase activity (GO:0008483) | 0.216160616 | 0.3582308 | -1.177116 | 1.8030284 |
| damaged DNA binding (GO:0003684) | 0.174911343 | 0.326864 | -1.032129 | 1.7994929 |
| retinoic acid receptor binding (GO:0042974) | 0.241216326 | 0.3771015 | -1.242086 | 1.7663222 |
| poly-pyrimidine tract binding (GO:0008187) | 0.288957595 | 0.4081961 | -1.419421 | 1.7621756 |
| Toll-like receptor binding (GO:0035325) | 0.149853814 | 0.3143607 | -0.915653 | 1.7379958 |
| neuropilin binding (GO:0038191) | 0.27731065 | 0.4051673 | -1.335018 | 1.7123162 |
| death domain binding (GO:0070513) | 0.163548015 | 0.3143607 | -0.941903 | 1.7054553 |
| L-glutamate transmembrane transporter activity (GO:0005313) | 0.216160616 | 0.3582308 | -1.104903 | 1.6924164 |
| poly(U) RNA binding (GO:0008266) | 0.241216326 | 0.3771015 | -1.187613 | 1.6888576 |
| RNA polymerase II regulatory region DNA binding (GO:0001012) | 0.108727405 | 0.3038304 | -0.757702 | 1.6812737 |
| 3',5'-cyclic-nucleotide phosphodiesterase activity (GO:0004114) | 0.288957595 | 0.4081961 | -1.351008 | 1.6772434 |
| proteasome binding (GO:0070628) | 0.288957595 | 0.4081961 | -1.348264 | 1.6738366 |
| DNA binding, bending (GO:0008301) | 0.253443135 | 0.3859248 | -1.205415 | 1.6545711 |
| sequence-specific DNA binding (GO:0043565) | 0.107002595 | 0.3038304 | -0.72593 | 1.6223826 |
| lysophosphatidic acid acyltransferase activity (GO:0042171) | 0.288957595 | 0.4081961 | -1.291585 | 1.6034705 |
| transmembrane-ephrin receptor activity (GO:0005005) | 0.253443135 | 0.3859248 | -1.164822 | 1.5988534 |
| mannose binding (GO:0005537) | 0.177022302 | 0.326864 | -0.922366 | 1.5970577 |
| ankyrin binding (GO:0030506) | 0.265473528 | 0.3977862 | -1.186164 | 1.5731387 |
| mitogen-activated protein kinase kinase kinase binding (GO:0031435) | 0.163548015 | 0.3143607 | -0.860169 | 1.5574643 |
| transcription factor activity, RNA polymerase II core promoter sequence-specific (GO:0000983) | 0.288957595 | 0.4081961 | -1.23573 | 1.5341287 |
| transmembrane receptor protein phosphatase activity (GO:0019198) | 0.228789902 | 0.36622 | -1.029017 | 1.5177502 |
| purine ribonucleoside binding (GO:0032550) | 0.11677737 | 0.3038304 | -0.702502 | 1.5086131 |
| protein transporter activity (GO:0008565) | 0.142182857 | 0.3143607 | -0.769358 | 1.5007425 |
| protein kinase A regulatory subunit binding (GO:0034237) | 0.265473528 | 0.3977862 | -1.129452 | 1.4979248 |
| receptor antagonist activity (GO:0048019) | 0.253443135 | 0.3859248 | -1.080658 | 1.483328 |
| low-density lipoprotein particle receptor binding (GO:0050750) | 0.288957595 | 0.4081961 | -1.194422 | 1.4828458 |
| voltage-gated anion channel activity (GO:0008308) | 0.265473528 | 0.3977862 | -1.067859 | 1.4162369 |
| I-SMAD binding (GO:0070411) | 0.163548015 | 0.3143607 | -0.765576 | 1.3861897 |
| protein deacetylase activity (GO:0033558) | 0.152131788 | 0.3143607 | -0.731298 | 1.3770405 |
| transmembrane receptor protein tyrosine phosphatase activity (GO:0005001) | 0.228789902 | 0.36622 | -0.932396 | 1.3752386 |
| transcription corepressor activity (GO:0003714) | 0.112529603 | 0.3038304 | -0.626964 | 1.3696281 |
| carbohydrate kinase activity (GO:0019200) | 0.203325166 | 0.3492885 | -0.858198 | 1.3670662 |
| ephrin receptor binding (GO:0046875) | 0.27731065 | 0.4051673 | -1.041865 | 1.3363138 |
| potassium channel regulator activity (GO:0015459) | 0.157771424 | 0.3143607 | -0.722148 | 1.3335239 |
| polypeptide N-acetylgalactosaminyltransferase activity (GO:0004653) | 0.300417411 | 0.4119759 | -1.104245 | 1.3279451 |
| Tat protein binding (GO:0030957) | 0.163548015 | 0.3143607 | -0.729198 | 1.3203205 |
| hydrogen ion transmembrane transporter activity (GO:0015078) | 0.204061912 | 0.3492885 | -0.82253 | 1.3072731 |
| histone deacetylase binding (GO:0042826) | 0.161254124 | 0.3143607 | -0.706942 | 1.2900097 |
| phosphatidylinositol bisphosphate binding (GO:1902936) | 0.169089719 | 0.323686 | -0.716935 | 1.2742272 |
| cyclic nucleotide binding (GO:0030551) | 0.300417411 | 0.4119759 | -1.037801 | 1.2480408 |
| antiporter activity (GO:0015297) | 0.253443135 | 0.3859248 | -0.907967 | 1.2462897 |
| symporter activity (GO:0015293) | 0.190280198 | 0.335494 | -0.721176 | 1.1966165 |
| DNA polymerase activity (GO:0034061) | 0.300417411 | 0.4119759 | -0.98375 | 1.1830409 |
| disordered domain specific binding (GO:0097718) | 0.322787594 | 0.4300778 | -1.031182 | 1.1660199 |
| calcium channel regulator activity (GO:0005246) | 0.355012788 | 0.4475833 | -1.119268 | 1.1591154 |
| core promoter proximal region DNA binding (GO:0001159) | 0.311693094 | 0.4188655 | -0.98932 | 1.1532864 |
| telomerase RNA binding (GO:0070034) | 0.311693094 | 0.4188655 | -0.973758 | 1.1351445 |
| immunoglobulin binding (GO:0019865) | 0.177022302 | 0.326864 | -0.644914 | 1.1166556 |
| N-acetyltransferase activity (GO:0008080) | 0.311693094 | 0.4188655 | -0.951713 | 1.1094467 |
| RNA polymerase II transcription factor activity, sequence-specific transcription regulatory region DNA binding (GO:0001133) | 0.27731065 | 0.4051673 | -0.846383 | 1.0855856 |
| transforming growth factor beta receptor binding (GO:0005160) | 0.163449678 | 0.3143607 | -0.599153 | 1.0852167 |
| guanyl ribonucleotide binding (GO:0032561) | 0.142712515 | 0.3143607 | -0.553902 | 1.0784048 |
| calcium-dependent cysteine-type endopeptidase activity (GO:0004198) | 0.300417411 | 0.4119759 | -0.885077 | 1.0643779 |
| SH2 domain binding (GO:0042169) | 0.355012788 | 0.4475833 | -1.006696 | 1.0425356 |
| GDP binding (GO:0019003) | 0.204061912 | 0.3492885 | -0.641107 | 1.0189313 |
| transcription coactivator binding (GO:0001223) | 0.216160616 | 0.3582308 | -0.657613 | 1.0072873 |
| repressing transcription factor binding (GO:0070491) | 0.215871747 | 0.3582308 | -0.638224 | 0.9784424 |
| death receptor activity (GO:0005035) | 0.333703813 | 0.4359529 | -0.887731 | 0.9742859 |
| 1-acylglycerol-3-phosphate O-acyltransferase activity (GO:0003841) | 0.265473528 | 0.3977862 | -0.718096 | 0.9523673 |
| acidic amino acid transmembrane transporter activity (GO:0015172) | 0.203325166 | 0.3492885 | -0.595234 | 0.9481772 |
| methylation-dependent protein binding (GO:0140034) | 0.209958339 | 0.356777 | -0.60469 | 0.943828 |
| oxidoreductase activity, acting on the aldehyde or oxo group of donors, NAD or NADP as acceptor (GO:0016620) | 0.344444607 | 0.4450262 | -0.869005 | 0.9262045 |
| lipoprotein particle receptor binding (GO:0070325) | 0.344444607 | 0.4450262 | -0.860196 | 0.9168159 |
| transcription cofactor binding (GO:0001221) | 0.344444607 | 0.4450262 | -0.852375 | 0.9084796 |
| RNA methyltransferase activity (GO:0008173) | 0.27731065 | 0.4051673 | -0.696962 | 0.8939356 |
| FK506 binding (GO:0005528) | 0.228789902 | 0.36622 | -0.601944 | 0.8878383 |
| RNA polymerase II carboxy-terminal domain kinase activity (GO:0008353) | 0.228789902 | 0.36622 | -0.581864 | 0.8582216 |
| serine-type endopeptidase inhibitor activity (GO:0004867) | 0.233693486 | 0.371533 | -0.582731 | 0.8471422 |
| phosphatidylinositol-3,5-bisphosphate binding (GO:0080025) | 0.300417411 | 0.4119759 | -0.687466 | 0.8267344 |
| serotonin receptor activity (GO:0099589) | 0.405359741 | 0.4800851 | -0.899963 | 0.8126489 |
| low-density lipoprotein receptor activity (GO:0005041) | 0.203325166 | 0.3492885 | -0.506926 | 0.8075066 |
| cation transmembrane transporter activity (GO:0008324) | 0.209750686 | 0.356777 | -0.508923 | 0.7948535 |
| ATP-dependent DNA helicase activity (GO:0004003) | 0.375642324 | 0.467311 | -0.793814 | 0.7772374 |
| ion channel inhibitor activity (GO:0008200) | 0.424383684 | 0.4975899 | -0.855938 | 0.7336393 |
| ubiquitin-like protein conjugating enzyme activity (GO:0061650) | 0.300417411 | 0.4119759 | -0.590548 | 0.7101822 |
| nucleobase-containing compound kinase activity (GO:0019205) | 0.395614021 | 0.4769742 | -0.760016 | 0.704775 |
| transition metal ion transmembrane transporter activity (GO:0046915) | 0.414948792 | 0.4883007 | -0.782826 | 0.688574 |
| lysophospholipase activity (GO:0004622) | 0.27731065 | 0.4051673 | -0.514302 | 0.6596521 |
| ferrous iron binding (GO:0008198) | 0.288957595 | 0.4081961 | -0.518077 | 0.6431795 |
| inward rectifier potassium channel activity (GO:0005242) | 0.300417411 | 0.4119759 | -0.533408 | 0.6414671 |
| estrogen receptor binding (GO:0030331) | 0.414948792 | 0.4883007 | -0.693602 | 0.6100924 |
| Rho guanyl-nucleotide exchange factor activity (GO:0005089) | 0.251597257 | 0.3859248 | -0.431047 | 0.5948125 |
| endodeoxyribonuclease activity (GO:0004520) | 0.442800835 | 0.5155517 | -0.726957 | 0.5922048 |
| 3'-5'-exoribonuclease activity (GO:0000175) | 0.405359741 | 0.4800851 | -0.633772 | 0.5722839 |
| Hsp90 protein binding (GO:0051879) | 0.395614021 | 0.4769742 | -0.601464 | 0.557747 |
| steroid hormone receptor binding (GO:0035258) | 0.352521947 | 0.4475833 | -0.520236 | 0.5424206 |
| phosphatidylinositol-3-phosphate binding (GO:0032266) | 0.405359741 | 0.4800851 | -0.597673 | 0.5396869 |
| DNA-directed DNA polymerase activity (GO:0003887) | 0.288957595 | 0.4081961 | -0.410988 | 0.5102319 |
| protein kinase inhibitor activity (GO:0004860) | 0.299391106 | 0.4119759 | -0.406168 | 0.4898401 |
| ATPase binding (GO:0051117) | 0.275514413 | 0.4051673 | -0.379506 | 0.4892272 |
| MHC protein binding (GO:0042287) | 0.395614021 | 0.4769742 | -0.519583 | 0.4818182 |
| anion channel activity (GO:0005253) | 0.293430806 | 0.4119759 | -0.391226 | 0.479688 |
| aldo-keto reductase (NADP) activity (GO:0004033) | 0.288957595 | 0.4081961 | -0.379268 | 0.4708518 |
| divalent inorganic cation transmembrane transporter activity (GO:0072509) | 0.469330872 | 0.5395004 | -0.613356 | 0.4639713 |
| metal ion binding (GO:0046872) | 0.179333728 | 0.3298334 | -0.264692 | 0.4548758 |
| peptide-lysine-N-acetyltransferase activity (GO:0061733) | 0.477891338 | 0.5479976 | -0.61239 | 0.4521718 |
| mitogen-activated protein kinase binding (GO:0051019) | 0.288957595 | 0.4081961 | -0.362536 | 0.4500792 |
| cytokine receptor binding (GO:0005126) | 0.183140991 | 0.335494 | -0.265128 | 0.4500543 |
| translation factor activity, RNA binding (GO:0008135) | 0.299391106 | 0.4119759 | -0.348443 | 0.4202233 |
| hydrolase activity, acting on carbon-nitrogen (but not peptide) bonds, in linear amides (GO:0016811) | 0.346677638 | 0.4466808 | -0.39122 | 0.4144426 |
| histone acetyltransferase activity (GO:0004402) | 0.494601464 | 0.5616661 | -0.586792 | 0.4131031 |
| cysteine-type endopeptidase inhibitor activity (GO:0004869) | 0.204061912 | 0.3492885 | -0.255382 | 0.4058872 |
| histone methyltransferase activity (GO:0042054) | 0.186496142 | 0.335494 | -0.239823 | 0.4027461 |
| protein methyltransferase activity (GO:0008276) | 0.494601464 | 0.5616661 | -0.509964 | 0.3590158 |
| acylglycerol O-acyltransferase activity (GO:0016411) | 0.375642324 | 0.467311 | -0.353094 | 0.3457207 |
| NADP binding (GO:0050661) | 0.395614021 | 0.4769742 | -0.358193 | 0.3321584 |
| phosphatidylinositol-3,4-bisphosphate binding (GO:0043325) | 0.333703813 | 0.4359529 | -0.300356 | 0.3296407 |
| exoribonuclease activity, producing 5'-phosphomonoesters (GO:0016896) | 0.344444607 | 0.4450262 | -0.307232 | 0.3274544 |
| ADP binding (GO:0043531) | 0.322787594 | 0.4300778 | -0.280797 | 0.3175147 |
| phosphatidylinositol-4-phosphate binding (GO:0070273) | 0.311693094 | 0.4188655 | -0.245564 | 0.2862629 |
| miRNA binding (GO:0035198) | 0.333703813 | 0.4359529 | -0.250494 | 0.2749176 |
| calcium-dependent phospholipid binding (GO:0005544) | 0.54159995 | 0.5990811 | -0.442486 | 0.2713447 |
| hormone receptor binding (GO:0051427) | 0.486314133 | 0.5549424 | -0.374821 | 0.2702083 |
| G-protein coupled peptide receptor activity (GO:0008528) | 0.281491068 | 0.4081961 | -0.207821 | 0.2634452 |
| single-stranded RNA binding (GO:0003727) | 0.526439077 | 0.587857 | -0.396559 | 0.2544402 |
| L-amino acid transmembrane transporter activity (GO:0015179) | 0.556276912 | 0.6081442 | -0.417694 | 0.2449732 |
| lipase activity (GO:0016298) | 0.51077839 | 0.5744726 | -0.347081 | 0.2331759 |
| inorganic anion transmembrane transporter activity (GO:0015103) | 0.548997946 | 0.6015889 | -0.364345 | 0.2184833 |
| potassium ion transmembrane transporter activity (GO:0015079) | 0.469330872 | 0.5395004 | -0.275719 | 0.2085669 |
| phosphatase activity (GO:0016791) | 0.239371444 | 0.3771015 | -0.136788 | 0.1955715 |
| glutathione transferase activity (GO:0004364) | 0.395614021 | 0.4769742 | -0.208299 | 0.1931588 |
| acetylgalactosaminyltransferase activity (GO:0008376) | 0.548997946 | 0.6015889 | -0.321196 | 0.1926084 |
| ATP-dependent helicase activity (GO:0008026) | 0.404197122 | 0.4800851 | -0.203647 | 0.1844742 |
| iron ion binding (GO:0005506) | 0.590952068 | 0.6356801 | -0.341167 | 0.179461 |
| calcium ion transmembrane transporter activity (GO:0015085) | 0.35834805 | 0.450577 | -0.169259 | 0.1737026 |
| non-membrane spanning protein tyrosine kinase activity (GO:0004715) | 0.51077839 | 0.5744726 | -0.253703 | 0.1704424 |
| S-adenosylmethionine-dependent methyltransferase activity (GO:0008757) | 0.54159995 | 0.5990811 | -0.238517 | 0.1462651 |
| flavin adenine dinucleotide binding (GO:0050660) | 0.548997946 | 0.6015889 | -0.222039 | 0.1331481 |
| snoRNA binding (GO:0030515) | 0.395614021 | 0.4769742 | -0.138603 | 0.128529 |
| phosphatidylinositol 3-kinase activity (GO:0035004) | 0.352521947 | 0.4475833 | -0.120547 | 0.1256871 |
| mRNA binding (GO:0003729) | 0.329616167 | 0.4359529 | -0.112416 | 0.1247622 |
| hydro-lyase activity (GO:0016836) | 0.526439077 | 0.587857 | -0.173593 | 0.1113805 |
| neuropeptide hormone activity (GO:0005184) | 0.355012788 | 0.4475833 | -0.096363 | 0.0997942 |
| SH3 domain binding (GO:0017124) | 0.5975559 | 0.6413129 | -0.16996 | 0.0875135 |
| protein kinase regulator activity (GO:0019887) | 0.521143079 | 0.5847275 | -0.102148 | 0.066573 |
| RNA-dependent ATPase activity (GO:0008186) | 0.674327749 | 0.6950763 | -0.154861 | 0.0610212 |
| G-protein coupled serotonin receptor activity (GO:0004993) | 0.333703813 | 0.4359529 | -0.053427 | 0.0586362 |
| spectrin binding (GO:0030507) | 0.27731065 | 0.4051673 | -0.044593 | 0.0571957 |
| ATP-dependent microtubule motor activity, plus-end-directed (GO:0008574) | 0.405359741 | 0.4800851 | -0.057844 | 0.0522321 |
| ligand-dependent nuclear receptor transcription coactivator activity (GO:0030374) | 0.658020922 | 0.685804 | -0.110252 | 0.0461426 |
| cysteine-type peptidase activity (GO:0008234) | 0.658020922 | 0.685804 | -0.103718 | 0.043408 |
| histone acetyltransferase binding (GO:0035035) | 0.311693094 | 0.4188655 | -0.034652 | 0.0403954 |
| phosphatidylinositol binding (GO:0035091) | 0.485582271 | 0.5549424 | -0.043045 | 0.0310959 |
| Rab GTPase binding (GO:0017137) | 0.353141958 | 0.4475833 | -0.018771 | 0.0195381 |
| methylated histone binding (GO:0035064) | 0.227741295 | 0.36622 | -0.012701 | 0.018791 |
| microtubule binding (GO:0008017) | 0.388014932 | 0.4769742 | -0.012135 | 0.0114882 |
| O-acyltransferase activity (GO:0008374) | 0.405359741 | 0.4800851 | -0.01267 | 0.0114405 |
| ATP-dependent microtubule motor activity (GO:1990939) | 0.548997946 | 0.6015889 | -0.009021 | 0.0054098 |
| tubulin binding (GO:0015631) | 0.395225826 | 0.4769742 | 0.0122225 | -0.011346 |
| ubiquitin binding (GO:0043130) | 0.640900253 | 0.6739512 | 0.0320323 | -0.014251 |
| protein phosphatase binding (GO:0019903) | 0.443000747 | 0.5155517 | 0.0187379 | -0.015256 |
| translation initiation factor activity (GO:0003743) | 0.451787919 | 0.5231816 | 0.0207353 | -0.016475 |
| serine-type endopeptidase activity (GO:0004252) | 0.828825748 | 0.8413837 | 0.1820712 | -0.034183 |
| RNA polymerase II transcription factor binding (GO:0001085) | 0.587179205 | 0.6345324 | 0.0692356 | -0.036863 |
| G-protein coupled receptor activity (GO:0004930) | 0.808757684 | 0.8245812 | 0.2076238 | -0.044069 |
| ubiquitin-protein transferase activity (GO:0004842) | 0.907196309 | 0.9146582 | 0.4601273 | -0.044815 |
| endopeptidase activity (GO:0004175) | 0.927434348 | 0.9314062 | 0.6241513 | -0.047019 |
| motor activity (GO:0003774) | 0.753123278 | 0.7728989 | 0.1709352 | -0.048465 |
| integrin binding (GO:0005178) | 0.786818429 | 0.8039604 | 0.2622506 | -0.062877 |
| growth factor activity (GO:0008083) | 0.679589224 | 0.6989635 | 0.1777304 | -0.068651 |
| ubiquitin-like protein ligase activity (GO:0061659) | 0.952618248 | 0.9526182 | 1.4194362 | -0.068901 |
| cation channel activity (GO:0005261) | 0.908807462 | 0.9146582 | 0.8984432 | -0.085911 |
| peptidase activity, acting on L-amino acid peptides (GO:0070011) | 0.951835521 | 0.9526182 | 1.7428999 | -0.086035 |
| kinase inhibitor activity (GO:0019210) | 0.622925257 | 0.6594852 | 0.1851096 | -0.087618 |
| single-stranded DNA binding (GO:0003697) | 0.415419987 | 0.4883007 | 0.1034083 | -0.090841 |
| zinc ion binding (GO:0008270) | 0.362328088 | 0.4543633 | 0.0921966 | -0.093598 |
| magnesium ion binding (GO:0000287) | 0.875599418 | 0.8850348 | 0.7332691 | -0.097412 |
| methyltransferase activity (GO:0008168) | 0.668980147 | 0.691083 | 0.2448152 | -0.098416 |
| protein phosphatase regulator activity (GO:0019888) | 0.54159995 | 0.5990811 | 0.1658983 | -0.101733 |
| heme binding (GO:0020037) | 0.329045282 | 0.4359529 | 0.1191293 | -0.132419 |
| helicase activity (GO:0004386) | 0.433666888 | 0.5072064 | 0.1640038 | -0.137022 |
| serine-type peptidase activity (GO:0008236) | 0.873646994 | 0.8849686 | 1.0462321 | -0.141324 |
| transition metal ion binding (GO:0046914) | 0.3169596 | 0.4247259 | 0.12682 | -0.145714 |
| ATP-dependent RNA helicase activity (GO:0004004) | 0.668980147 | 0.691083 | 0.380368 | -0.152908 |
| carboxylic ester hydrolase activity (GO:0052689) | 0.815926988 | 0.8300862 | 0.8216814 | -0.167155 |
| GTPase binding (GO:0051020) | 0.352521947 | 0.4475833 | 0.1628629 | -0.169808 |
| cysteine-type endopeptidase activity (GO:0004197) | 0.38145235 | 0.4720347 | 0.1971179 | -0.189976 |
| DNA-directed 5'-3' RNA polymerase activity (GO:0003899) | 0.451787919 | 0.5231816 | 0.2442718 | -0.194084 |
| calcium channel activity (GO:0005262) | 0.783313299 | 0.8021265 | 0.8092334 | -0.197633 |
| transcription factor activity, RNA polymerase II distal enhancer sequence-specific binding (GO:0003705) | 0.652406418 | 0.682988 | 0.4690052 | -0.200306 |
| microtubule motor activity (GO:0003777) | 0.629014428 | 0.6644319 | 0.4380698 | -0.20309 |
| ATP binding (GO:0005524) | 0.232061699 | 0.3701937 | 0.1429352 | -0.208793 |
| acetylglucosaminyltransferase activity (GO:0008375) | 0.563438759 | 0.6145413 | 0.3833136 | -0.219906 |
| phosphatidylinositol-4,5-bisphosphate 3-kinase activity (GO:0046934) | 0.305343247 | 0.4162965 | 0.1949709 | -0.231298 |
| myosin binding (GO:0017022) | 0.534080984 | 0.5949738 | 0.3823805 | -0.239832 |
| SH3/SH2 adaptor activity (GO:0005070) | 0.570485363 | 0.6193464 | 0.4462347 | -0.250457 |
| hormone activity (GO:0005179) | 0.392872736 | 0.4769742 | 0.3043577 | -0.284352 |
| amino acid transmembrane transporter activity (GO:0015171) | 0.604053444 | 0.6438661 | 0.5734109 | -0.289052 |
| voltage-gated potassium channel activity (GO:0005249) | 0.404197122 | 0.4800851 | 0.3201274 | -0.289988 |
| phosphatidylinositol-4,5-bisphosphate binding (GO:0005546) | 0.257575005 | 0.3909472 | 0.2236021 | -0.303304 |
| phosphoric ester hydrolase activity (GO:0042578) | 0.604053444 | 0.6438661 | 0.6208147 | -0.312948 |
| double-stranded RNA binding (GO:0003725) | 0.622925257 | 0.6594852 | 0.6622913 | -0.313482 |
| actin filament binding (GO:0051015) | 0.613347134 | 0.6522898 | 0.6769858 | -0.330927 |
| 5'-3' RNA polymerase activity (GO:0034062) | 0.395614021 | 0.4769742 | 0.3633282 | -0.33692 |
| small GTPase binding (GO:0031267) | 0.646700028 | 0.6785287 | 0.8721421 | -0.380143 |
| rRNA binding (GO:0019843) | 0.51077839 | 0.5744726 | 0.5860875 | -0.393745 |
| phosphatidylinositol phosphate binding (GO:1901981) | 0.38145235 | 0.4720347 | 0.444807 | -0.428691 |
| mRNA 3'-UTR binding (GO:0003730) | 0.275514413 | 0.4051673 | 0.3371113 | -0.434575 |
| dicarboxylic acid transmembrane transporter activity (GO:0005310) | 0.375642324 | 0.467311 | 0.4485016 | -0.439136 |
| tumor necrosis factor-activated receptor activity (GO:0005031) | 0.333703813 | 0.4359529 | 0.4043649 | -0.443791 |
| RNA helicase activity (GO:0003724) | 0.668980147 | 0.691083 | 1.1674193 | -0.469304 |
| G-protein coupled amine receptor activity (GO:0008227) | 0.604053444 | 0.6438661 | 0.9775646 | -0.492783 |
| activating transcription factor binding (GO:0033613) | 0.305343247 | 0.4162965 | 0.5045401 | -0.598545 |
| polyubiquitin modification-dependent protein binding (GO:0031593) | 0.51077839 | 0.5744726 | 0.8953847 | -0.601537 |
| secondary active transmembrane transporter activity (GO:0015291) | 0.663545014 | 0.690028 | 1.5444504 | -0.63347 |
| Ras GTPase binding (GO:0017016) | 0.347866004 | 0.446984 | 0.6078848 | -0.641889 |
| phosphatidylinositol kinase activity (GO:0052742) | 0.570485363 | 0.6193464 | 1.172907 | -0.658315 |
| ATPase activity (GO:0016887) | 0.640861028 | 0.6739512 | 1.5708315 | -0.69893 |
| GTPase regulator activity (GO:0030695) | 0.458416158 | 0.5295497 | 1.1072981 | -0.863668 |
| actin binding (GO:0003779) | 0.589911207 | 0.6356801 | 1.6997897 | -0.897121 |
| GTPase activator activity (GO:0005096) | 0.574257302 | 0.6220016 | 1.6315148 | -0.904965 |
| **KEGG** | | | | |
| Term | P-value | FDR | Z-score | Combined Score |
| Human papillomavirus infection | 2.20E-08 | 5.43E-06 | -12.33911 | 217.57723 |
| Long-term potentiation | 1.12E-07 | 1.24E-05 | -8.272351 | 132.39339 |
| Pathways in cancer | 1.50E-07 | 1.24E-05 | 6.5997483 | -103.6918 |
| Kaposi sarcoma-associated herpesvirus infection | 3.63E-07 | 2.24E-05 | -1.064131 | 15.780872 |
| MAPK signaling pathway | 1.55E-06 | 7.65E-05 | -6.021204 | 80.551831 |
| Calcium signaling pathway | 2.31E-06 | 9.50E-05 | -1.664761 | 21.607818 |
| T cell receptor signaling pathway | 5.36E-06 | 0.000147 | -20.89921 | 253.65894 |
| AGE-RAGE signaling pathway in diabetic complications | 4.89E-06 | 0.000147 | -7.483557 | 91.503936 |
| Epstein-Barr virus infection | 5.04E-06 | 0.000147 | -0.355775 | 4.3397183 |
| Human immunodeficiency virus 1 infection | 9.30E-06 | 0.0002296 | -9.116593 | 105.62317 |
| Th1 and Th2 cell differentiation | 1.75E-05 | 0.0003747 | -20.9095 | 229.03665 |
| Human cytomegalovirus infection | 1.82E-05 | 0.0003747 | -11.58681 | 126.45661 |
| NOD-like receptor signaling pathway | 3.30E-05 | 0.0006265 | -2.787632 | 28.768021 |
| Oxytocin signaling pathway | 3.89E-05 | 0.0006409 | -7.069711 | 71.785635 |
| Axon guidance | 3.89E-05 | 0.0006409 | -0.665508 | 6.7587271 |
| Long-term depression | 4.88E-05 | 0.0006836 | -71.14557 | 706.29111 |
| Dopaminergic synapse | 5.26E-05 | 0.0006836 | -4.026234 | 39.67066 |
| Regulation of actin cytoskeleton | 4.64E-05 | 0.0006836 | -3.775478 | 37.675849 |
| Wnt signaling pathway | 5.23E-05 | 0.0006836 | -3.748718 | 36.960105 |
| Th17 cell differentiation | 5.85E-05 | 0.0006892 | -57.0713 | 556.26174 |
| Hippo signaling pathway | 5.86E-05 | 0.0006892 | -1.249141 | 12.172753 |
| ErbB signaling pathway | 6.79E-05 | 0.0007625 | -10.61374 | 101.86329 |
| Measles | 8.17E-05 | 0.0008769 | -6.051803 | 56.966037 |
| Signaling pathways regulating pluripotency of stem cells | 8.67E-05 | 0.0008928 | -6.577294 | 61.514314 |
| Phospholipase D signaling pathway | 0.000146053 | 0.0013875 | -6.032318 | 53.274655 |
| Retrograde endocannabinoid signaling | 0.000146053 | 0.0013875 | -0.49605 | 4.3808888 |
| cAMP signaling pathway | 0.000175946 | 0.0016096 | 12.817542 | -110.8119 |
| Phosphatidylinositol signaling system | 0.000198925 | 0.001646 | -8.502852 | 72.466281 |
| Choline metabolism in cancer | 0.000198925 | 0.001646 | -7.531807 | 64.190466 |
| Inflammatory mediator regulation of TRP channels | 0.000213246 | 0.001646 | -4.368181 | 36.924518 |
| HIF-1 signaling pathway | 0.000213246 | 0.001646 | -4.228244 | 35.741624 |
| Hepatitis C | 0.000213098 | 0.001646 | -0.583192 | 4.9301638 |
| Human T-cell leukemia virus 1 infection | 0.000237611 | 0.0017785 | 17.054747 | -142.3198 |
| Toll-like receptor signaling pathway | 0.000279253 | 0.001916 | -35.50777 | 290.57408 |
| C-type lectin receptor signaling pathway | 0.000279253 | 0.001916 | -9.707892 | 79.443501 |
| Cellular senescence | 0.000275409 | 0.001916 | -0.833096 | 6.8290941 |
| Necroptosis | 0.000304276 | 0.0020313 | -8.54916 | 69.227455 |
| cGMP-PKG signaling pathway | 0.000369634 | 0.0024026 | -2.271414 | 17.950982 |
| TNF signaling pathway | 0.000408783 | 0.0025242 | -52.65129 | 410.80246 |
| Hepatocellular carcinoma | 0.000406461 | 0.0025242 | -0.714821 | 5.5813395 |
| Nicotine addiction | 0.000434596 | 0.0026182 | -10.27363 | 79.529144 |
| Rap1 signaling pathway | 0.000532794 | 0.0031333 | 33.979306 | -256.1148 |
| Gap junction | 0.000546828 | 0.0031411 | -8.278149 | 62.180287 |
| Neurotrophin signaling pathway | 0.000690942 | 0.0038787 | -10.73512 | 78.124334 |
| GnRH signaling pathway | 0.00076221 | 0.0041345 | -13.05399 | 93.718343 |
| Amphetamine addiction | 0.000786721 | 0.0041345 | -8.550104 | 61.11304 |
| Epithelial cell signaling in Helicobacter pylori infection | 0.000786721 | 0.0041345 | -4.488353 | 32.081118 |
| NF-kappa B signaling pathway | 0.000865129 | 0.0044518 | -15.12798 | 106.69208 |
| Osteoclast differentiation | 0.001057446 | 0.0052238 | -4.877185 | 33.417976 |
| Aldosterone synthesis and secretion | 0.001039733 | 0.0052238 | -3.598662 | 24.718457 |
| Inositol phosphate metabolism | 0.001228658 | 0.0059506 | -8.193331 | 54.910339 |
| Pancreatic cancer | 0.001317738 | 0.0061412 | -11.75871 | 77.981866 |
| Glioma | 0.001317738 | 0.0061412 | -5.812874 | 38.550039 |
| Antigen processing and presentation | 0.001510618 | 0.0069097 | -13.63613 | 88.569904 |
| Viral carcinogenesis | 0.001608757 | 0.0072248 | 4.1436945 | -26.65346 |
| Influenza A | 0.001856184 | 0.0081871 | 0.0254666 | -0.160165 |
| Tuberculosis | 0.00253121 | 0.0109686 | 5.4791597 | -32.76021 |
| VEGF signaling pathway | 0.002578744 | 0.0109819 | -12.58167 | 74.992454 |
| Breast cancer | 0.002669047 | 0.0111738 | -2.440998 | 14.465435 |
| African trypanosomiasis | 0.002865227 | 0.0117952 | -50.37683 | 294.96176 |
| Cushing syndrome | 0.003691578 | 0.0149479 | 1.3161894 | -7.3729 |
| Ferroptosis | 0.003817978 | 0.0149689 | -41.89443 | 233.26963 |
| Lysosome | 0.003801363 | 0.0149689 | 0.2577106 | -1.436066 |
| Central carbon metabolism in cancer | 0.003934911 | 0.0151036 | -23.38177 | 129.48512 |
| Platelet activation | 0.003974636 | 0.0151036 | 4.8791945 | -26.97132 |
| Renin secretion | 0.005082784 | 0.0171632 | -61.68475 | 325.81245 |
| RIG-I-like receptor signaling pathway | 0.005403191 | 0.0171632 | -45.22711 | 236.12015 |
| Prolactin signaling pathway | 0.005403191 | 0.0171632 | -6.103486 | 31.864868 |
| Fc epsilon RI signaling pathway | 0.004776073 | 0.0171632 | -3.765532 | 20.123519 |
| Progesterone-mediated oocyte maturation | 0.005324733 | 0.0171632 | -3.327706 | 17.421846 |
| Hepatitis B | 0.00499393 | 0.0171632 | 0.9171979 | -4.86072 |
| Relaxin signaling pathway | 0.005143158 | 0.0171632 | 1.2218499 | -6.439257 |
| Focal adhesion | 0.005082861 | 0.0171632 | 1.5212217 | -8.034912 |
| RNA transport | 0.005368598 | 0.0171632 | 3.0133293 | -15.75124 |
| Natural killer cell mediated cytotoxicity | 0.00536059 | 0.0171632 | 8.8765702 | -46.41276 |
| Protein processing in endoplasmic reticulum | 0.005368598 | 0.0171632 | 14.692035 | -76.79804 |
| Proteoglycans in cancer | 0.005419969 | 0.0171632 | 37.006318 | -193.0866 |
| PI3K-Akt signaling pathway | 0.005079973 | 0.0171632 | 112.35714 | -593.5209 |
| FoxO signaling pathway | 0.005584847 | 0.0174615 | 0.4905692 | -2.544925 |
| Melanogenesis | 0.005864554 | 0.0181068 | -1.122653 | 5.7691234 |
| Glucagon signaling pathway | 0.006443243 | 0.0194083 | -1.03315 | 5.2119534 |
| Endocytosis | 0.006387913 | 0.0194083 | 89.082496 | -450.1648 |
| Estrogen signaling pathway | 0.006813044 | 0.0197979 | 0.4749081 | -2.369277 |
| Insulin signaling pathway | 0.006813044 | 0.0197979 | 0.5888707 | -2.937827 |
| Apelin signaling pathway | 0.006813044 | 0.0197979 | 0.6713512 | -3.349315 |
| Synaptic vesicle cycle | 0.008497032 | 0.0239683 | -45.88227 | 218.76841 |
| Vibrio cholerae infection | 0.008488221 | 0.0239683 | -13.19134 | 62.910496 |
| Apoptosis | 0.00853931 | 0.0239683 | 0.5834131 | -2.778841 |
| Amyotrophic lateral sclerosis (ALS) | 0.009096308 | 0.0252448 | -98.17144 | 461.39462 |
| Toxoplasmosis | 0.009973332 | 0.0270705 | -7.358614 | 33.907318 |
| Serotonergic synapse | 0.009973332 | 0.0270705 | -4.756262 | 21.916098 |
| Glutamatergic synapse | 0.01038919 | 0.0278927 | -1.210426 | 5.5280035 |
| Thyroid hormone signaling pathway | 0.011257581 | 0.0298992 | -8.24903 | 37.011036 |
| mTOR signaling pathway | 0.011703448 | 0.030429 | 2.553797 | -11.35896 |
| Phagosome | 0.011703448 | 0.030429 | 47.560062 | -211.5411 |
| Colorectal cancer | 0.012642186 | 0.0322234 | -20.64917 | 90.251677 |
| Sphingolipid signaling pathway | 0.012654524 | 0.0322234 | -4.939095 | 21.582562 |
| Ras signaling pathway | 0.013178019 | 0.033214 | 112.32815 | -486.2916 |
| Circadian rhythm | 0.013317266 | 0.0332259 | -45.42052 | 196.15734 |
| GABAergic synapse | 0.014496689 | 0.0358068 | -4.881814 | 20.668797 |
| TGF-beta signaling pathway | 0.015153264 | 0.0369191 | -14.00724 | 58.683869 |
| Cell cycle | 0.015245928 | 0.0369191 | 4.1382379 | -17.31208 |
| Glycerolipid metabolism | 0.016772115 | 0.0402205 | -30.14088 | 123.21704 |
| Small cell lung cancer | 0.017241486 | 0.0409485 | -28.38242 | 115.24504 |
| Basal cell carcinoma | 0.018675278 | 0.0435169 | -40.32949 | 160.53374 |
| Cytosolic DNA-sensing pathway | 0.018675278 | 0.0435169 | -28.56201 | 113.69264 |
| Circadian entrainment | 0.020310438 | 0.0467372 | 1.9697182 | -7.675244 |
| Tight junction | 0.020435708 | 0.0467372 | 14.792789 | -57.55093 |
| Cytokine-cytokine receptor interaction | 0.021284025 | 0.0482308 | 204.02081 | -785.439 |
| Spliceosome | 0.021488795 | 0.0482521 | 5.0315852 | -19.32241 |
| Non-small cell lung cancer | 0.02177193 | 0.0484474 | -7.671435 | 29.359609 |
| MicroRNAs in cancer | 0.023577395 | 0.0519966 | 206.02121 | -772.0577 |
| Longevity regulating pathway | 0.024622017 | 0.0538198 | 0.7934341 | -2.93897 |
| Adipocytokine signaling pathway | 0.025164673 | 0.0540767 | -1.416681 | 5.2166636 |
| Fluid shear stress and atherosclerosis | 0.025177414 | 0.0540767 | 1.8865476 | -6.945906 |
| B cell receptor signaling pathway | 0.027593666 | 0.0587555 | -1.639978 | 5.8877989 |
| Bladder cancer | 0.028089464 | 0.0593 | -21.6966 | 77.508089 |
| Parathyroid hormone synthesis, secretion and action | 0.02846516 | 0.0595839 | 3.5142032 | -12.50731 |
| Adrenergic signaling in cardiomyocytes | 0.030133067 | 0.0620239 | 3.0708268 | -10.75444 |
| Cell adhesion molecules (CAMs) | 0.030133067 | 0.0620239 | 4.4924707 | -15.73323 |
| Insulin resistance | 0.030521321 | 0.0623039 | 0.8881466 | -3.099036 |
| Gastric acid secretion | 0.032858293 | 0.0665246 | -1.460634 | 4.9888717 |
| Gastric cancer | 0.033768797 | 0.0678121 | 3.2524286 | -11.01994 |
| Leukocyte transendothelial migration | 0.034907833 | 0.0689779 | 0.9580474 | -3.214291 |
| Cholinergic synapse | 0.034907833 | 0.0689779 | 2.04045 | -6.8458 |
| Glycosaminoglycan degradation | 0.036896064 | 0.072328 | -379.0276 | 1250.6587 |
| Type II diabetes mellitus | 0.037737745 | 0.0733955 | -7.546114 | 24.729329 |
| N-Glycan biosynthesis | 0.046517499 | 0.0897642 | -6.743692 | 20.689151 |
| JAK-STAT signaling pathway | 0.047494087 | 0.0909383 | 3.6782154 | -11.20807 |
| Hypertrophic cardiomyopathy (HCM) | 0.04842083 | 0.0919996 | -3.182266 | 9.6353458 |
| Salmonella infection | 0.05016613 | 0.0938715 | -19.87056 | 59.460967 |
| Insulin secretion | 0.05016613 | 0.0938715 | -2.609562 | 7.8088922 |
| Oocyte meiosis | 0.051749824 | 0.0961068 | 37.28685 | -110.4188 |
| Salivary secretion | 0.057488477 | 0.1059676 | -10.56662 | 30.180068 |
| Legionellosis | 0.058771257 | 0.1063243 | -48.28235 | 136.83714 |
| Rheumatoid arthritis | 0.059403835 | 0.1063243 | -6.859305 | 19.366538 |
| mRNA surveillance pathway | 0.059403835 | 0.1063243 | -4.528658 | 12.786198 |
| Morphine addiction | 0.059403835 | 0.1063243 | -2.787106 | 7.8691041 |
| Vascular smooth muscle contraction | 0.062481643 | 0.1110285 | 14.924371 | -41.38353 |
| IL-17 signaling pathway | 0.063335387 | 0.1117417 | -4.88973 | 13.492286 |
| Endometrial cancer | 0.066778011 | 0.1169799 | -117.1018 | 316.92206 |
| Collecting duct acid secretion | 0.069671916 | 0.1211899 | -24.36297 | 64.901932 |
| Butanoate metabolism | 0.074257828 | 0.1273728 | -13.18101 | 34.273416 |
| Pancreatic secretion | 0.073744943 | 0.1273728 | 23.58507 | -61.48965 |
| Caffeine metabolism | 0.077956843 | 0.1318859 | -13.58497 | 34.663402 |
| Neomycin, kanamycin and gentamicin biosynthesis | 0.077956843 | 0.1318859 | -5.723741 | 14.604697 |
| Thermogenesis | 0.080669824 | 0.1355473 | 6.5261911 | -16.42897 |
| Chagas disease (American trypanosomiasis) | 0.084964507 | 0.1417989 | 7.6283312 | -18.80782 |
| Shigellosis | 0.0872498 | 0.1436713 | -10.94869 | 26.703633 |
| Mitophagy | 0.0872498 | 0.1436713 | -5.953549 | 14.520587 |
| Galactose metabolism | 0.088560843 | 0.1448644 | -25.21069 | 61.112375 |
| Neuroactive ligand-receptor interaction | 0.097247288 | 0.1580268 | 264.14286 | -615.5844 |
| Fructose and mannose metabolism | 0.09851012 | 0.1590327 | -16.01475 | 37.115727 |
| Renal cell carcinoma | 0.099994369 | 0.1603806 | 23.582091 | -54.3011 |
| Arrhythmogenic right ventricular cardiomyopathy (ARVC) | 0.110012325 | 0.1741862 | 2.2290575 | -4.919893 |
| Melanoma | 0.110012325 | 0.1741862 | 9.0352126 | -19.94219 |
| DNA replication | 0.113978725 | 0.1793168 | -15.07463 | 32.738223 |
| Primary immunodeficiency | 0.119265837 | 0.1864472 | -2.900414 | 6.1674409 |
| Pertussis | 0.123938635 | 0.1925336 | 1.2783715 | -2.6692 |
| AMPK signaling pathway | 0.128732343 | 0.1987306 | 4.5349917 | -9.296823 |
| Homologous recombination | 0.140979536 | 0.2162854 | -23.17976 | 45.412404 |
| Alzheimer disease | 0.142712515 | 0.2175925 | 5.9188803 | -11.5236 |
| ECM-receptor interaction | 0.145934453 | 0.2211399 | 1.1947169 | -2.299349 |
| Synthesis and degradation of ketone bodies | 0.149853814 | 0.2256945 | -5.634296 | 10.69443 |
| Type I diabetes mellitus | 0.152131788 | 0.2263648 | -3.882394 | 7.3105798 |
| Autophagy | 0.152013355 | 0.2263648 | 6.4491044 | -12.14874 |
| Glycosphingolipid biosynthesis | 0.163449678 | 0.2403099 | -473.6197 | 857.84368 |
| Proteasome | 0.163449678 | 0.2403099 | -0.676361 | 1.2250587 |
| Hedgehog signaling pathway | 0.174911343 | 0.2541359 | -27.1801 | 47.387857 |
| Nucleotide excision repair | 0.174911343 | 0.2541359 | -5.132467 | 8.9483334 |
| Endocrine and other factor-regulated calcium reabsorption | 0.180689595 | 0.2555297 | -74.90048 | 128.15283 |
| Notch signaling pathway | 0.180689595 | 0.2555297 | -5.028837 | 8.604212 |
| Valine, leucine and isoleucine degradation | 0.180689595 | 0.2555297 | -0.898119 | 1.5366592 |
| Fc gamma R-mediated phagocytosis | 0.181043341 | 0.2555297 | 0.7375555 | -1.260496 |
| Dilated cardiomyopathy (DCM) | 0.181043341 | 0.2555297 | 2.555542 | -4.367469 |
| Cocaine addiction | 0.186496142 | 0.2602517 | -14.27428 | 23.971431 |
| Malaria | 0.186496142 | 0.2602517 | -8.676826 | 14.571383 |
| Amoebiasis | 0.201443229 | 0.2795308 | 7.2834231 | -11.66985 |
| Glycerophospholipid metabolism | 0.205587245 | 0.2836874 | 4.3045737 | -6.80934 |
| Fanconi anemia pathway | 0.215871747 | 0.296224 | -9.365973 | 14.358699 |
| Pathogenic Escherichia coli infection | 0.221800067 | 0.3010144 | -3.980444 | 5.994465 |
| Regulation of lipolysis in adipocytes | 0.221800067 | 0.3010144 | -2.931354 | 4.4145568 |
| Ribosome | 0.233220372 | 0.3147838 | 17.032518 | -24.79545 |
| Nitrogen metabolism | 0.241216326 | 0.3238067 | -3.785236 | 5.3828371 |
| Viral myocarditis | 0.245623267 | 0.3279403 | 1.2431985 | -1.745396 |
| Pantothenate and CoA biosynthesis | 0.265473528 | 0.3525374 | -3.82314 | 5.0704021 |
| Inflammatory bowel disease (IBD) | 0.281491068 | 0.3698314 | -16.61092 | 21.056904 |
| Cortisol synthesis and secretion | 0.281491068 | 0.3698314 | -9.373361 | 11.882184 |
| Aminoacyl-tRNA biosynthesis | 0.28746367 | 0.37568 | -11.57551 | 14.430714 |
| Mismatch repair | 0.311693094 | 0.4030796 | -4.581089 | 5.3403414 |
| Proximal tubule bicarbonate reclamation | 0.311693094 | 0.4030796 | -1.186173 | 1.3827648 |
| p53 signaling pathway | 0.323138142 | 0.4135499 | 3.3877237 | -3.827028 |
| Adherens junction | 0.323138142 | 0.4135499 | 7.6310673 | -8.620629 |
| Alcoholism | 0.329616167 | 0.4196659 | 9.4397398 | -10.47647 |
| Leishmaniasis | 0.334938288 | 0.419948 | 4.4006098 | -4.813427 |
| PPAR signaling pathway | 0.334938288 | 0.419948 | 8.6754963 | -9.489336 |
| Thyroid hormone synthesis | 0.334938288 | 0.419948 | 12.382156 | -13.54371 |
| Maturity onset diabetes of the young | 0.344444607 | 0.429686 | -21.18705 | 22.581621 |
| Chronic myeloid leukemia | 0.346677638 | 0.4302984 | 16.097604 | -17.05316 |
| RNA degradation | 0.364155017 | 0.447494 | 7.6343586 | -7.712043 |
| Complement and coagulation cascades | 0.364155017 | 0.447494 | 13.138928 | -13.27262 |
| Chemokine signaling pathway | 0.366131581 | 0.4476955 | 13.158655 | -13.22132 |
| Huntington disease | 0.377081766 | 0.4588138 | 7.5823285 | -7.394994 |
| Ubiquitin mediated proteolysis | 0.379433575 | 0.4594122 | 28.059223 | -27.19151 |
| Citrate cycle (TCA cycle) | 0.38570908 | 0.4642331 | -4.542094 | 4.3271252 |
| Taste transduction | 0.38717418 | 0.4642331 | 9.3993188 | -8.918831 |
| Mucin type O-glycan biosynthesis | 0.395614021 | 0.4675438 | -3.291651 | 3.0524015 |
| RNA polymerase | 0.395614021 | 0.4675438 | 0.3785728 | -0.351057 |
| beta-Alanine metabolism | 0.395614021 | 0.4675438 | 2.5585829 | -2.372615 |
| Base excision repair | 0.414948792 | 0.4880588 | -7.593772 | 6.6794834 |
| Pentose and glucuronate interconversions | 0.424383684 | 0.4967904 | -2.54306 | 2.1797008 |
| Non-alcoholic fatty liver disease (NAFLD) | 0.43122502 | 0.5024178 | 7.1717821 | -6.032367 |
| Alanine, aspartate and glutamate metabolism | 0.433666888 | 0.5028907 | -7.544986 | 6.3036741 |
| Starch and sucrose metabolism | 0.442800835 | 0.5110832 | 4.0759686 | -3.320427 |
| Thyroid cancer | 0.451787919 | 0.5190308 | 2.6231523 | -2.084206 |
| Allograft rejection | 0.460630492 | 0.5263369 | -6.276394 | 4.8652039 |
| Hematopoietic cell lineage | 0.46454027 | 0.5263369 | 8.0471617 | -6.169815 |
| Prostate cancer | 0.46454027 | 0.5263369 | 27.408915 | -21.01461 |
| Pyruvate metabolism | 0.469330872 | 0.5293366 | 0.9793964 | -0.740862 |
| Graft-versus-host disease | 0.486314133 | 0.5459981 | -17.34824 | 12.506355 |
| Porphyrin and chlorophyll metabolism | 0.494601464 | 0.5527899 | 0.2204139 | -0.155172 |
| Carbohydrate digestion and absorption | 0.51077839 | 0.5657501 | -2.186105 | 1.4686677 |
| Fatty acid degradation | 0.51077839 | 0.5657501 | -2.010014 | 1.3503667 |
| ABC transporters | 0.518672224 | 0.5719287 | 0.6407116 | -0.420616 |
| Drug metabolism | 0.521143079 | 0.5720993 | 11.039284 | -7.19464 |
| Cysteine and methionine metabolism | 0.534080984 | 0.5811366 | 1.1438741 | -0.717447 |
| Sphingolipid metabolism | 0.534080984 | 0.5811366 | 7.7495373 | -4.86057 |
| Herpes simplex virus 1 infection | 0.538644899 | 0.583532 | 757.02798 | -468.3723 |
| Amino sugar and nucleotide sugar metabolism | 0.54159995 | 0.5841711 | -3.303915 | 2.0260523 |
| Arginine and proline metabolism | 0.548997946 | 0.5895761 | -8.573927 | 5.1414459 |
| Mineral absorption | 0.563438759 | 0.6024648 | -2.948359 | 1.6914633 |
| Autoimmune thyroid disease | 0.577418575 | 0.6132026 | -6.673468 | 3.6649877 |
| Transcriptional misregulation in cancer | 0.57844614 | 0.6132026 | 34.380241 | -18.82008 |
| Glutathione metabolism | 0.5975559 | 0.6307535 | -6.298826 | 3.2433122 |
| Pyrimidine metabolism | 0.604053444 | 0.6348987 | 2.8557816 | -1.439578 |
| Oxidative phosphorylation | 0.634172368 | 0.6609307 | 2.8451486 | -1.295779 |
| Systemic lupus erythematosus | 0.634172368 | 0.6609307 | 31.386386 | -14.29444 |
| Acute myeloid leukemia | 0.658020922 | 0.6829041 | -0.521314 | 0.2181796 |
| Glycolysis / Gluconeogenesis | 0.668980147 | 0.6884921 | 6.7151485 | -2.699496 |
| Staphylococcus aureus infection | 0.668980147 | 0.6884921 | 42.56791 | -17.11234 |
| Bacterial invasion of epithelial cells | 0.699801191 | 0.7172236 | 12.732193 | -4.544871 |
| Chemical carcinogenesis | 0.736490983 | 0.7517077 | 69.962316 | -21.39855 |
| Peroxisome | 0.740750944 | 0.7529444 | 6.9202125 | -2.076692 |
| Protein digestion and absorption | 0.768708822 | 0.7781602 | 6.3770562 | -1.67744 |
| Ribosome biogenesis in eukaryotes | 0.806693468 | 0.8132787 | 54.290776 | -11.66228 |
| Purine metabolism | 0.877615168 | 0.8811827 | 84.358176 | -11.01271 |
| Olfactory transduction | 0.999316479 | 0.9993165 | 817.33025 | -0.558854 |

**Table S4.** The overlapped 8 DEGs identified in the integrated analysis of GBM vs. NG, GBM vs. A, and GBM vs. OD tissues.

| **Gene symbol** | **Entrez ID** | **Gene name** | **FDR**  **GBM/NG training sets** | **FDR**  **GBM/NG test sets** | **FDR**  **GBM /A** | **FDR**  **GBM/OD** |
| --- | --- | --- | --- | --- | --- | --- |
| BARD1 | 580 | BRCA1 associated RING domain 1 | 2.10E-20 | 3.45E-20 | 1.10E-05 | 8.79E-04 |
| CBX3 | 11335 | chromobox 3 | 2.10E-20 | 3.45E-20 | 3.16E-20 | 2.88E-02 |
| CTSS | 1520 | cathepsin S | 2.10E-20 | 3.45E-20 | 6.17E-05 | 4.62E-20 |
| EGFR | 1956 | epidermal growth factor receptor | 2.10E-20 | 3.45E-20 | 3.16E-20 | 2.85E-02 |
| GUCY1A3 | 2982 | guanylate cyclase 1 soluble subunit alpha 3 | 2.10E-20 | 3.45E-20 | 9.71E-03 | 4.62E-20 |
| IFRD1 | 3475 | interferon related developmental regulator 1 | 2.10E-20 | 3.45E-20 | 3.16E-20 | 4.62E-20 |
| MOBP | 4336 | myelin-associated oligodendrocyte basic protein | 2.10E-20 | 3.45E-20 | 3.16E-20 | 1.07E-04 |
| STAT1 | 6772 | signal transducer and activator of transcription 1 | 2.10E-20 | 3.45E-20 | 3.16E-20 | 4.62E-20 |

Abbreviations: FDR, false discovery rate; GBM, glioblastoma; A, astrocytoma; OD, oligodendroglioma; NG, nonglioma.

**Table S5.** Eight differentially expressed genes validation results in TCGA-GBMLGG dataset

| **sample ID** | **Histology type** | **Overall survival (months)** | **vital state** | **BARD1** | **CBX3** | **CTSS** | **EGFR** | **GUCY1A3** | **IFRD1** | **MOBP** | **STAT1** |
| --- | --- | --- | --- | --- | --- | --- | --- | --- | --- | --- | --- |
| TCGA-HT-8015-01B-11R-A28M-07 | A | 0.10 | 0 | 38.15 | 1603.90 | 774.01 | 343.76 | 827.23 | 4628.96 | 1724.11 | 840.98 |
| TCGA-HT-7691-01A-11R-2256-07 | A | 0.10 | 0 | 82.36 | 2038.93 | 1084.18 | 736.03 | 874.12 | 3.81 | 1637.17 | 65.06 |
| TCGA-HT-7680-01A-11R-2256-07 | A | 0.77 | 0 | 275.82 | 2135.64 | 1308.93 | 1272.50 | 1745.99 | 2.66 | 2211.14 | 124.06 |
| TCGA-HT-7607-01A-11R-2090-07 | A | 3.20 | 1 | 43.10 | 1846.80 | 447.81 | 658.91 | 617.51 | 3026.94 | 2443.43 | 2429.63 |
| TCGA-VM-A8C9-01A-11R-A36H-07 | A | 43.80 | 0 | 2.55 | 2173.83 | 1251.19 | 101.24 | 759.63 | 1395.10 | 1620.50 | 74.50 |
| TCGA-P5-A5EY-01A-11R-A27Q-07 | A | 2.40 | 0 | 34.18 | 1854.08 | 473.98 | 647.44 | 571.43 | 229.59 | 2852.04 | 1063.78 |
| TCGA-E1-A7YN-01A-11R-A34F-07 | A | 24.23 | 1 | 90.16 | 3364.49 | 3109.95 | 3003.23 | 3820.78 | 5688.29 | 18041.23 | 190.21 |
| TCGA-QH-A6CS-01A-11R-A31N-07 | A | 19.60 | 0 | 7.14 | 2651.20 | 830.19 | 2691.97 | 709.84 | 15070.88 | 1473.74 | 150.43 |
| TCGA-DU-7290-01A-11R-2027-07 | A | 10.50 | 1 | 258.90 | 2304.01 | 1782.66 | 810.24 | 1600.17 | 1951.28 | 3368.31 | 262.13 |
| TCGA-HT-A74H-01A-11R-A32Q-07 | A | 2.47 | 0 | 9.54 | 3139.45 | 1169.83 | 448.34 | 2206.10 | 8559.31 | 1696.50 | 443.83 |
| TCGA-HT-7478-01A-11R-2027-07 | A | 6.47 | 0 | 98.80 | 3047.52 | 1596.24 | 1668.69 | 544.27 | 965.13 | 4868.38 | 631.45 |
| TCGA-DU-7012-01A-11R-2027-07 | A | 6.63 | 1 | 152.44 | 3845.57 | 4152.11 | 6040.51 | 1911.99 | 582.20 | 7727.00 | 667.22 |
| TCGA-DU-8158-01A-11R-2256-07 | A | 5.17 | 1 | 135.61 | 2473.82 | 2018.05 | 3455.69 | 1284.92 | 1490.61 | 3991.98 | 612.24 |
| TCGA-HW-7493-01A-11R-2027-07 | A | 70.23 | 0 | 79.65 | 2361.61 | 975.71 | 2882.09 | 730.78 | 345.68 | 2868.58 | 1721.23 |
| TCGA-HT-8110-01A-11R-2404-07 | A | 13.97 | 0 | 112.00 | 3197.43 | 2518.21 | 13699.28 | 2035.00 | 761.71 | 4443.10 | 531.64 |
| TCGA-HT-8563-01A-11R-2404-07 | A | 28.67 | 0 | 117.85 | 2308.16 | 5730.44 | 995.26 | 1394.18 | 122.25 | 13355.91 | 1585.51 |
| TCGA-S9-A7R2-01A-21R-A34R-07 | A | 10.53 | 1 | 45.10 | 2532.01 | 1903.98 | 948.54 | 1554.80 | 8047.53 | 1907.86 | 258.00 |
| TCGA-HW-A5KK-01A-11R-A27Q-07 | A | 12.93 | 1 | 79.77 | 2523.85 | 1712.99 | 7143.43 | 837.17 | 14137.34 | 2129.93 | 232.73 |
| TCGA-HT-7857-01A-11R-2403-07 | A | 0.23 | 0 | 66.63 | 2721.26 | 3182.44 | 49.97 | 757.53 | 509.76 | 4369.57 | 628.78 |
| TCGA-DU-7006-01A-11R-2027-07 | A | 11.63 | 1 | 190.59 | 4801.38 | 1887.20 | 415.32 | 1745.41 | 491.96 | 3296.21 | 233.64 |
| TCGA-CS-5397-01A-01R-1896-07 | A | 6.47 | 1 | 160.28 | 2881.72 | 1146.84 | 2407.90 | 1404.91 | 2819.56 | 2359.54 | 889.11 |
| TCGA-CS-4944-01A-01R-1470-07 | A | 60.93 | 0 | 22.19 | 2415.84 | 769.64 | 1741.52 | 796.87 | 1513.05 | 1560.96 | 649.10 |
| TCGA-FG-6688-01A-11R-1896-07 | A | 19.03 | 0 | 138.28 | 3987.06 | 1857.84 | 19100.44 | 3683.15 | 8018.49 | 3450.23 | 227.01 |
| TCGA-E1-A7YK-01A-11R-A34F-07 | A | 12.60 | 1 | 81.37 | 2405.81 | 1611.78 | 457.11 | 959.22 | 18622.51 | 3441.48 | 195.44 |
| TCGA-HT-8106-01A-11R-2404-07 | A | 0.10 | 0 | 182.52 | 2625.30 | 5423.55 | 811.60 | 930.44 | 7912.86 | 6908.16 | 614.57 |
| TCGA-DB-A75P-01A-11R-A32Q-07 | A | 16.40 | 0 | 50.02 | 1521.54 | 364.52 | 272.31 | 652.62 | 1898.10 | 1667.44 | 387.22 |
| TCGA-S9-A6UA-01A-12R-A33Z-07 | A | 5.93 | 1 | 153.79 | 3128.28 | 2973.41 | 65.02 | 735.90 | 179.66 | 3731.58 | 513.83 |
| TCGA-S9-A7R3-01A-11R-A34R-07 | A | 100.43 | 0 | 42.72 | 2328.22 | 1062.48 | 2179.32 | 1384.07 | 5673.74 | 1493.56 | 315.70 |
| TCGA-HT-A5R7-01A-11R-A28M-07 | A | 20.50 | 0 | 54.58 | 1990.66 | 273.85 | 819.15 | 928.31 | 3186.59 | 4693.24 | 837.82 |
| TCGA-E1-A7YM-01A-11R-A34F-07 | A | 21.60 | 1 | 67.49 | 3176.63 | 358.16 | 2511.47 | 1566.64 | 18217.83 | 1561.22 | 229.08 |
| TCGA-S9-A6WN-01A-12R-A33Z-07 | A | 26.83 | 0 | 115.42 | 1944.44 | 1981.64 | 622.37 | 772.62 | 136.88 | 6882.56 | 601.41 |
| TCGA-S9-A6WL-01A-21R-A33Z-07 | A | 31.53 | 0 | 78.45 | 2115.87 | 210.55 | 2749.66 | 811.09 | 97.39 | 2465.73 | 684.40 |
| TCGA-VM-A8CF-01A-11R-A36H-07 | A | 20.30 | 0 | 0.76 | 2324.02 | 1453.17 | 100.45 | 1722.81 | 5373.11 | 872.36 | 158.61 |
| TCGA-P5-A5F2-01A-11R-A28M-07 | A | 8.07 | 0 | 32.21 | 2213.09 | 189.43 | 2469.82 | 1181.79 | 2402.53 | 1135.63 | 326.94 |
| TCGA-E1-A7Z6-01A-11R-A34R-07 | A | 32.80 | 1 | 121.35 | 1783.63 | 574.79 | 1972.67 | 1633.58 | 246.47 | 1674.98 | 247.88 |
| TCGA-S9-A89V-01A-11R-A36H-07 | A | 18.97 | 0 | 102.11 | 2703.23 | 619.47 | 2456.70 | 627.04 | 517.75 | 3103.71 | 245.71 |
| TCGA-HT-7686-01A-11R-2256-07 | A | 43.33 | 0 | 127.24 | 2403.66 | 2156.86 | 2612.05 | 888.85 | 2069.10 | 3590.86 | 687.02 |
| TCGA-HT-7854-01A-11R-2256-07 | A | 40.03 | 0 | 178.90 | 2005.57 | 1411.41 | 7106.56 | 1482.53 | 3941.09 | 2764.98 | 1434.04 |
| TCGA-E1-5303-01A-01R-1470-07 | A | 68.40 | 1 | 151.66 | 2578.78 | 1442.26 | 3470.38 | 798.89 | 9747.58 | 1380.44 | 369.02 |
| TCGA-VM-A8CD-01A-11R-A36H-07 | A | 8.00 | 1 | 137.45 | 2359.33 | 1008.11 | 3353.01 | 1742.33 | 1008.45 | 28125.87 | 182.01 |
| TCGA-HT-7479-01A-11R-2027-07 | A | 40.90 | 0 | 85.26 | 2833.65 | 939.37 | 1349.73 | 1037.51 | 14234.56 | 1809.78 | 391.44 |
| TCGA-E1-A7Z3-01A-11R-A34R-07 | A | 74.50 | 1 | 30.32 | 1722.66 | 388.17 | 893.60 | 1132.70 | 1385.19 | 1541.75 | 292.25 |
| TCGA-E1-A7YH-01A-11R-A34F-07 | A | 94.50 | 1 | 158.59 | 2629.22 | 376.80 | 2576.68 | 1360.47 | 10004.39 | 1129.26 | 521.64 |
| TCGA-CS-4938-01B-11R-1896-07 | A | 119.13 | 0 | 99.43 | 2515.63 | 752.26 | 1595.13 | 1305.22 | 2842.88 | 1507.03 | 200.97 |
| TCGA-S9-A7R7-01A-11R-A34R-07 | A | 100.00 | 0 | 149.94 | 1826.09 | 1570.23 | 906.34 | 996.66 | 642.70 | 3752.51 | 520.07 |
| TCGA-S9-A7IX-01A-12R-A34F-07 | A | 27.30 | 1 | 129.55 | 2925.99 | 1253.77 | 15402.26 | 1364.21 | 1166.96 | 2666.88 | 792.55 |
| TCGA-FG-A87Q-01A-11R-A36H-07 | A | 5.80 | 0 | 83.21 | 2847.04 | 1205.28 | 36523.68 | 2254.11 | 11328.85 | 4678.13 | 214.75 |
| TCGA-P5-A5F1-01A-11R-A28M-07 | A | 5.97 | 0 | 44.63 | 2528.33 | 623.28 | 473.04 | 1950.17 | 912.85 | 10638.40 | 158.18 |
| TCGA-TM-A84C-01A-11R-A36H-07 | A | 16.40 | 1 | 147.70 | 2513.62 | 601.96 | 596.01 | 1088.69 | 266.76 | 5975.21 | 123.60 |
| TCGA-HT-7606-01A-11R-2090-07 | A | 17.53 | 0 | 98.46 | 5952.47 | 331.17 | 12044.74 | 1624.69 | 1887.96 | 2153.40 | 175.93 |
| TCGA-DU-5854-01A-11R-1708-07 | A | 8.57 | 0 | 187.71 | 2409.80 | 1337.89 | 26687.95 | 1142.97 | 14836.90 | 2962.29 | 578.32 |
| TCGA-DU-7299-01A-21R-2027-07 | A | 44.63 | 1 | 71.49 | 2692.91 | 772.42 | 2866.91 | 706.29 | 4248.19 | 2552.88 | 416.33 |
| TCGA-P5-A733-01A-11R-A32Q-07 | A | 13.80 | 0 | 43.34 | 3029.58 | 261.47 | 1304.89 | 1925.26 | 95.92 | 1396.96 | 509.80 |
| TCGA-QH-A6CX-01A-11R-A32Q-07 | A | 12.40 | 1 | 62.66 | 1852.87 | 875.52 | 13925.81 | 2140.98 | 9767.27 | 2661.26 | 846.43 |
| TCGA-S9-A6TZ-01A-21R-A32Q-07 | A | 37.33 | 0 | 38.95 | 2795.91 | 899.68 | 8530.76 | 1132.36 | 601.18 | 6167.12 | 2013.80 |
| TCGA-CS-6665-01A-11R-1896-07 | A | 52.27 | 0 | 141.33 | 3549.91 | 2223.35 | 246.39 | 1175.06 | 11281.82 | 3695.31 | 690.39 |
| TCGA-TM-A7C4-01A-11R-A32Q-07 | A | 49.00 | 0 | 35.52 | 2559.61 | 154.74 | 807.78 | 1130.41 | 197.57 | 1503.16 | 1105.11 |
| TCGA-HT-7476-01A-11R-2027-07 | A | 6.63 | 0 | 71.78 | 2982.67 | 455.86 | 1330.02 | 695.54 | 834.16 | 1896.45 | 898.93 |
| TCGA-DU-6392-01A-11R-1708-07 | A | 214.10 | 0 | 88.65 | 4312.34 | 935.64 | 686.96 | 591.74 | 1601.76 | 3369.56 | 379.85 |
| TCGA-HT-7601-01A-11R-2090-07 | A | 5.10 | 0 | 119.16 | 2626.47 | 2719.74 | 1871.07 | 711.06 | 11610.60 | 5136.27 | 1046.90 |
| TCGA-DB-A4XF-01A-11R-A27Q-07 | A | 34.03 | 0 | 233.93 | 2557.53 | 556.58 | 4798.55 | 777.22 | 539.98 | 2813.29 | 353.97 |
| TCGA-DU-7292-01A-11R-2027-07 | A | 8.07 | 1 | 141.49 | 1949.54 | 318.11 | 53761.60 | 933.57 | 1169.91 | 2668.80 | 1345.58 |
| TCGA-FG-6689-01A-11R-1896-07 | A | 15.13 | 0 | 96.80 | 2623.28 | 816.87 | 2250.48 | 722.37 | 5556.90 | 1609.88 | 589.27 |
| TCGA-TM-A84F-01A-11R-A36H-07 | A | 59.87 | 0 | 37.87 | 2613.89 | 1701.74 | 427.06 | 986.32 | 4485.53 | 6214.10 | 2338.24 |
| TCGA-S9-A6U9-01A-11R-A32Q-07 | A | 97.27 | 0 | 66.02 | 1559.52 | 1573.01 | 1475.93 | 1034.22 | 5121.45 | 1269.40 | 372.05 |
| TCGA-HT-7884-01B-11R-2403-07 | A | 11.43 | 0 | 108.02 | 2763.13 | 218.99 | 2802.55 | 1079.45 | 9233.55 | 1209.22 | 653.64 |
| TCGA-RY-A843-01A-11R-A36H-07 | A | 2.10 | 0 | 72.58 | 2573.35 | 439.32 | 2701.54 | 953.15 | 1085.25 | 1969.28 | 1007.30 |
| TCGA-HT-8564-01A-11R-2404-07 | A | 15.93 | 0 | 89.57 | 3044.50 | 524.76 | 6015.03 | 1089.66 | 2320.17 | 2998.79 | 1437.52 |
| TCGA-P5-A736-01A-11R-A32Q-07 | A | 7.67 | 0 | 44.85 | 1744.74 | 2071.98 | 775.73 | 1430.23 | 2052.05 | 1524.36 | 393.13 |
| TCGA-WY-A859-01A-12R-A36H-07 | A | 40.43 | 0 | 37.15 | 2088.19 | 157.97 | 610.87 | 736.23 | 1733.81 | 1017.28 | 515.91 |
| TCGA-FG-5963-01A-11R-1708-07 | A | 25.83 | 1 | 82.15 | 2921.23 | 2304.26 | 91.21 | 919.54 | 14128.13 | 4152.13 | 1223.12 |
| TCGA-HT-7485-01A-11R-2027-07 | A | 4.07 | 0 | 48.58 | 2667.28 | 886.05 | 3177.13 | 1315.19 | 4625.64 | 1677.87 | 362.67 |
| TCGA-HW-7490-01A-11R-2027-07 | A | 46.23 | 0 | 117.68 | 2925.66 | 502.79 | 1467.89 | 1675.00 | 200.36 | 11118.84 | 375.41 |
| TCGA-DU-A7TI-01A-11R-A33Z-07 | A | 39.43 | 1 | 67.34 | 2409.53 | 850.55 | 4248.31 | 1155.35 | 1200.39 | 8996.17 | 437.74 |
| TCGA-CS-6290-01A-11R-1708-07 | A | 37.90 | 1 | 55.18 | 2402.36 | 1430.29 | 1941.09 | 956.98 | 7810.58 | 8591.25 | 308.65 |
| TCGA-CS-6667-01A-12R-2027-07 | A | 48.97 | 0 | 120.85 | 2996.72 | 349.78 | 3180.58 | 1204.22 | 482.68 | 1185.43 | 199.88 |
| TCGA-HT-7860-01A-11R-2403-07 | A | 0.50 | 0 | 260.09 | 3345.82 | 1413.54 | 3339.30 | 1255.04 | 25.22 | 5280.98 | 369.24 |
| TCGA-E1-5307-01A-01R-1896-07 | A | 58.73 | 1 | 32.02 | 2902.73 | 640.10 | 9947.16 | 684.27 | 989.42 | 2290.24 | 612.50 |
| TCGA-E1-5302-01A-01R-1470-07 | A | 50.83 | 1 | 159.50 | 2058.25 | 1177.09 | 4129.39 | 962.86 | 1768.18 | 2250.59 | 241.60 |
| TCGA-DH-A7UT-01A-12R-A34F-07 | A | 17.70 | 1 | 63.55 | 2766.61 | 487.58 | 749.10 | 1414.21 | 11394.57 | 1143.85 | 1187.18 |
| TCGA-HT-A618-01A-11R-A29R-07 | A | 17.07 | 0 | 37.75 | 3475.27 | 2004.78 | 1086.74 | 1349.10 | 1602.39 | 3824.13 | 293.91 |
| TCGA-P5-A781-01A-11R-A32Q-07 | A | 4.47 | 0 | 39.30 | 3178.28 | 377.63 | 2498.34 | 1079.01 | 4763.37 | 1591.98 | 1741.49 |
| TCGA-DU-A7TC-01A-21R-A34R-07 | A | 37.90 | 0 | 21.88 | 2652.34 | 310.16 | 1665.18 | 876.56 | 895.70 | 1581.64 | 526.56 |
| TCGA-HT-A616-01A-11R-A29R-07 | A | 1.83 | 0 | 12.99 | 3067.23 | 227.54 | 719.16 | 1244.50 | 526.28 | 1335.90 | 830.79 |
| TCGA-DU-7013-01A-11R-2027-07 | A | 8.97 | 1 | 234.04 | 6164.51 | 1093.40 | 58123.01 | 1587.16 | 682.01 | 4787.40 | 264.08 |
| TCGA-FG-6691-01A-11R-1896-07 | A | 41.90 | 0 | 191.14 | 3526.34 | 1987.75 | 6115.49 | 922.81 | 501.31 | 2309.47 | 1012.78 |
| TCGA-HT-A5RC-01A-11R-A28M-07 | A | 5.40 | 1 | 201.82 | 3255.65 | 707.94 | 58640.32 | 1439.71 | 2832.23 | 2622.32 | 145.63 |
| TCGA-DB-A75O-01A-11R-A32Q-07 | A | 31.17 | 0 | 49.27 | 1910.26 | 871.55 | 1600.54 | 853.37 | 1906.74 | 2838.12 | 211.73 |
| TCGA-CS-5394-01A-01R-1470-07 | A | 0.27 | 0 | 132.82 | 3480.09 | 216.79 | 8346.92 | 807.29 | 84.64 | 1883.91 | 3594.18 |
| TCGA-DU-A5TY-01A-11R-A28M-07 | A | 34.43 | 1 | 85.86 | 17126.71 | 3120.36 | 39468.77 | 1386.05 | 1663.78 | 2826.85 | 373.23 |
| TCGA-E1-5305-01A-01R-1896-07 | A | 81.10 | 1 | 132.83 | 2968.81 | 513.11 | 6926.01 | 1043.14 | 1981.66 | 4392.22 | 561.29 |
| TCGA-F6-A8O4-01A-11R-A36H-07 | A | 0.20 | 0 | 70.51 | 2408.00 | 264.35 | 2704.47 | 1130.53 | 688.09 | 1367.01 | 391.60 |
| TCGA-HT-7858-01A-11R-2403-07 | A | 51.33 | 0 | 140.06 | 3212.20 | 1705.30 | 2796.19 | 1047.58 | 8863.78 | 1736.76 | 292.40 |
| TCGA-CS-5393-01A-01R-1470-07 | A | 40.73 | 0 | 71.41 | 5485.58 | 746.42 | 11177.29 | 855.36 | 6367.16 | 1249.95 | 577.77 |
| TCGA-S9-A6U8-01A-21R-A33Z-07 | A | 99.60 | 1 | 87.47 | 2686.54 | 1204.93 | 1968.74 | 940.38 | 8682.97 | 1761.16 | 539.81 |
| TCGA-S9-A6U6-01A-12R-A33Z-07 | A | 35.63 | 0 | 49.74 | 2640.46 | 401.97 | 4510.93 | 815.97 | 489.02 | 1289.34 | 323.34 |
| TCGA-HW-A5KM-01A-11R-A27Q-07 | A | 21.00 | 0 | 135.23 | 2488.12 | 5821.97 | 2099.88 | 785.59 | 520.68 | 23046.24 | 359.40 |
| TCGA-S9-A7R4-01A-12R-A34R-07 | A | 30.47 | 0 | 82.88 | 3195.44 | 978.70 | 2442.96 | 1174.25 | 331.04 | 2287.98 | 369.69 |
| TCGA-WY-A858-01A-11R-A36H-07 | A | 44.57 | 0 | 17.94 | 2823.98 | 1621.30 | 615.98 | 1184.13 | 3097.88 | 1315.70 | 229.05 |
| TCGA-CS-6188-01A-11R-1896-07 | A | 27.13 | 1 | 189.87 | 3761.30 | 846.65 | 20037.55 | 1532.01 | 2729.84 | 5005.43 | 657.87 |
| TCGA-S9-A6U0-01A-12R-A32Q-07 | A | 24.73 | 1 | 96.79 | 3099.56 | 1687.50 | 32086.49 | 1323.01 | 371.13 | 3529.31 | 1394.91 |
| TCGA-HT-7855-01A-11R-2403-07 | A | 19.50 | 0 | 233.67 | 3229.33 | 455.94 | 4246.64 | 935.03 | 11.40 | 1701.22 | 101.16 |
| TCGA-DB-5273-01A-01R-1470-07 | A | 83.10 | 0 | 51.80 | 2613.34 | 978.04 | 1315.79 | 567.34 | 2690.43 | 4339.41 | 417.32 |
| TCGA-FG-A6J3-01A-11R-A31N-07 | A | 22.57 | 0 | 3.20 | 3714.40 | 826.96 | 133.21 | 849.34 | 3821.50 | 3157.05 | 230.19 |
| TCGA-DU-7007-01A-11R-2027-07 | A | 63.83 | 1 | 152.39 | 2747.68 | 1035.19 | 918.66 | 1413.35 | 560.76 | 3489.38 | 691.24 |
| TCGA-P5-A5EV-01A-11R-A27Q-07 | A | 0.23 | 0 | 34.22 | 2671.90 | 642.11 | 2332.10 | 1400.97 | 628.42 | 1611.51 | 226.65 |
| TCGA-HT-8104-01A-11R-2404-07 | A | 12.40 | 0 | 265.83 | 3745.31 | 867.82 | 114753.47 | 1875.44 | 3952.81 | 5497.07 | 756.74 |
| TCGA-DU-A5TU-01A-11R-A28M-07 | A | 26.53 | 0 | 18.20 | 4657.22 | 915.79 | 278.46 | 1479.48 | 311.05 | 2406.69 | 495.98 |
| TCGA-DU-5847-01A-11R-1708-07 | A | 18.27 | 0 | 411.95 | 2589.77 | 1167.55 | 2677.83 | 1305.67 | 2840.24 | 4233.39 | 312.19 |
| TCGA-CS-4942-01A-01R-1470-07 | A | 44.50 | 1 | 144.64 | 3371.28 | 2347.40 | 3437.69 | 1109.00 | 2246.02 | 8284.78 | 667.47 |
| TCGA-S9-A6U5-01A-12R-A33Z-07 | A | 33.07 | 0 | 63.29 | 2705.06 | 1531.65 | 717.69 | 806.33 | 276.79 | 3332.07 | 1350.21 |
| TCGA-E1-A7YJ-01A-11R-A34F-07 | A | 19.73 | 1 | 171.97 | 3101.45 | 1064.17 | 57650.01 | 1949.39 | 2864.12 | 9345.92 | 91.38 |
| TCGA-FG-A60L-01A-12R-A31N-07 | A | 21.83 | 0 | 4.14 | 2767.20 | 872.74 | 201.22 | 1673.56 | 5583.03 | 1120.54 | 441.80 |
| TCGA-P5-A72W-01A-11R-A32Q-07 | A | 10.57 | 0 | 10.13 | 3157.97 | 339.75 | 881.01 | 1301.27 | 1002.03 | 947.34 | 74.43 |
| TCGA-E1-A7Z4-01A-11R-A34R-07 | A | 147.07 | 1 | 28.06 | 2975.29 | 854.27 | 1976.08 | 1280.15 | 161.64 | 1644.05 | 127.30 |
| TCGA-E1-A7YE-01A-11R-A34F-07 | A | 29.53 | 1 | 119.68 | 2924.34 | 1685.57 | 1783.88 | 1269.42 | 1062.95 | 2061.07 | 153.23 |
| TCGA-QH-A6XC-01A-12R-A32Q-07 | A | 16.93 | 0 | 33.20 | 3474.84 | 543.04 | 7544.46 | 1686.14 | 3474.84 | 2998.19 | 263.43 |
| TCGA-P5-A5EW-01A-11R-A27Q-07 | A | 6.80 | 0 | 145.31 | 2742.30 | 774.58 | 3960.67 | 813.09 | 1598.44 | 1438.22 | 380.46 |
| TCGA-HW-8321-01A-11R-2404-07 | A | 43.13 | 0 | 165.06 | 2870.78 | 557.97 | 4246.59 | 1149.28 | 1793.03 | 2022.49 | 364.11 |
| TCGA-DB-5277-01A-01R-1470-07 | A | 51.57 | 1 | 128.47 | 3474.90 | 623.38 | 1715.33 | 963.15 | 2812.57 | 3346.44 | 1087.05 |
| TCGA-FG-A87N-01A-11R-A36H-07 | A | 18.90 | 0 | 134.01 | 3311.59 | 557.37 | 443.49 | 1112.66 | 584.35 | 2545.80 | 191.52 |
| TCGA-HW-8319-01A-11R-2404-07 | A | 40.30 | 1 | 151.31 | 2491.61 | 494.46 | 5946.14 | 1060.15 | 1662.55 | 1886.99 | 724.28 |
| TCGA-S9-A6WO-01A-21R-A34F-07 | A | 18.87 | 0 | 122.67 | 2786.38 | 880.13 | 3258.27 | 1283.58 | 1275.65 | 1197.76 | 208.49 |
| TCGA-CS-4941-01A-01R-1470-07 | A | 7.80 | 1 | 136.30 | 2473.78 | 1554.52 | 48018.05 | 1350.24 | 1622.15 | 8622.84 | 408.56 |
| TCGA-FG-7636-01A-11R-2090-07 | A | 18.13 | 0 | 49.04 | 2728.90 | 397.46 | 1123.71 | 822.64 | 4252.63 | 1700.71 | 556.95 |
| TCGA-DB-A64X-01A-11R-A29R-07 | A | 18.77 | 0 | 25.22 | 3343.84 | 273.36 | 542.17 | 1432.35 | 77.17 | 1538.27 | 213.34 |
| TCGA-DU-A5TP-01A-11R-A28M-07 | A | 33.73 | 0 | 54.60 | 3598.93 | 1079.64 | 766.40 | 1997.13 | 550.90 | 5325.12 | 186.78 |
| TCGA-TQ-A7RV-01A-21R-A34F-07 | A | 62.27 | 0 | 51.17 | 2770.02 | 325.35 | 2324.28 | 866.06 | 3469.21 | 1718.38 | 274.56 |
| TCGA-VM-A8CH-01A-12R-A36H-07 | A | 23.80 | 0 | 116.00 | 2620.77 | 1037.37 | 3588.27 | 1343.39 | 3478.49 | 2467.99 | 215.96 |
| TCGA-HT-A4DS-01A-11R-A26U-07 | A | 0.23 | 0 | 1.41 | 4443.65 | 1002.29 | 824.54 | 1120.08 | 296.95 | 2736.02 | 110.74 |
| TCGA-DU-A76O-01A-11R-A32Q-07 | A | 17.40 | 0 | 34.52 | 2367.97 | 1217.35 | 970.16 | 769.07 | 1379.82 | 1911.40 | 588.65 |
| TCGA-S9-A6WG-01A-11R-A33Z-07 | A | 86.73 | 0 | 138.02 | 2757.37 | 935.66 | 728.86 | 1038.80 | 721.02 | 2190.08 | 1071.22 |
| TCGA-WH-A86K-01A-11R-A36H-07 | A | 13.50 | 0 | 54.69 | 3200.12 | 443.51 | 880.94 | 1436.90 | 752.40 | 1227.16 | 171.27 |
| TCGA-P5-A780-01A-12R-A32Q-07 | A | 2.40 | 0 | 157.30 | 3332.55 | 450.04 | 1376.24 | 1195.55 | 5550.74 | 1912.57 | 337.63 |
| TCGA-S9-A7IZ-01A-11R-A34F-07 | A | 20.27 | 0 | 167.77 | 2723.89 | 778.88 | 1244.22 | 1178.75 | 297.29 | 2510.05 | 338.15 |
| TCGA-DU-A7TJ-01A-11R-A34R-07 | A | 0.57 | 0 | 216.63 | 3470.93 | 696.01 | 97394.77 | 2263.72 | 88.34 | 3375.45 | 477.43 |
| TCGA-WY-A85A-01A-21R-A36H-07 | A | 44.00 | 0 | 52.78 | 2812.88 | 438.11 | 1238.24 | 741.62 | 1331.22 | 3625.76 | 461.34 |
| TCGA-WY-A85C-01A-11R-A36H-07 | A | 47.53 | 0 | 58.44 | 3053.53 | 514.92 | 3311.24 | 1118.51 | 62.53 | 1764.20 | 572.54 |
| TCGA-DU-6405-01A-11R-1708-07 | A | 20.17 | 1 | 265.34 | 2791.22 | 545.08 | 16967.29 | 1426.47 | 834.08 | 5250.94 | 150.15 |
| TCGA-S9-A7R8-01A-11R-A34R-07 | A | 32.03 | 1 | 110.60 | 3499.72 | 880.64 | 7836.34 | 1254.50 | 5599.55 | 2021.94 | 140.20 |
| TCGA-DU-6402-01A-11R-1708-07 | A | 7.13 | 1 | 153.98 | 3651.17 | 1059.23 | 608.85 | 1614.51 | 306.99 | 2301.31 | 225.83 |
| TCGA-DH-A66B-01A-11R-A29R-07 | A | 42.63 | 0 | 97.67 | 3666.42 | 1292.26 | 1209.62 | 1604.48 | 15.03 | 2290.68 | 60.94 |
| TCGA-S9-A89Z-01A-11R-A36H-07 | A | 20.77 | 0 | 106.70 | 3385.17 | 1469.86 | 538.26 | 1622.01 | 2466.03 | 2797.13 | 252.63 |
| TCGA-HT-A5RA-01A-11R-A28M-07 | A | 27.73 | 0 | 256.67 | 2966.82 | 933.26 | 24851.90 | 2219.30 | 4.58 | 9137.30 | 181.92 |
| TCGA-DU-A5TW-01A-11R-A28M-07 | A | 25.33 | 0 | 49.98 | 6371.68 | 410.86 | 2151.15 | 2121.54 | 1321.92 | 791.65 | 243.69 |
| TCGA-DH-A66D-01A-11R-A31N-07 | A | 28.20 | 0 | 87.96 | 2739.20 | 998.46 | 2581.01 | 1339.89 | 692.13 | 1807.48 | 398.92 |
| TCGA-DB-A4XB-01A-11R-A26U-07 | A | 30.63 | 0 | 91.02 | 2542.10 | 427.79 | 3612.34 | 1294.61 | 1349.22 | 1560.70 | 386.03 |
| TCGA-HT-A5RB-01A-11R-A28M-07 | A | 0.10 | 0 | 65.20 | 3137.10 | 232.98 | 3635.16 | 774.21 | 1452.54 | 1345.64 | 506.23 |
| TCGA-HW-A5KL-01A-11R-A27Q-07 | A | 29.27 | 0 | 74.34 | 2891.88 | 711.63 | 2856.74 | 1016.95 | 4268.85 | 1308.24 | 555.46 |
| TCGA-HT-7604-01A-11R-2090-07 | A | 124.17 | 0 | 251.25 | 3421.18 | 527.39 | 4780.26 | 1170.75 | 534.27 | 2103.68 | 595.20 |
| TCGA-DU-7010-01A-11R-2027-07 | A | 15.20 | 1 | 228.45 | 4120.25 | 537.67 | 1219.39 | 1245.93 | 8101.87 | 3860.76 | 962.63 |
| TCGA-S9-A6TS-01A-12R-A33Z-07 | A | 63.03 | 1 | 24.69 | 2496.03 | 650.79 | 793.61 | 1056.00 | 617.72 | 1517.20 | 388.89 |
| TCGA-DB-A75M-01A-11R-A32Q-07 | A | 18.13 | 0 | 25.37 | 1944.82 | 571.87 | 1559.34 | 981.31 | 2689.81 | 1152.65 | 218.07 |
| TCGA-DB-A75L-01A-11R-A32Q-07 | A | 11.40 | 0 | 105.33 | 3235.19 | 515.06 | 2843.36 | 1240.38 | 2212.25 | 1292.64 | 292.04 |
| TCGA-S9-A7QW-01A-11R-A34F-07 | A | 15.27 | 0 | 40.57 | 3470.38 | 169.20 | 1875.02 | 1789.58 | 1400.67 | 1354.05 | 312.51 |
| TCGA-TM-A7C3-01A-11R-A32Q-07 | A | 55.53 | 1 | 58.33 | 4324.79 | 355.72 | 9863.19 | 1504.20 | 71.59 | 1688.91 | 471.06 |
| TCGA-E1-A7YD-01A-11R-A34F-07 | A | 14.50 | 1 | 170.26 | 5318.96 | 442.05 | 19363.01 | 2208.00 | 27.85 | 2399.37 | 80.86 |
| TCGA-TM-A84B-01A-11R-A36H-07 | A | 25.27 | 1 | 129.30 | 3785.40 | 404.19 | 4589.20 | 1884.71 | 3.01 | 2642.15 | 217.32 |
| TCGA-FG-A4MX-01A-11R-A26U-07 | A | 18.97 | 0 | 23.09 | 3459.84 | 301.20 | 467.87 | 2356.43 | 60.24 | 664.66 | 124.50 |
| TCGA-HW-8320-01A-11R-2404-07 | A | 40.57 | 0 | 141.02 | 2736.07 | 487.10 | 4325.75 | 1016.18 | 31.14 | 1365.41 | 171.81 |
| TCGA-S9-A7QX-01A-11R-A34F-07 | A | 18.80 | 0 | 109.13 | 3179.83 | 730.27 | 2226.21 | 1119.80 | 1493.22 | 989.50 | 288.24 |
| TCGA-DH-A7UV-01A-12R-A34F-07 | A | 18.87 | 0 | 101.75 | 2740.86 | 568.60 | 1984.71 | 1317.84 | 3658.92 | 1161.59 | 193.98 |
| TCGA-WY-A85B-01A-11R-A36H-07 | A | 46.43 | 0 | 89.50 | 3875.22 | 1394.15 | 2535.25 | 844.66 | 1815.40 | 1615.32 | 229.78 |
| TCGA-TM-A7CA-01A-21R-A33Z-07 | A | 35.27 | 0 | 99.21 | 3552.09 | 377.38 | 3132.80 | 1219.00 | 3850.67 | 1742.58 | 242.57 |
| TCGA-P5-A72X-01A-11R-A32Q-07 | A | 13.43 | 0 | 41.49 | 2057.45 | 288.53 | 1683.96 | 1084.83 | 137.19 | 1335.17 | 399.80 |
| TCGA-VW-A8FI-01A-11R-A36H-07 | A | 8.17 | 1 | 63.77 | 4216.33 | 825.98 | 17710.35 | 1873.66 | 8045.89 | 4943.98 | 156.73 |
| TCGA-DU-A6S7-01A-21R-A32Q-07 | A | 21.27 | 0 | 79.61 | 2578.21 | 1308.69 | 3059.63 | 926.38 | 5001.88 | 963.85 | 158.37 |
| TCGA-CS-6666-01A-11R-1896-07 | A | 47.60 | 0 | 135.25 | 3632.72 | 610.90 | 2058.91 | 975.70 | 26.88 | 3563.64 | 313.10 |
| TCGA-HT-7477-01B-11R-A28M-07 | A | 24.60 | 0 | 256.20 | 4345.23 | 400.45 | 1584.86 | 1653.64 | 1013.15 | 5104.06 | 242.67 |
| TCGA-S9-A6U1-01A-21R-A33Z-07 | A | 26.17 | 0 | 112.79 | 2845.76 | 848.94 | 3678.25 | 785.97 | 4522.52 | 1537.66 | 1235.55 |
| TCGA-S9-A6TU-01A-12R-A32Q-07 | A | 88.33 | 0 | 76.74 | 2041.21 | 799.15 | 2661.62 | 1153.01 | 3668.88 | 1216.01 | 137.85 |
| TCGA-S9-A7IS-01A-11R-A34F-07 | A | 8.03 | 1 | 215.61 | 5149.94 | 323.88 | 108.88 | 1000.69 | 3512.20 | 5279.91 | 311.64 |
| TCGA-S9-A6WM-01A-12R-A33Z-07 | A | 20.13 | 0 | 159.98 | 5309.04 | 565.59 | 66389.93 | 2174.57 | 35.55 | 2930.31 | 116.35 |
| TCGA-P5-A5EZ-01A-11R-A27Q-07 | A | 2.33 | 0 | 82.12 | 3314.58 | 850.60 | 805.49 | 1573.99 | 7959.18 | 1998.81 | 318.03 |
| TCGA-E1-A7YL-01A-11R-A34F-07 | A | 16.40 | 1 | 92.86 | 3898.70 | 1863.01 | 4192.51 | 2186.88 | 333.46 | 2624.71 | 174.21 |
| TCGA-HT-A61B-01A-11R-A29R-07 | A | 17.77 | 0 | 60.83 | 5053.29 | 1873.89 | 259.77 | 1333.93 | 869.45 | 6212.70 | 1138.10 |
| TCGA-TM-A84I-01A-11R-A36H-07 | A | 28.47 | 0 | 209.11 | 3766.74 | 534.27 | 3672.85 | 1001.95 | 274.14 | 1956.78 | 146.03 |
| TCGA-DB-A4XD-01A-11R-A27Q-07 | A | 40.33 | 0 | 95.12 | 2649.62 | 550.19 | 5163.89 | 1273.90 | 3416.83 | 1161.09 | 446.94 |
| TCGA-DU-7298-01A-11R-2027-07 | A | 19.20 | 1 | 172.90 | 3587.43 | 764.20 | 5106.76 | 1017.86 | 5195.13 | 2100.44 | 448.72 |
| TCGA-P5-A735-01A-11R-A32Q-07 | A | 9.73 | 0 | 32.75 | 2422.58 | 450.97 | 1834.88 | 950.76 | 3742.16 | 788.76 | 193.03 |
| TCGA-RY-A83Z-01A-11R-A36H-07 | A | 10.03 | 0 | 3.51 | 4762.77 | 383.89 | 214.78 | 1563.17 | 526.91 | 944.93 | 51.19 |
| TCGA-CS-4943-01A-01R-1470-07 | A | 36.87 | 1 | 225.32 | 3770.07 | 813.23 | 6477.45 | 1127.24 | 1662.00 | 1776.45 | 178.69 |
| TCGA-FG-8185-01A-11R-2256-07 | A | 14.43 | 0 | 73.04 | 3497.14 | 201.38 | 1620.07 | 880.67 | 34.57 | 1868.65 | 126.54 |
| TCGA-TM-A7CF-01A-11R-A32Q-07 | A | 66.30 | 0 | 23.18 | 2077.77 | 221.91 | 3293.19 | 884.87 | 1158.67 | 1449.03 | 483.01 |
| TCGA-KT-A7W1-01A-11R-A34F-07 | A | 14.60 | 0 | 238.89 | 4404.74 | 334.32 | 30132.10 | 2077.00 | 33.56 | 10808.82 | 324.12 |
| TCGA-DH-A7UU-01A-12R-A34F-07 | A | 13.90 | 0 | 232.07 | 2874.84 | 398.96 | 3527.06 | 846.15 | 2443.72 | 1196.87 | 333.33 |
| TCGA-DH-5142-01A-01R-1470-07 | A | 64.77 | 0 | 84.43 | 3291.24 | 1840.38 | 3135.42 | 1183.44 | 184.80 | 3089.77 | 363.08 |
| TCGA-E1-A7YI-01A-11R-A34F-07 | A | 3.70 | 1 | 294.41 | 4600.40 | 444.59 | 136.28 | 1173.01 | 1138.93 | 2958.32 | 108.83 |
| TCGA-HT-8011-01A-11R-2403-07 | A | 16.47 | 0 | 245.48 | 8111.78 | 507.55 | 20832.79 | 2980.82 | 279.30 | 2703.41 | 232.02 |
| TCGA-HT-A74O-01A-11R-A32Q-07 | A | 0.10 | 0 | 35.84 | 2620.00 | 1599.33 | 324.93 | 1455.02 | 1665.75 | 2934.42 | 191.61 |
| TCGA-VV-A86M-01A-11R-A36H-07 | A | 16.23 | 0 | 130.02 | 4890.63 | 887.83 | 1318.05 | 1361.05 | 8.37 | 2061.94 | 95.98 |
| TCGA-TM-A84Q-01A-11R-A36H-07 | A | 25.73 | 0 | 92.11 | 3207.51 | 873.48 | 2731.61 | 1101.49 | 2382.00 | 2838.03 | 164.41 |
| TCGA-E1-5304-01A-01R-1470-07 | A | 41.70 | 1 | 298.76 | 4124.57 | 162.22 | 3781.81 | 937.80 | 1145.07 | 1490.63 | 71.08 |
| TCGA-27-1831-01A-01R-1850-01 | GBM | 16.83 | 1 | 44.56 | 13174.33 | 2903.24 | 4022.79 | 5026.61 | 122.14 | 6380.85 | 229.42 |
| TCGA-14-1829-01A-01R-1850-01 | GBM | 7.27 | 0 | 110.89 | 11871.60 | 4263.36 | 6157.18 | 3363.85 | 1836.95 | 2987.42 | 285.97 |
| TCGA-14-0736-02A-01R-2005-01 | GBM | 15.33 | 1 | 25.93 | 12898.53 | 5134.16 | 3191.63 | 1600.90 | 1606.54 | 2678.69 | 236.75 |
| TCGA-14-1823-01A-01R-1849-01 | GBM | 18.10 | 1 | 90.77 | 6241.56 | 3039.01 | 5279.80 | 1846.21 | 8.25 | 3651.16 | 243.81 |
| TCGA-06-0132-01A-02R-1849-01 | GBM | 25.70 | 1 | 48.85 | 2320.87 | 4910.13 | 8928.12 | 932.45 | 6566.95 | 3483.72 | 592.88 |
| TCGA-28-5213-01A-01R-1850-01 | GBM | 31.70 | 0 | 89.92 | 5976.84 | 5420.98 | 941.39 | 1841.96 | 524.52 | 2784.74 | 509.54 |
| TCGA-06-0219-01A-01R-1849-01 | GBM | 0.73 | 1 | 120.70 | 5505.79 | 2235.20 | 3797.72 | 2066.87 | 3650.30 | 4630.24 | 269.45 |
| TCGA-02-2486-01A-01R-1849-01 | GBM | 20.60 | 1 | 119.51 | 4196.95 | 4528.66 | 6124.96 | 2330.49 | 1419.51 | 14254.88 | 209.15 |
| TCGA-32-1980-01A-01R-1850-01 | GBM | 1.20 | 1 | 43.50 | 2003.35 | 2849.04 | 902.99 | 999.55 | 3422.56 | 3596.58 | 749.66 |
| TCGA-06-0141-01A-01R-1849-01 | GBM | 10.43 | 1 | 64.17 | 5699.18 | 6232.55 | 743.32 | 2714.07 | 648.36 | 3537.99 | 293.12 |
| TCGA-27-2526-01A-01R-1850-01 | GBM | 2.90 | 1 | 56.18 | 5573.03 | 1638.76 | 23745.98 | 1938.20 | 115.17 | 4701.12 | 94.38 |
| TCGA-06-5410-01A-01R-1849-01 | GBM | 3.60 | 1 | 59.32 | 3002.30 | 10626.44 | 303.22 | 1237.02 | 5081.05 | 3526.46 | 330.86 |
| TCGA-06-0171-02A-11R-2005-01 | GBM | 13.30 | 1 | 111.80 | 1796.56 | 10627.72 | 1153.70 | 882.02 | 1538.14 | 7211.00 | 429.32 |
| TCGA-32-1982-01A-01R-1850-01 | GBM | 4.73 | 1 | 162.23 | 4243.35 | 1940.01 | 10607.30 | 1470.22 | 294.04 | 2516.27 | 359.95 |
| TCGA-12-5295-01A-01R-1849-01 | GBM | 15.13 | 1 | 144.24 | 4724.40 | 4057.31 | 7750.65 | 1028.98 | 37.35 | 2660.66 | 177.72 |
| TCGA-14-2554-01A-01R-1850-01 | GBM | 17.73 | 1 | 113.31 | 5089.20 | 1891.03 | 32453.36 | 2031.34 | 84.86 | 2512.05 | 184.19 |
| TCGA-06-5412-01A-01R-1849-01 | GBM | 4.60 | 1 | 101.22 | 4339.16 | 3400.64 | 372.53 | 1755.17 | 162.16 | 4947.54 | 318.50 |
| TCGA-14-1402-02A-01R-2005-01 | GBM | 32.50 | 1 | 159.69 | 6372.60 | 1588.36 | 9953.10 | 2340.67 | 408.09 | 6763.66 | 124.20 |
| TCGA-06-0749-01A-01R-1849-01 | GBM | 2.73 | 1 | 10.81 | 2955.63 | 2167.24 | 553.46 | 1672.92 | 2408.42 | 2464.73 | 216.72 |
| TCGA-06-5411-01A-01R-1849-01 | GBM | 8.47 | 1 | 74.59 | 5691.53 | 1693.37 | 293.72 | 2010.59 | 5371.55 | 2408.38 | 593.46 |
| TCGA-19-4065-01A-01R-2005-01 | GBM | 7.13 | 0 | 209.53 | 6762.80 | 3676.45 | 401.95 | 2805.08 | 514.55 | 5316.07 | 241.83 |
| TCGA-27-1832-01A-01R-1850-01 | GBM | 10.00 | 1 | 106.13 | 10132.55 | 2525.91 | 732.51 | 2646.95 | 7.68 | 4260.58 | 826.46 |
| TCGA-06-0138-01A-02R-1849-01 | GBM | 24.57 | 1 | 71.87 | 6127.16 | 2915.69 | 2074.58 | 1806.50 | 1554.94 | 2843.81 | 266.07 |
| TCGA-14-0781-01B-01R-1849-01 | GBM | 0.97 | 1 | 70.90 | 3270.70 | 2011.40 | 330.67 | 1560.73 | 58.50 | 3742.69 | 254.83 |
| TCGA-19-1787-01B-01R-1850-01 | GBM | 12.83 | 1 | 301.03 | 4118.43 | 1306.20 | 2001.21 | 2292.85 | 2083.12 | 5914.73 | 207.15 |
| TCGA-06-2561-01A-02R-1849-01 | GBM | 17.90 | 1 | 113.80 | 3554.93 | 2236.62 | 1734.05 | 1971.83 | 1298.59 | 4092.96 | 227.61 |
| TCGA-76-4929-01A-01R-1850-01 | GBM | 3.70 | 1 | 98.27 | 2914.01 | 1233.39 | 3492.95 | 1114.46 | 861.53 | 2560.02 | 223.34 |
| TCGA-19-0957-02A-11R-2005-01 | GBM | 22.20 | 1 | 137.40 | 2832.06 | 1327.15 | 881.12 | 1331.52 | 10149.95 | 4552.34 | 398.58 |
| TCGA-27-1834-01A-01R-1850-01 | GBM | 41.10 | 1 | 143.49 | 3908.03 | 1895.29 | 1401.08 | 3625.48 | 185.04 | 11561.22 | 278.67 |
| TCGA-02-0055-01A-01R-1849-01 | GBM | 2.53 | 1 | 85.77 | 4214.72 | 2837.84 | 264.05 | 1684.65 | 53.82 | 3910.86 | 509.60 |
| TCGA-26-5136-01B-01R-1850-01 | GBM | 19.23 | 1 | 95.02 | 6289.59 | 1751.13 | 3104.03 | 2980.39 | 714.93 | 4907.99 | 315.23 |
| TCGA-06-0649-01B-01R-1849-01 | GBM | 2.13 | 1 | 75.14 | 3558.87 | 3192.87 | 1211.96 | 1670.61 | 34.33 | 1888.91 | 298.62 |
| TCGA-06-0238-01A-02R-1849-01 | GBM | 13.50 | 1 | 62.28 | 7452.71 | 2779.70 | 143.00 | 2122.26 | 2904.27 | 6021.34 | 587.66 |
| TCGA-06-2567-01A-01R-1849-01 | GBM | 4.43 | 1 | 368.50 | 3900.12 | 1383.91 | 4575.42 | 1387.74 | 608.22 | 6186.38 | 858.57 |
| TCGA-06-5859-01A-01R-1849-01 | GBM | 4.63 | 0 | 429.64 | 4418.50 | 2801.11 | 6551.50 | 2863.06 | 42.79 | 4200.11 | 106.52 |
| TCGA-16-1045-01B-01R-1850-01 | GBM | 29.43 | 1 | 149.53 | 4216.20 | 4470.41 | 25341.12 | 1560.75 | 793.77 | 4031.78 | 412.46 |
| TCGA-28-5207-01A-01R-1850-01 | GBM | 11.43 | 1 | 95.05 | 5859.86 | 1389.50 | 8721.31 | 1549.89 | 395.84 | 2269.20 | 492.51 |
| TCGA-41-4097-01A-01R-1850-01 | GBM | 0.20 | 1 | 97.47 | 1812.74 | 2118.96 | 3178.73 | 815.04 | 672.29 | 3349.19 | 275.52 |
| TCGA-28-5215-01A-01R-1850-01 | GBM | 11.17 | 1 | 151.24 | 2994.23 | 1493.45 | 996.60 | 1218.98 | 917.36 | 3597.04 | 1150.90 |
| TCGA-06-0178-01A-01R-1849-01 | GBM | 89.37 | 1 | 125.31 | 4713.43 | 2603.88 | 457.41 | 947.15 | 1163.94 | 2058.81 | 357.49 |
| TCGA-06-0882-01A-01R-1849-01 | GBM | 21.07 | 1 | 91.05 | 2923.38 | 2229.26 | 877.11 | 1623.37 | 2637.57 | 4811.92 | 265.63 |
| TCGA-06-2562-01A-01R-1849-01 | GBM | 12.73 | 1 | 88.67 | 4086.43 | 1092.70 | 438.41 | 1829.38 | 152.26 | 2733.99 | 425.88 |
| TCGA-76-4926-01B-01R-1850-01 | GBM | 4.60 | 1 | 126.65 | 3721.75 | 746.23 | 6927.82 | 2155.37 | 580.51 | 3658.19 | 150.66 |
| TCGA-76-4928-01B-01R-1850-01 | GBM | 3.13 | 1 | 86.33 | 3867.22 | 1312.88 | 322.52 | 1008.33 | 550.83 | 2389.57 | 180.54 |
| TCGA-06-5856-01A-01R-1849-01 | GBM | 3.80 | 1 | 132.37 | 5887.78 | 1536.92 | 20246.88 | 1725.61 | 187.72 | 2543.89 | 483.27 |
| TCGA-06-0750-01A-01R-1849-01 | GBM | 0.93 | 1 | 20.19 | 4632.93 | 1819.12 | 5167.93 | 2137.77 | 167.51 | 4159.32 | 265.72 |
| TCGA-28-5216-01A-01R-1850-01 | GBM | 13.83 | 0 | 117.85 | 3016.76 | 866.81 | 383.54 | 1309.63 | 112.27 | 3163.20 | 691.78 |
| TCGA-12-0616-01A-01R-1849-01 | GBM | 14.93 | 1 | 112.86 | 5665.85 | 1403.52 | 1581.68 | 1333.94 | 5.94 | 4954.75 | 219.78 |
| TCGA-06-0644-01A-02R-1849-01 | GBM | 12.80 | 1 | 112.36 | 4489.02 | 2867.63 | 544.64 | 1852.16 | 206.16 | 3040.82 | 605.47 |
| TCGA-06-0168-01A-01R-1849-01 | GBM | 19.93 | 1 | 110.09 | 2651.38 | 1791.28 | 1116.97 | 910.55 | 802.75 | 3183.49 | 479.36 |
| TCGA-06-1804-01A-01R-1849-01 | GBM | 13.80 | 1 | 167.22 | 2731.77 | 766.56 | 4149.56 | 2096.51 | 221.12 | 4088.91 | 160.31 |
| TCGA-02-0047-01A-01R-1849-01 | GBM | 14.93 | 1 | 192.38 | 2086.73 | 1692.74 | 564.24 | 875.26 | 387.84 | 3086.76 | 1035.68 |
| TCGA-19-1389-02A-21R-2005-01 | GBM | 4.70 | 1 | 103.89 | 4539.94 | 3724.49 | 591.33 | 2092.99 | 3.02 | 3838.66 | 296.58 |
| TCGA-14-0817-01A-01R-1849-01 | GBM | 5.47 | 1 | 170.09 | 4637.85 | 3163.71 | 5874.28 | 2562.72 | 687.17 | 6791.21 | 417.86 |
| TCGA-28-5220-01A-01R-1850-01 | GBM | 12.93 | 1 | 110.92 | 6255.57 | 2635.79 | 4945.05 | 1618.64 | 667.24 | 6842.77 | 201.26 |
| TCGA-06-0646-01A-01R-1849-01 | GBM | 5.83 | 1 | 190.15 | 3456.40 | 2052.57 | 5309.65 | 1400.32 | 260.90 | 3905.95 | 255.38 |
| TCGA-27-2519-01A-01R-1850-01 | GBM | 18.33 | 1 | 69.97 | 4713.13 | 2720.13 | 429.49 | 2141.01 | 31.75 | 3275.57 | 431.11 |
| TCGA-06-5417-01A-01R-1849-01 | GBM | 5.17 | 0 | 148.86 | 4368.32 | 708.02 | 3001.91 | 1580.15 | 19.08 | 2566.79 | 633.59 |
| TCGA-27-1835-01A-01R-1850-01 | GBM | 21.60 | 1 | 34.83 | 3304.09 | 393.84 | 528.46 | 1859.34 | 8.04 | 2926.99 | 191.56 |
| TCGA-14-1825-01A-01R-1850-01 | GBM | 7.73 | 1 | 140.47 | 4916.39 | 488.29 | 1530.03 | 1571.91 | 284.28 | 2168.90 | 847.83 |
| TCGA-15-1444-01A-02R-1850-01 | GBM | 51.23 | 1 | 127.53 | 2445.25 | 1751.69 | 2924.18 | 1086.07 | 7776.03 | 7390.27 | 493.47 |
| TCGA-28-2513-01A-01R-1850-01 | GBM | 7.40 | 0 | 165.30 | 3648.52 | 3572.73 | 28039.46 | 2076.09 | 40.58 | 6314.19 | 374.76 |
| TCGA-06-5418-01A-01R-1849-01 | GBM | 2.77 | 1 | 199.51 | 7154.00 | 1820.88 | 1449.16 | 3557.40 | 38.71 | 2046.20 | 93.30 |
| TCGA-06-0184-01A-01R-1849-01 | GBM | 70.87 | 1 | 165.77 | 5164.30 | 3184.89 | 1158.89 | 2743.99 | 1862.68 | 3702.31 | 574.30 |
| TCGA-28-2514-01A-02R-1850-01 | GBM | 5.33 | 0 | 193.51 | 2697.56 | 760.20 | 75934.59 | 1245.35 | 235.89 | 7428.30 | 233.13 |
| TCGA-19-2620-01A-01R-1850-01 | GBM | 4.93 | 1 | 135.53 | 2432.23 | 738.10 | 14317.59 | 1429.49 | 6694.14 | 2423.08 | 195.97 |
| TCGA-12-1597-01B-01R-1849-01 | GBM | 22.50 | 1 | 130.49 | 4112.91 | 1152.13 | 6642.23 | 1374.58 | 4156.86 | 2281.95 | 323.87 |
| TCGA-06-5413-01A-01R-1849-01 | GBM | 8.93 | 0 | 198.02 | 7950.61 | 3829.26 | 25486.64 | 1774.22 | 138.29 | 3235.65 | 760.11 |
| TCGA-32-5222-01A-01R-1850-01 | GBM | 19.50 | 1 | 183.51 | 8269.98 | 2565.61 | 20390.27 | 2197.59 | 1912.52 | 4745.60 | 244.85 |
| TCGA-06-0130-01A-01R-1849-01 | GBM | 13.13 | 1 | 135.12 | 6496.65 | 7721.51 | 349.12 | 1500.77 | 150.59 | 9176.90 | 4451.26 |
| TCGA-32-4213-01A-01R-1850-01 | GBM | 20.13 | 0 | 226.57 | 3037.76 | 3391.61 | 6550.50 | 1260.14 | 4.20 | 6959.44 | 441.96 |
| TCGA-26-5134-01A-01R-1850-01 | GBM | 5.57 | 0 | 198.77 | 3831.97 | 911.89 | 659.84 | 918.03 | 1377.05 | 3247.95 | 696.72 |
| TCGA-27-1830-01A-01R-1850-01 | GBM | 5.13 | 1 | 173.44 | 4140.10 | 1236.46 | 903.62 | 2621.35 | 150.52 | 3923.44 | 478.65 |
| TCGA-06-0878-01A-01R-1849-01 | GBM | 7.27 | 0 | 133.55 | 4531.17 | 1959.80 | 19351.59 | 2096.53 | 87.97 | 3393.87 | 261.80 |
| TCGA-06-5858-01A-01R-1849-01 | GBM | 6.23 | 0 | 206.47 | 4411.58 | 1356.43 | 1922.49 | 2816.77 | 2961.71 | 3839.11 | 371.01 |
| TCGA-06-2558-01A-01R-1849-01 | GBM | 12.67 | 1 | 174.75 | 5132.83 | 1567.68 | 1651.51 | 2033.84 | 1118.18 | 2561.62 | 408.59 |
| TCGA-06-0156-01A-03R-1849-01 | GBM | 5.93 | 1 | 282.98 | 5729.03 | 2668.67 | 1219.83 | 2389.51 | 31.69 | 2065.01 | 829.28 |
| TCGA-12-5299-01A-02R-1849-01 | GBM | 3.27 | 1 | 205.73 | 7043.63 | 1699.74 | 49813.82 | 1937.13 | 58.60 | 5912.32 | 123.61 |
| TCGA-28-2509-01A-01R-1850-01 | GBM | 4.83 | 0 | 255.01 | 4392.28 | 1022.04 | 1012.02 | 1337.68 | 19.54 | 2165.83 | 41.08 |
| TCGA-06-5416-01A-01R-1849-01 | GBM | 6.80 | 0 | 123.60 | 4868.95 | 274.76 | 265.79 | 1451.23 | 95.31 | 2521.97 | 209.98 |
| TCGA-12-0618-01A-01R-1849-01 | GBM | 13.17 | 1 | 176.75 | 4628.83 | 632.36 | 4209.61 | 2362.53 | 361.35 | 2199.14 | 486.65 |
| TCGA-06-0210-01A-01R-1849-01 | GBM | 7.50 | 1 | 129.72 | 4220.72 | 2028.56 | 906.06 | 2036.30 | 703.78 | 2399.81 | 105.03 |
| TCGA-12-3652-01A-01R-1849-01 | GBM | 35.40 | 1 | 122.11 | 4573.26 | 403.60 | 10816.68 | 1361.18 | 12.21 | 4292.42 | 35.99 |
| TCGA-28-1747-01C-01R-1850-01 | GBM | 2.57 | 1 | 118.72 | 3070.23 | 2050.08 | 10470.38 | 2106.27 | 2151.70 | 12066.94 | 187.13 |
| TCGA-32-2615-01A-01R-1850-01 | GBM | 16.17 | 1 | 136.07 | 4083.37 | 1336.07 | 50675.69 | 1372.35 | 1484.67 | 4245.79 | 206.05 |
| TCGA-26-1442-01A-01R-1850-01 | GBM | 31.77 | 0 | 144.73 | 2561.09 | 520.71 | 1399.02 | 1324.59 | 1067.12 | 2719.98 | 434.71 |
| TCGA-26-5132-01A-01R-1850-01 | GBM | 9.53 | 0 | 277.87 | 4205.74 | 650.61 | 14429.40 | 3425.39 | 197.99 | 5097.42 | 271.09 |
| TCGA-14-1034-01A-01R-1849-01 | GBM | 16.17 | 1 | 210.05 | 2969.07 | 1793.27 | 1316.42 | 1307.98 | 3.02 | 3303.46 | 97.78 |
| TCGA-27-1837-01A-01R-1850-01 | GBM | 14.23 | 1 | 109.28 | 2205.64 | 1121.03 | 15511.48 | 1756.76 | 340.78 | 11782.61 | 580.49 |
| TCGA-06-0745-01A-01R-1849-01 | GBM | 7.97 | 1 | 71.70 | 5873.57 | 1476.88 | 8360.34 | 1514.21 | 103.10 | 1537.55 | 231.65 |
| TCGA-06-0645-01A-01R-1849-01 | GBM | 5.83 | 1 | 105.07 | 3357.03 | 3970.20 | 10123.18 | 1854.68 | 1112.91 | 3627.29 | 532.67 |
| TCGA-16-0846-01A-01R-1850-01 | GBM | 3.97 | 1 | 67.05 | 2975.36 | 1853.87 | 290.52 | 1673.93 | 3043.55 | 18434.96 | 261.89 |
| TCGA-14-0789-01A-01R-1849-01 | GBM | 11.40 | 1 | 126.34 | 2385.98 | 2227.42 | 16095.79 | 1360.08 | 42.32 | 4537.59 | 203.41 |
| TCGA-26-5135-01A-01R-1850-01 | GBM | 9.00 | 1 | 75.32 | 2794.94 | 787.97 | 389.86 | 967.09 | 1479.11 | 2927.22 | 400.63 |
| TCGA-06-0187-01A-01R-1849-01 | GBM | 27.60 | 1 | 254.93 | 2253.56 | 1163.30 | 86384.59 | 1730.09 | 15.11 | 9028.84 | 150.64 |
| TCGA-41-3915-01A-01R-1850-01 | GBM | 12.00 | 1 | 244.40 | 2906.46 | 2402.50 | 451.25 | 1361.66 | 237.15 | 2485.51 | 417.00 |
| TCGA-41-2571-01A-01R-1850-01 | GBM | 0.87 | 1 | 77.72 | 3986.82 | 613.54 | 806.04 | 2263.38 | 5033.72 | 1386.06 | 128.95 |
| TCGA-27-2528-01A-01R-1850-01 | GBM | 16.00 | 1 | 188.96 | 5136.60 | 400.29 | 15776.70 | 968.59 | 7.62 | 2691.10 | 605.90 |
| TCGA-12-0619-01A-01R-1849-01 | GBM | 35.40 | 1 | 290.93 | 3394.44 | 2313.73 | 26544.70 | 2388.51 | 5.02 | 4930.23 | 220.25 |
| TCGA-14-0787-01A-01R-1849-01 | GBM | 2.27 | 1 | 97.97 | 4613.21 | 1211.18 | 9595.94 | 1700.29 | 53.70 | 2536.28 | 168.36 |
| TCGA-76-4932-01A-01R-1850-01 | GBM | 48.60 | 1 | 124.38 | 4270.15 | 620.90 | 9059.57 | 1833.83 | 7.46 | 2576.12 | 141.79 |
| TCGA-32-2632-01A-01R-1850-01 | GBM | 8.97 | 1 | 180.71 | 3557.34 | 3140.65 | 34062.80 | 1896.66 | 30.95 | 4906.33 | 139.27 |
| TCGA-06-2559-01A-01R-1849-01 | GBM | 5.00 | 1 | 255.51 | 4667.89 | 1005.77 | 2397.65 | 2918.15 | 262.33 | 3495.28 | 247.64 |
| TCGA-19-2624-01A-01R-1850-01 | GBM | 0.17 | 1 | 158.43 | 5165.14 | 309.44 | 13508.70 | 1765.03 | 74.27 | 2262.20 | 131.20 |
| TCGA-28-5204-01A-01R-1850-01 | GBM | 15.13 | 1 | 235.44 | 7319.62 | 915.39 | 18799.90 | 1812.36 | 6.47 | 11687.49 | 252.37 |
| TCGA-06-5408-01A-01R-1849-01 | GBM | 11.90 | 1 | 172.28 | 5224.52 | 749.20 | 10488.91 | 1847.38 | 5.30 | 8994.37 | 136.50 |
| TCGA-32-2638-01A-01R-1850-01 | GBM | 25.53 | 1 | 209.91 | 2812.89 | 2000.79 | 50665.70 | 926.10 | 62.89 | 12561.71 | 300.31 |
| TCGA-12-3653-01A-01R-1849-01 | GBM | 14.73 | 1 | 390.14 | 4063.09 | 1274.11 | 147457.43 | 1000.73 | 31.18 | 28640.32 | 92.10 |
| TCGA-06-0190-01A-01R-1849-01 | GBM | 10.57 | 1 | 163.20 | 3655.41 | 2708.75 | 471.95 | 2410.48 | 78.84 | 3238.59 | 768.02 |
| TCGA-06-5414-01A-01R-1849-01 | GBM | 9.10 | 0 | 192.62 | 4055.71 | 1882.70 | 47190.54 | 1824.24 | 191.45 | 6626.91 | 217.34 |
| TCGA-06-5415-01A-01R-1849-01 | GBM | 8.67 | 0 | 225.74 | 4826.48 | 791.10 | 41114.57 | 1314.78 | 396.40 | 3658.39 | 417.81 |
| TCGA-06-2564-01A-01R-1849-01 | GBM | 6.03 | 0 | 198.03 | 4892.43 | 1423.44 | 18300.64 | 2647.65 | 1060.98 | 2545.23 | 254.44 |
| TCGA-14-0790-01B-01R-1849-01 | GBM | 13.97 | 1 | 95.56 | 4058.02 | 680.55 | 25554.07 | 2049.15 | 43.00 | 7980.20 | 348.12 |
| TCGA-06-0158-01A-01R-1849-01 | GBM | 10.97 | 1 | 199.93 | 2070.24 | 1444.46 | 21442.28 | 1089.07 | 1526.81 | 7980.18 | 297.70 |
| TCGA-76-4931-01A-01R-1850-01 | GBM | 9.30 | 1 | 167.06 | 4284.24 | 490.35 | 12877.32 | 1980.71 | 121.88 | 2281.88 | 710.12 |
| TCGA-76-4925-01A-01R-1850-01 | GBM | 4.87 | 1 | 253.75 | 4483.07 | 557.22 | 1684.93 | 2409.34 | 198.89 | 2074.15 | 122.59 |
| TCGA-26-5139-01A-01R-1850-01 | GBM | 1.60 | 0 | 145.07 | 3518.00 | 1156.61 | 12048.22 | 2208.95 | 450.52 | 3300.39 | 222.14 |
| TCGA-76-4927-01A-01R-1850-01 | GBM | 17.83 | 1 | 114.86 | 3594.38 | 1337.35 | 11039.04 | 1982.33 | 3142.97 | 3093.98 | 548.59 |
| TCGA-19-2625-01A-01R-1850-01 | GBM | 4.13 | 1 | 138.80 | 3049.43 | 453.78 | 517.28 | 1918.06 | 62.48 | 2737.52 | 70.17 |
| TCGA-06-2557-01A-01R-1849-01 | GBM | 1.10 | 1 | 223.86 | 2914.34 | 4296.54 | 23491.66 | 1255.11 | 2472.85 | 9593.19 | 213.89 |
| TCGA-06-0743-01A-01R-1849-01 | GBM | 26.77 | 1 | 30.21 | 3352.94 | 975.09 | 11606.17 | 1823.00 | 88.50 | 2304.72 | 181.77 |
| TCGA-32-1970-01A-01R-1850-01 | GBM | 15.60 | 1 | 238.00 | 4305.58 | 806.56 | 42979.91 | 2213.52 | 70.03 | 6043.58 | 135.16 |
| TCGA-06-0747-01A-01R-1849-01 | GBM | 2.73 | 1 | 164.27 | 2383.66 | 481.00 | 36708.89 | 1435.73 | 28.13 | 5227.91 | 51.28 |
| TCGA-08-0386-01A-01R-1849-01 | GBM | 18.27 | 1 | 338.09 | 3521.19 | 465.56 | 9228.81 | 1395.77 | 127.01 | 2949.17 | 121.53 |
| TCGA-28-5218-01A-01R-1850-01 | GBM | 5.23 | 1 | 61.87 | 5767.04 | 2957.31 | 726.91 | 739.17 | 39.95 | 2365.72 | 285.48 |
| TCGA-32-2616-01A-01R-1850-01 | GBM | 7.47 | 1 | 203.10 | 4594.67 | 1060.00 | 1821.58 | 3107.51 | 622.53 | 6756.24 | 87.57 |
| TCGA-41-2572-01A-01R-1850-01 | GBM | 13.53 | 1 | 95.19 | 3548.81 | 988.49 | 43650.52 | 2071.57 | 499.74 | 8584.03 | 176.34 |
| TCGA-28-5208-01A-01R-1850-01 | GBM | 18.13 | 1 | 158.05 | 7213.60 | 1530.65 | 5238.42 | 1467.91 | 111.59 | 3550.29 | 286.40 |
| TCGA-06-0686-01A-01R-1849-01 | GBM | 14.40 | 1 | 85.46 | 5045.51 | 875.69 | 528.25 | 1891.23 | 1594.89 | 2071.03 | 289.68 |
| TCGA-12-0821-01A-01R-1849-01 | GBM | 10.77 | 1 | 157.54 | 6499.82 | 627.32 | 1151.98 | 2847.55 | 538.85 | 2380.60 | 200.12 |
| TCGA-06-0129-01A-01R-1849-01 | GBM | 34.13 | 1 | 304.50 | 3409.70 | 2354.03 | 6031.37 | 837.69 | 1138.08 | 13603.36 | 364.25 |
| TCGA-06-0157-01A-01R-1849-01 | GBM | 3.23 | 1 | 124.71 | 3121.21 | 1442.42 | 14668.19 | 1283.87 | 44.95 | 4420.06 | 492.08 |
| TCGA-28-1753-01A-01R-1850-01 | GBM | 1.23 | 0 | 157.56 | 3885.32 | 6012.86 | 9251.65 | 1344.05 | 272.24 | 3547.70 | 680.60 |
| TCGA-06-2569-01A-01R-1849-01 | GBM | 0.43 | 0 | 287.91 | 3438.35 | 167.55 | 1643.86 | 736.28 | 16.52 | 929.79 | 92.04 |
| TCGA-26-5133-01A-01R-1850-01 | GBM | 15.07 | 0 | 177.58 | 3005.77 | 392.78 | 457.49 | 2938.55 | 20.57 | 2310.01 | 1271.63 |
| TCGA-19-2619-01A-01R-1850-01 | GBM | 9.80 | 0 | 242.94 | 2857.97 | 546.93 | 2819.71 | 804.40 | 21.18 | 2144.10 | 213.04 |
| TCGA-06-2563-01A-01R-1849-01 | GBM | 31.07 | 0 | 195.40 | 4262.89 | 1062.42 | 33026.61 | 1631.15 | 348.32 | 7333.10 | 479.53 |
| TCGA-12-3650-01A-01R-1849-01 | GBM | 11.10 | 1 | 222.60 | 4968.32 | 1107.88 | 23120.29 | 1676.37 | 556.51 | 2583.90 | 208.90 |
| TCGA-15-0742-01A-01R-1850-01 | GBM | 13.97 | 1 | 72.63 | 5156.32 | 340.00 | 10908.80 | 1372.63 | 13.16 | 3906.32 | 106.84 |
| TCGA-27-2521-01A-01R-1850-01 | GBM | 17.00 | 1 | 204.87 | 3418.88 | 426.48 | 1917.77 | 1835.89 | 534.42 | 1157.40 | 142.31 |
| TCGA-06-2565-01A-01R-1849-01 | GBM | 16.87 | 1 | 184.10 | 2826.53 | 1269.98 | 55872.98 | 1095.66 | 25.51 | 6060.80 | 265.31 |
| TCGA-06-0174-01A-01R-1849-01 | GBM | 3.27 | 1 | 220.37 | 4774.58 | 671.21 | 945.54 | 1557.24 | 283.41 | 3051.94 | 237.01 |
| TCGA-06-0211-01B-01R-1849-01 | GBM | 12.00 | 1 | 280.78 | 3537.56 | 977.75 | 15905.45 | 1497.38 | 74.29 | 2362.50 | 239.65 |
| TCGA-06-0152-02A-01R-2005-01 | GBM | 12.50 | 1 | 207.88 | 3571.18 | 2302.45 | 18165.79 | 1536.13 | 63.90 | 10089.45 | 539.18 |
| TCGA-19-5960-01A-11R-1850-01 | GBM | 15.17 | 1 | 276.48 | 7606.60 | 422.28 | 19694.01 | 1666.21 | 762.95 | 3137.55 | 219.62 |
| TCGA-27-2523-01A-01R-1850-01 | GBM | 16.30 | 1 | 175.02 | 4717.01 | 540.81 | 3460.61 | 1784.07 | 119.57 | 3203.15 | 185.64 |
| TCGA-27-2524-01A-01R-1850-01 | GBM | 7.70 | 1 | 68.27 | 5590.06 | 1852.97 | 281.48 | 2067.97 | 177.87 | 1901.48 | 164.10 |
| TCGA-41-5651-01A-01R-1850-01 | GBM | 15.33 | 1 | 136.52 | 6517.75 | 341.92 | 1615.10 | 1560.26 | 1663.87 | 1673.02 | 487.58 |
| TCGA-02-2483-01A-01R-1849-01 | GBM | 15.53 | 0 | 100.05 | 6578.58 | 1401.58 | 190.81 | 1541.32 | 16.04 | 3437.68 | 263.85 |
| TCGA-06-0125-01A-01R-1849-01 | GBM | 48.27 | 1 | 188.23 | 4202.49 | 568.34 | 29987.21 | 1452.03 | 13.44 | 2216.34 | 264.41 |
| TCGA-02-2485-01A-01R-1849-01 | GBM | 15.67 | 0 | 134.76 | 4065.24 | 669.05 | 27073.10 | 1713.81 | 112.38 | 8580.00 | 131.90 |
| TCGA-06-0221-02A-11R-2005-01 | GBM | 20.10 | 1 | 200.50 | 2643.61 | 683.21 | 3426.02 | 1288.22 | 2521.30 | 3105.26 | 839.10 |
| TCGA-19-1390-01A-01R-1850-01 | GBM | 25.73 | 1 | 186.02 | 4455.07 | 538.52 | 222.24 | 925.61 | 3352.40 | 2546.45 | 100.13 |
| TCGA-06-2570-01A-01R-1849-01 | GBM | 31.93 | 0 | 168.06 | 2790.04 | 1337.22 | 725.18 | 819.43 | 843.72 | 2718.15 | 379.84 |
| TCGA-32-2634-01A-01R-1850-01 | GBM | 23.10 | 0 | 288.78 | 3447.90 | 870.90 | 959.04 | 1561.70 | 1376.65 | 2728.22 | 355.66 |
| TCGA-28-5209-01A-01R-1850-01 | GBM | 14.73 | 0 | 238.65 | 5146.93 | 633.57 | 76941.07 | 1373.55 | 4.45 | 7045.41 | 362.42 |
| TCGA-06-0744-01A-01R-1849-01 | GBM | 47.53 | 1 | 80.47 | 5047.17 | 699.96 | 39274.66 | 2065.32 | 472.04 | 3108.78 | 189.03 |
| TCGA-19-2629-01A-01R-1850-01 | GBM | 24.57 | 1 | 128.62 | 3129.24 | 1401.46 | 1374.38 | 1299.48 | 1823.10 | 8701.77 | 419.56 |
| TCGA-FG-8181-01A-11R-2256-07 | OA | 28.73 | 0 | 14.14 | 1719.56 | 247.05 | 256.08 | 1534.56 | 747.05 | 2055.38 | 1802.44 |
| TCGA-DU-8162-01A-21R-2256-07 | OA | 14.80 | 1 | 100.68 | 1885.63 | 456.46 | 13256.14 | 812.91 | 4987.58 | 2415.07 | 1818.40 |
| TCGA-S9-A6WI-01A-21R-A33Z-07 | OA | 86.17 | 0 | 11.48 | 1793.39 | 176.00 | 574.16 | 707.83 | 7890.78 | 1746.78 | 1160.70 |
| TCGA-FG-7643-01A-11R-2090-07 | OA | 20.37 | 0 | 61.28 | 2144.67 | 430.38 | 1841.54 | 827.41 | 8535.90 | 2104.79 | 1011.60 |
| TCGA-S9-A7IQ-01A-21R-A34F-07 | OA | 36.63 | 0 | 13.27 | 1755.62 | 109.60 | 746.08 | 762.42 | 1216.81 | 1682.44 | 1326.41 |
| TCGA-DB-A64O-01A-11R-A29R-07 | OA | 25.83 | 1 | 33.90 | 3119.15 | 630.50 | 1605.19 | 1140.02 | 3985.89 | 2093.86 | 288.39 |
| TCGA-TQ-A7RP-01A-21R-A34F-07 | OA | 30.47 | 0 | 34.42 | 2017.76 | 495.47 | 2062.25 | 2382.13 | 6774.33 | 2244.07 | 150.51 |
| TCGA-FG-A713-01A-11R-A32Q-07 | OA | 20.73 | 0 | 27.17 | 1469.17 | 154.81 | 1184.94 | 542.48 | 5755.93 | 1440.71 | 1805.09 |
| TCGA-HT-8018-01A-11R-2404-07 | OA | 38.40 | 1 | 45.94 | 1790.87 | 980.35 | 1114.69 | 692.02 | 804.50 | 8099.81 | 1444.55 |
| TCGA-HT-7474-01A-11R-2027-07 | OA | 3.80 | 0 | 38.71 | 2054.16 | 284.83 | 1213.51 | 691.11 | 7767.02 | 3709.49 | 1708.60 |
| TCGA-DU-8161-01A-11R-2256-07 | OA | 24.07 | 1 | 149.82 | 2747.88 | 2033.29 | 7109.50 | 571.43 | 1804.78 | 10295.70 | 213.98 |
| TCGA-HT-7880-01A-11R-2403-07 | OA | 5.40 | 0 | 24.43 | 1764.28 | 214.47 | 867.99 | 891.18 | 11877.22 | 1271.57 | 1571.07 |
| TCGA-QH-A6CY-01A-11R-A32Q-07 | OA | 13.60 | 0 | 49.22 | 2717.59 | 262.23 | 1014.66 | 943.40 | 6966.83 | 2195.10 | 1233.19 |
| TCGA-HT-7608-01A-11R-2090-07 | OA | 22.37 | 0 | 72.66 | 2223.30 | 493.84 | 2709.95 | 777.30 | 905.05 | 1540.62 | 1381.73 |
| TCGA-DB-A64Q-01A-11R-A29R-07 | OA | 6.07 | 0 | 57.84 | 3467.81 | 435.68 | 1708.66 | 1471.96 | 1368.58 | 1740.26 | 1324.27 |
| TCGA-DB-5276-01A-01R-1470-07 | OA | 73.93 | 0 | 92.83 | 1891.45 | 1051.50 | 909.09 | 832.18 | 14822.75 | 2506.15 | 1360.70 |
| TCGA-HT-7610-01A-21R-2090-07 | OA | 56.87 | 0 | 59.43 | 2289.16 | 316.98 | 5740.38 | 937.92 | 1945.59 | 1726.85 | 1514.08 |
| TCGA-QH-A6CZ-01A-11R-A32Q-07 | OA | 9.30 | 0 | 40.26 | 2965.32 | 208.85 | 3905.87 | 929.85 | 1088.48 | 2756.88 | 3652.05 |
| TCGA-HT-7684-01A-11R-2256-07 | OA | 6.13 | 0 | 2.84 | 2785.34 | 439.19 | 990.46 | 1416.56 | 2604.99 | 1426.23 | 491.53 |
| TCGA-TQ-A7RR-01A-21R-A34F-07 | OA | 26.23 | 0 | 97.96 | 2279.54 | 725.06 | 1917.52 | 972.73 | 11428.94 | 1480.06 | 612.55 |
| TCGA-DU-7304-01A-12R-2090-07 | OA | 23.63 | 1 | 135.71 | 2117.73 | 1033.84 | 2972.13 | 866.76 | 1474.09 | 1581.60 | 612.97 |
| TCGA-HT-7473-01A-11R-2027-07 | OA | 16.77 | 0 | 158.62 | 3347.56 | 3310.56 | 2625.13 | 1260.56 | 870.41 | 3363.68 | 291.60 |
| TCGA-S9-A6WH-01A-12R-A33Z-07 | OA | 39.10 | 0 | 29.45 | 2486.99 | 212.37 | 2483.86 | 752.35 | 140.03 | 1383.79 | 894.97 |
| TCGA-DU-5851-01A-13R-1896-07 | OA | 17.70 | 0 | 58.03 | 1704.79 | 477.40 | 2253.69 | 796.56 | 8017.54 | 1899.12 | 1384.95 |
| TCGA-IK-8125-01A-11R-2256-07 | OA | 43.37 | 0 | 88.59 | 2961.03 | 408.36 | 3816.94 | 869.51 | 501.58 | 1620.80 | 1250.41 |
| TCGA-HT-7902-01A-12R-2403-07 | OA | 31.87 | 0 | 65.06 | 2137.13 | 372.16 | 526.74 | 1094.98 | 33312.24 | 4688.50 | 1825.56 |
| TCGA-HW-7489-01A-11R-2027-07 | OA | 42.07 | 1 | 21.94 | 2063.04 | 352.72 | 1372.21 | 861.77 | 1982.95 | 1562.34 | 821.38 |
| TCGA-P5-A77X-01A-11R-A32Q-07 | OA | 3.50 | 0 | 49.36 | 2949.10 | 171.32 | 2085.93 | 849.65 | 1850.23 | 1290.59 | 701.99 |
| TCGA-P5-A731-01A-11R-A32Q-07 | OA | 9.13 | 0 | 122.51 | 2995.13 | 446.26 | 1151.25 | 2096.86 | 821.76 | 3751.88 | 736.84 |
| TCGA-DB-5270-01A-02R-1470-07 | OA | 124.43 | 0 | 42.62 | 2444.60 | 643.37 | 1646.09 | 876.58 | 3474.94 | 2590.18 | 1126.83 |
| TCGA-FG-8187-01A-11R-2256-07 | OA | 20.37 | 0 | 37.32 | 2696.44 | 357.23 | 2193.21 | 1209.22 | 20874.21 | 1581.55 | 813.42 |
| TCGA-S9-A6WQ-01A-12R-A34F-07 | OA | 14.27 | 0 | 25.55 | 2873.00 | 1034.87 | 1901.31 | 1086.38 | 13025.23 | 1300.81 | 495.86 |
| TCGA-HT-7482-01A-11R-2027-07 | OA | 108.43 | 0 | 116.57 | 3000.48 | 1602.76 | 1311.52 | 1022.94 | 15323.61 | 1606.56 | 546.02 |
| TCGA-FG-A70Z-01A-12R-A33Z-07 | OA | 10.93 | 0 | 92.95 | 3956.20 | 1162.49 | 12313.52 | 1619.53 | 608.49 | 3192.58 | 477.43 |
| TCGA-FN-7833-01A-11R-2090-07 | OA | 27.90 | 0 | 142.43 | 2612.02 | 2201.41 | 2732.92 | 890.58 | 1335.68 | 24530.79 | 197.70 |
| TCGA-HT-7681-01A-11R-2403-07 | OA | 45.30 | 0 | 30.68 | 2550.61 | 415.69 | 1042.11 | 710.45 | 2252.73 | 2564.83 | 2371.88 |
| TCGA-DH-5143-01A-01R-1470-07 | OA | 46.70 | 0 | 104.31 | 2227.62 | 833.40 | 3255.80 | 721.60 | 966.13 | 2371.57 | 446.42 |
| TCGA-TM-A84H-01A-11R-A36H-07 | OA | 30.87 | 0 | 129.13 | 3466.81 | 350.02 | 4788.07 | 1480.59 | 138.92 | 1414.58 | 264.78 |
| TCGA-CS-6186-01A-12R-2027-07 | OA | 17.93 | 1 | 78.76 | 3716.66 | 433.93 | 3142.04 | 889.66 | 705.39 | 7023.65 | 116.90 |
| TCGA-DU-7011-01A-11R-2027-07 | OA | 106.67 | 1 | 72.26 | 3527.77 | 1532.23 | 3198.80 | 838.94 | 1893.92 | 2356.49 | 607.47 |
| TCGA-FG-A60J-01A-11R-A28M-07 | OA | 14.97 | 0 | 52.58 | 2570.83 | 842.06 | 3373.64 | 1916.96 | 3553.52 | 3568.54 | 1155.85 |
| TCGA-QH-A6X3-01A-21R-A32Q-07 | OA | 10.43 | 0 | 183.95 | 3492.96 | 778.06 | 3567.60 | 2272.73 | 962.87 | 1392.23 | 346.14 |
| TCGA-DB-A4XH-01A-11R-A27Q-07 | OA | 32.07 | 0 | 36.98 | 3173.73 | 171.11 | 3691.39 | 790.40 | 4489.58 | 1532.22 | 1039.33 |
| TCGA-S9-A7IY-01A-11R-A34F-07 | OA | 23.83 | 0 | 34.61 | 2272.68 | 572.50 | 1837.14 | 949.49 | 3224.04 | 1747.43 | 805.43 |
| TCGA-QH-A6X5-01A-12R-A32Q-07 | OA | 16.57 | 0 | 34.00 | 3094.29 | 323.22 | 828.86 | 928.00 | 16458.37 | 1448.51 | 1120.50 |
| TCGA-DU-5852-01A-11R-1708-07 | OA | 6.83 | 1 | 155.84 | 2729.03 | 1267.11 | 67520.30 | 974.38 | 647.95 | 2543.35 | 410.67 |
| TCGA-QH-A870-01A-11R-A36H-07 | OA | 13.17 | 0 | 93.83 | 2314.54 | 358.59 | 1603.94 | 1884.58 | 1043.61 | 1450.22 | 329.07 |
| TCGA-TQ-A7RG-01A-11R-A33Z-07 | OA | 19.97 | 0 | 48.70 | 2896.08 | 652.32 | 1873.30 | 917.48 | 2647.15 | 1544.71 | 1254.95 |
| TCGA-HT-8114-01A-11R-2404-07 | OA | 34.67 | 0 | 231.87 | 2857.54 | 1093.12 | 5150.78 | 1187.63 | 19.92 | 4651.15 | 364.61 |
| TCGA-HT-A74L-01A-11R-A32Q-07 | OA | 11.20 | 0 | 143.60 | 3115.54 | 732.14 | 11783.21 | 1718.08 | 1821.04 | 2171.27 | 2631.01 |
| TCGA-DB-A4XC-01A-11R-A26U-07 | OA | 47.37 | 0 | 93.17 | 2178.69 | 939.37 | 3793.23 | 922.78 | 320.36 | 3223.36 | 864.71 |
| TCGA-DB-5274-01A-01R-1470-07 | OA | 76.30 | 0 | 0.57 | 3567.24 | 788.51 | 573.56 | 627.59 | 3536.21 | 1444.83 | 559.77 |
| TCGA-DH-5140-01A-01R-1470-07 | OA | 20.23 | 1 | 157.16 | 4612.12 | 428.06 | 1057.67 | 968.89 | 42.13 | 2393.39 | 1425.15 |
| TCGA-RY-A845-01A-11R-A36H-07 | OA | 19.87 | 0 | 66.17 | 3637.51 | 434.77 | 2271.64 | 1466.87 | 816.33 | 1891.22 | 383.71 |
| TCGA-DU-5855-01A-11R-1708-07 | OA | 6.90 | 0 | 217.02 | 2675.82 | 747.37 | 4768.34 | 1113.26 | 178.03 | 1943.71 | 395.73 |
| TCGA-QH-A6XA-01A-12R-A32Q-07 | OA | 16.73 | 0 | 64.17 | 3323.08 | 452.32 | 1759.32 | 1176.92 | 6458.56 | 1254.90 | 571.75 |
| TCGA-HT-7611-01A-11R-2403-07 | OA | 58.40 | 0 | 235.48 | 2401.61 | 1160.01 | 3813.20 | 1002.09 | 2035.21 | 1966.96 | 299.70 |
| TCGA-TM-A84L-01A-11R-A36H-07 | OA | 41.40 | 1 | 48.67 | 2128.64 | 829.39 | 1444.29 | 1527.69 | 4446.20 | 1559.01 | 497.34 |
| TCGA-P5-A77W-01A-11R-A32Q-07 | OA | 19.47 | 0 | 39.26 | 3699.25 | 197.09 | 660.36 | 1091.87 | 1621.52 | 1899.10 | 1351.00 |
| TCGA-QH-A65S-01A-11R-A29R-07 | OA | 2.57 | 0 | 34.88 | 2928.40 | 878.21 | 1071.57 | 1178.09 | 13934.52 | 1184.21 | 616.89 |
| TCGA-DU-A5TR-01A-11R-A28M-07 | OA | 30.27 | 0 | 21.06 | 3065.41 | 1344.79 | 284.92 | 1496.12 | 1460.09 | 1882.48 | 257.21 |
| TCGA-DU-8167-01A-11R-2256-07 | OA | 15.70 | 0 | 62.53 | 3810.32 | 917.98 | 1398.64 | 1399.48 | 9154.77 | 2107.76 | 519.85 |
| TCGA-QH-A6X4-01A-51R-A32Q-07 | OA | 14.73 | 0 | 31.07 | 2166.09 | 207.90 | 2508.12 | 810.51 | 5264.29 | 1062.52 | 270.81 |
| TCGA-S9-A7QY-01A-11R-A34F-07 | OA | 28.77 | 0 | 35.62 | 2375.94 | 217.94 | 2280.29 | 1318.52 | 7339.90 | 2063.29 | 1312.66 |
| TCGA-HT-7483-01A-11R-2027-07 | OA | 175.17 | 0 | 142.70 | 3551.61 | 492.94 | 3316.18 | 1024.63 | 147.05 | 1658.82 | 632.38 |
| TCGA-DB-A4XE-01A-11R-A27Q-07 | OA | 37.97 | 0 | 77.30 | 4306.11 | 349.89 | 2779.83 | 1193.73 | 2543.88 | 1701.41 | 1378.05 |
| TCGA-P5-A730-01A-11R-A32Q-07 | OA | 11.10 | 0 | 132.13 | 3517.59 | 217.21 | 2298.46 | 1617.87 | 3413.50 | 2113.59 | 282.32 |
| TCGA-DU-7306-01A-11R-2090-07 | OA | 42.57 | 0 | 180.74 | 2289.02 | 588.51 | 3513.90 | 2026.79 | 1108.20 | 8364.33 | 271.11 |
| TCGA-HT-8111-01A-11R-2404-07 | OA | 0.23 | 0 | 79.56 | 2660.89 | 1032.89 | 1254.22 | 933.88 | 10948.02 | 2455.09 | 371.64 |
| TCGA-FG-A60K-01A-11R-A29R-07 | OA | 18.07 | 0 | 124.91 | 2965.17 | 504.29 | 1966.74 | 1077.17 | 1753.78 | 1885.89 | 1046.68 |
| TCGA-DB-A4X9-01A-11R-A26U-07 | OA | 47.07 | 0 | 68.34 | 3053.21 | 430.36 | 2544.58 | 1194.57 | 846.11 | 1417.84 | 364.11 |
| TCGA-KT-A74X-01A-11R-A32Q-07 | OA | 14.60 | 0 | 68.30 | 3619.17 | 671.26 | 1699.51 | 887.65 | 1933.13 | 2800.11 | 453.15 |
| TCGA-TM-A7C5-01A-11R-A32Q-07 | OA | 50.00 | 0 | 58.82 | 3089.97 | 265.28 | 2044.95 | 986.16 | 168.97 | 1551.33 | 703.58 |
| TCGA-FG-8186-01A-11R-2256-07 | OA | 16.23 | 0 | 75.45 | 2833.18 | 870.21 | 4150.87 | 1747.65 | 1393.85 | 6814.57 | 1472.05 |
| TCGA-DB-5275-01A-01R-1470-07 | OA | 48.60 | 0 | 66.32 | 3138.55 | 361.91 | 2172.43 | 1147.71 | 216.95 | 2053.15 | 237.61 |
| TCGA-DU-7019-01A-11R-2027-07 | OA | 26.67 | 0 | 102.50 | 3476.93 | 855.92 | 2097.20 | 1066.57 | 406.66 | 2454.95 | 506.16 |
| TCGA-E1-A7YW-01A-11R-A34R-07 | OA | 37.33 | 1 | 159.41 | 2875.85 | 424.49 | 2388.37 | 979.44 | 1726.43 | 1402.55 | 180.54 |
| TCGA-VV-A829-01A-21R-A36H-07 | OA | 37.57 | 0 | 79.15 | 4424.32 | 711.95 | 6681.30 | 983.68 | 16.32 | 1864.14 | 83.64 |
| TCGA-DU-5871-01A-12R-1708-07 | OA | 19.20 | 0 | 176.67 | 2666.91 | 905.68 | 3943.53 | 1156.00 | 134.76 | 1453.33 | 425.42 |
| TCGA-S9-A6WP-01A-12R-A34F-07 | OA | 18.90 | 0 | 56.74 | 2965.37 | 921.39 | 2683.67 | 783.20 | 5222.73 | 1688.23 | 920.00 |
| TCGA-E1-A7YU-01A-11R-A34R-07 | OA | 0.77 | 1 | 42.79 | 2596.62 | 241.98 | 820.00 | 986.45 | 5228.43 | 942.71 | 244.83 |
| TCGA-DB-A64S-01A-11R-A29R-07 | OA | 27.83 | 0 | 44.25 | 3251.23 | 1111.11 | 912.48 | 1201.08 | 2896.76 | 2097.35 | 367.26 |
| TCGA-FG-7637-01A-11R-2090-07 | OA | 40.63 | 0 | 134.06 | 4192.95 | 234.77 | 16873.50 | 960.37 | 92.21 | 1476.74 | 858.95 |
| TCGA-TQ-A7RO-01A-11R-A33Z-07 | OA | 31.63 | 0 | 140.72 | 2423.08 | 295.48 | 3631.10 | 971.95 | 84.62 | 2070.14 | 1785.07 |
| TCGA-TQ-A7RK-01A-11R-A33Z-07 | OA | 44.70 | 0 | 66.17 | 3269.06 | 361.53 | 839.58 | 1272.57 | 1751.10 | 1326.03 | 171.78 |
| TCGA-DU-5872-01A-11R-1708-07 | OA | 17.73 | 0 | 93.68 | 2913.25 | 1018.04 | 2208.85 | 1994.45 | 1306.73 | 5198.13 | 332.41 |
| TCGA-DU-5853-01A-11R-1896-07 | OA | 13.57 | 0 | 80.49 | 3006.07 | 1347.45 | 4170.57 | 1002.02 | 3334.55 | 2160.39 | 645.04 |
| TCGA-DU-8163-01A-11R-2256-07 | OA | 20.97 | 0 | 112.18 | 3596.35 | 1292.95 | 4831.52 | 1138.09 | 6585.86 | 2135.54 | 818.61 |
| TCGA-DB-A75K-01A-11R-A32Q-07 | OA | 12.27 | 0 | 51.46 | 1965.29 | 524.81 | 3017.46 | 626.55 | 3161.44 | 9645.37 | 348.04 |
| TCGA-HT-A74J-01A-12R-A32Q-07 | OA | 10.67 | 0 | 53.66 | 2613.59 | 331.03 | 2417.91 | 1084.81 | 988.75 | 1640.42 | 243.62 |
| TCGA-DB-A4XA-01A-11R-A26U-07 | OA | 19.10 | 0 | 22.64 | 2528.44 | 468.80 | 1082.25 | 942.57 | 3220.32 | 1297.07 | 1249.59 |
| TCGA-WY-A85D-01A-11R-A36H-07 | OA | 38.23 | 0 | 24.16 | 2839.25 | 722.88 | 500.97 | 1669.63 | 3870.32 | 1125.25 | 254.93 |
| TCGA-HT-7475-01A-11R-2027-07 | OA | 17.67 | 0 | 63.71 | 2980.36 | 205.97 | 4223.77 | 1517.41 | 2284.54 | 1488.03 | 543.65 |
| TCGA-TQ-A7RH-01A-12R-A34F-07 | OA | 16.20 | 0 | 67.48 | 2685.47 | 1134.96 | 3876.31 | 1713.31 | 1870.76 | 1305.76 | 343.50 |
| TCGA-WY-A85E-01A-11R-A36H-07 | OA | 21.10 | 0 | 45.37 | 4931.57 | 249.15 | 805.65 | 1224.57 | 339.13 | 2203.40 | 956.14 |
| TCGA-QH-A6CV-01A-11R-A31N-07 | OA | 14.73 | 0 | 37.33 | 3613.53 | 1141.91 | 30874.63 | 1754.67 | 2786.55 | 2704.12 | 110.03 |
| TCGA-TQ-A7RJ-01A-11R-A33Z-07 | OA | 40.97 | 0 | 83.82 | 2378.66 | 867.64 | 3284.64 | 1151.77 | 346.91 | 1299.10 | 588.59 |
| TCGA-HT-7609-01A-11R-2090-07 | OA | 46.63 | 0 | 144.75 | 3114.40 | 687.16 | 3408.16 | 1154.47 | 1226.07 | 1783.27 | 232.68 |
| TCGA-E1-5322-01A-01R-1470-07 | OA | 132.60 | 1 | 88.21 | 3556.65 | 1161.16 | 2436.84 | 960.74 | 106.14 | 2872.68 | 165.43 |
| TCGA-FG-5965-01B-11R-1896-07 | OA | 37.33 | 1 | 108.62 | 2315.26 | 605.59 | 3782.72 | 826.83 | 479.11 | 2657.44 | 439.11 |
| TCGA-TQ-A7RM-01A-11R-A33Z-07 | OA | 37.20 | 0 | 208.71 | 4278.61 | 135.49 | 2033.65 | 2066.48 | 12.96 | 1609.66 | 451.73 |
| TCGA-E1-A7YV-01A-11R-A34R-07 | OA | 32.90 | 1 | 346.21 | 3340.66 | 168.12 | 1854.70 | 1134.70 | 131.42 | 2122.58 | 224.31 |
| TCGA-DU-8166-01A-11R-2256-07 | OA | 17.20 | 0 | 122.47 | 3603.11 | 569.53 | 7254.73 | 1458.88 | 427.07 | 1710.17 | 557.16 |
| TCGA-DB-A64W-01A-11R-A29R-07 | OA | 14.60 | 1 | 154.09 | 3520.43 | 73.71 | 2369.88 | 776.20 | 914.74 | 1695.83 | 904.53 |
| TCGA-DB-5281-01A-01R-1470-07 | OA | 79.37 | 0 | 205.34 | 2302.30 | 412.44 | 1715.45 | 753.59 | 634.57 | 2613.99 | 628.68 |
| TCGA-DU-6542-01A-11R-1896-07 | OA | 2.43 | 0 | 130.40 | 3130.29 | 1231.91 | 1976.45 | 1023.04 | 2519.77 | 3400.74 | 311.90 |
| TCGA-HT-7690-01A-11R-2256-07 | OA | 0.10 | 0 | 77.19 | 3584.08 | 1066.80 | 1318.44 | 948.05 | 2061.50 | 3065.81 | 688.77 |
| TCGA-HT-7692-01A-12R-2256-07 | OA | 3.00 | 0 | 33.11 | 4055.56 | 474.51 | 2807.05 | 986.30 | 1969.94 | 2947.87 | 1702.82 |
| TCGA-HT-7873-01B-11R-2403-07 | OA | 23.93 | 0 | 119.38 | 2574.00 | 393.46 | 3539.39 | 1036.79 | 5712.18 | 1308.26 | 327.06 |
| TCGA-DU-6396-01A-11R-1708-07 | OA | 76.20 | 1 | 224.86 | 2744.07 | 2595.72 | 1507.82 | 1106.58 | 2949.21 | 9556.30 | 152.69 |
| TCGA-DB-A64U-01A-11R-A29R-07 | OA | 28.20 | 0 | 44.88 | 3086.69 | 363.64 | 1341.38 | 947.83 | 13570.00 | 1975.83 | 1191.41 |
| TCGA-DB-5280-01A-01R-1470-07 | OA | 37.07 | 0 | 195.10 | 2321.61 | 608.83 | 5870.99 | 956.22 | 1589.26 | 1797.70 | 275.81 |
| TCGA-HT-8013-01A-11R-2403-07 | OA | 64.43 | 1 | 116.15 | 3731.50 | 1984.16 | 2456.57 | 1219.70 | 2343.77 | 1899.39 | 310.13 |
| TCGA-P5-A737-01A-11R-A32Q-07 | OA | 12.40 | 0 | 84.83 | 2563.26 | 256.97 | 5312.28 | 813.81 | 965.72 | 2005.92 | 989.40 |
| TCGA-DU-A6S6-01A-21R-A32Q-07 | OA | 96.43 | 0 | 29.20 | 1864.05 | 78.99 | 3191.85 | 826.71 | 645.76 | 1210.63 | 1337.00 |
| TCGA-FG-8188-01A-11R-2256-07 | OA | 15.17 | 0 | 101.93 | 2598.71 | 413.16 | 1763.45 | 1048.49 | 9927.76 | 1850.57 | 286.00 |
| TCGA-DU-A7T8-01A-21R-A34R-07 | OA | 140.97 | 1 | 73.08 | 2958.78 | 532.43 | 2169.99 | 1287.69 | 54.00 | 3496.25 | 286.19 |
| TCGA-FG-A4MY-01A-11R-A26U-07 | OA | 24.03 | 0 | 97.14 | 2616.65 | 529.89 | 582.82 | 1254.73 | 10070.67 | 1481.83 | 363.09 |
| TCGA-DU-6403-01A-11R-1708-07 | OA | 11.80 | 1 | 270.52 | 5265.00 | 1235.22 | 24642.14 | 1015.16 | 9639.25 | 3871.75 | 293.30 |
| TCGA-HT-A614-01A-11R-A29R-07 | OA | 2.73 | 0 | 18.05 | 3100.09 | 502.86 | 475.52 | 1127.17 | 6936.67 | 1254.96 | 498.96 |
| TCGA-FG-A4MW-01A-11R-A26U-07 | OA | 18.63 | 1 | 120.98 | 3276.16 | 492.87 | 42612.68 | 1672.45 | 5712.32 | 4057.05 | 254.34 |
| TCGA-DU-6395-01A-13R-1708-07 | OA | 49.70 | 1 | 94.02 | 3441.77 | 566.93 | 2055.88 | 1045.43 | 3734.67 | 1402.29 | 183.50 |
| TCGA-HT-7879-01A-11R-2403-07 | OA | 3.73 | 0 | 84.22 | 2883.95 | 503.65 | 2121.63 | 1131.63 | 4550.07 | 1264.26 | 628.98 |
| TCGA-FG-A4MU-01B-11R-A28M-07 | OA | 10.87 | 0 | 211.59 | 3943.71 | 1396.06 | 22441.04 | 1879.62 | 14.38 | 4641.74 | 223.50 |
| TCGA-S9-A6TV-01A-12R-A34R-07 | OA | 19.03 | 0 | 158.11 | 2608.51 | 1381.38 | 616.67 | 1099.04 | 3.86 | 8238.65 | 676.13 |
| TCGA-TM-A84T-01A-11R-A36H-07 | OA | 24.13 | 0 | 79.74 | 3035.23 | 676.40 | 3648.51 | 1067.69 | 4211.40 | 1358.83 | 219.29 |
| TCGA-DU-6406-01A-11R-1708-07 | OA | 17.07 | 1 | 227.75 | 4141.88 | 568.45 | 39475.42 | 1520.85 | 46.67 | 15053.20 | 244.56 |
| TCGA-QH-A6CW-01A-11R-A32Q-07 | OA | 13.80 | 0 | 124.94 | 2935.76 | 462.82 | 4062.05 | 922.61 | 704.10 | 883.66 | 176.02 |
| TCGA-QH-A65X-01A-11R-A32Q-07 | OA | 16.07 | 0 | 0.53 | 2787.45 | 454.64 | 779.00 | 658.23 | 328.59 | 1202.53 | 404.01 |
| TCGA-DU-A7TD-01A-12R-A34F-07 | OA | 7.60 | 1 | 266.75 | 6150.45 | 1532.64 | 120100.11 | 2464.33 | 626.03 | 18474.71 | 380.03 |
| TCGA-CS-6669-01A-11R-1896-07 | OD | 49.80 | 0 | 32.32 | 1410.56 | 57.35 | 406.92 | 619.03 | 4350.02 | 1997.27 | 1238.05 |
| TCGA-HT-8558-01A-21R-2404-07 | OD | 13.93 | 0 | 22.67 | 1686.65 | 134.62 | 245.86 | 760.61 | 16139.58 | 1926.49 | 1169.07 |
| TCGA-HT-8107-01A-13R-2404-07 | OD | 0.47 | 0 | 40.82 | 1483.01 | 203.06 | 1013.57 | 706.05 | 5993.95 | 2168.64 | 1284.10 |
| TCGA-FG-8189-01B-11R-A28M-07 | OD | 22.83 | 0 | 17.78 | 1468.76 | 105.79 | 419.96 | 635.66 | 1311.90 | 2688.55 | 2367.53 |
| TCGA-HT-8019-01A-21R-2404-07 | OD | 33.47 | 0 | 18.87 | 1615.59 | 92.64 | 416.82 | 613.30 | 2845.61 | 1383.56 | 1124.95 |
| TCGA-E1-A7YY-01A-11R-A34R-07 | OD | 148.17 | 1 | 22.50 | 1440.12 | 253.62 | 417.22 | 695.27 | 1377.19 | 2213.20 | 1033.18 |
| TCGA-HT-8113-01A-11R-2404-07 | OD | 30.00 | 0 | 21.44 | 1684.87 | 147.40 | 1168.03 | 771.41 | 2970.85 | 1877.39 | 1591.96 |
| TCGA-HT-A61A-01A-11R-A29R-07 | OD | 6.47 | 0 | 29.04 | 1756.91 | 238.61 | 507.21 | 792.30 | 6159.82 | 2007.62 | 1460.70 |
| TCGA-HT-7603-01A-21R-2090-07 | OD | 23.50 | 0 | 16.86 | 2365.71 | 258.14 | 305.81 | 997.20 | 3974.53 | 1703.18 | 1004.01 |
| TCGA-HT-7874-01A-11R-2403-07 | OD | 37.67 | 0 | 42.90 | 1866.96 | 189.34 | 2095.83 | 719.50 | 5341.49 | 1999.66 | 1835.79 |
| TCGA-TM-A84R-01A-21R-A36H-07 | OD | 18.87 | 0 | 27.25 | 1766.38 | 165.10 | 1202.94 | 674.81 | 2503.31 | 1822.08 | 1436.59 |
| TCGA-P5-A5F6-01A-11R-A28M-07 | OD | 1.30 | 0 | 14.49 | 1477.05 | 302.54 | 495.16 | 1855.07 | 0.60 | 846.01 | 111.11 |
| TCGA-DU-A7TG-01A-21R-A34R-07 | OD | 45.03 | 1 | 14.17 | 1556.25 | 311.80 | 825.78 | 736.43 | 1487.03 | 2391.34 | 1318.05 |
| TCGA-DB-A64L-01A-11R-A29R-07 | OD | 24.53 | 0 | 29.24 | 2204.31 | 618.60 | 2404.70 | 867.34 | 352.69 | 2102.22 | 1242.77 |
| TCGA-HT-7694-01A-11R-2256-07 | OD | 7.00 | 0 | 33.55 | 1843.12 | 171.35 | 1671.08 | 681.80 | 6262.13 | 1702.35 | 1659.60 |
| TCGA-HT-8105-01A-11R-2404-07 | OD | 6.33 | 0 | 94.41 | 2738.10 | 531.90 | 2761.44 | 756.89 | 457.56 | 1512.80 | 854.26 |
| TCGA-QH-A6CU-01A-11R-A31N-07 | OD | 15.03 | 0 | 7.79 | 2745.13 | 597.66 | 191.99 | 1986.09 | 12032.83 | 918.20 | 996.10 |
| TCGA-HT-7881-01A-11R-2403-07 | OD | 35.97 | 0 | 22.69 | 1960.38 | 111.75 | 720.95 | 937.69 | 16016.93 | 2057.57 | 2472.06 |
| TCGA-HT-A617-01A-11R-A29R-07 | OD | 16.37 | 0 | 27.62 | 3766.47 | 3159.88 | 1615.31 | 1221.90 | 1960.76 | 2181.69 | 193.31 |
| TCGA-HT-8010-01A-11R-2403-07 | OD | 1.67 | 0 | 35.34 | 2047.85 | 378.92 | 1560.76 | 891.52 | 3170.98 | 1606.78 | 2866.99 |
| TCGA-HW-7487-01A-11R-2027-07 | OD | 46.07 | 0 | 53.74 | 2338.68 | 202.75 | 2453.35 | 753.22 | 3620.23 | 1750.73 | 972.23 |
| TCGA-DB-5279-01A-03R-2347-07 | OD | 45.13 | 0 | 57.57 | 2788.69 | 476.61 | 4046.42 | 1233.57 | 84.40 | 1708.54 | 943.74 |
| TCGA-FG-5962-01B-11R-1896-07 | OD | 48.43 | 0 | 56.19 | 2314.60 | 267.62 | 3169.19 | 1049.21 | 996.83 | 5503.17 | 952.06 |
| TCGA-HT-7616-01A-11R-2256-07 | OD | 0.23 | 1 | 84.21 | 3042.48 | 1910.09 | 5553.79 | 1669.35 | 465.14 | 7602.72 | 551.83 |
| TCGA-CS-5396-01A-02R-1470-07 | OD | 54.37 | 0 | 141.12 | 2484.55 | 1090.27 | 2053.83 | 738.09 | 2.76 | 4457.70 | 116.57 |
| TCGA-HT-8109-01A-11R-2404-07 | OD | 5.63 | 0 | 71.57 | 2471.86 | 418.65 | 3821.61 | 765.74 | 3995.92 | 1780.29 | 1274.13 |
| TCGA-DU-A5TT-01A-11R-A28M-07 | OD | 24.77 | 0 | 58.74 | 4850.13 | 465.19 | 1928.97 | 1765.73 | 3018.32 | 4124.06 | 131.31 |
| TCGA-DU-7302-01A-11R-2090-07 | OD | 61.13 | 0 | 61.35 | 2466.60 | 179.96 | 3513.62 | 681.32 | 819.02 | 2478.87 | 1222.56 |
| TCGA-E1-5319-01A-01R-1896-07 | OD | 96.90 | 1 | 53.41 | 2187.95 | 652.21 | 1931.28 | 629.32 | 6551.00 | 2310.84 | 1948.59 |
| TCGA-VM-A8CA-01A-11R-A36H-07 | OD | 13.70 | 0 | 4.44 | 2010.27 | 419.83 | 424.82 | 1180.06 | 328.75 | 1185.06 | 591.98 |
| TCGA-DU-A7TB-01A-11R-A33Z-07 | OD | 52.23 | 0 | 51.32 | 3223.35 | 221.18 | 112.56 | 1082.76 | 3752.80 | 2264.19 | 387.78 |
| TCGA-DU-7300-01A-21R-2090-07 | OD | 62.87 | 1 | 62.22 | 2366.51 | 660.54 | 1824.75 | 692.31 | 2173.57 | 3666.75 | 2115.99 |
| TCGA-FG-7641-01B-11R-2256-07 | OD | 20.90 | 0 | 76.64 | 2524.48 | 83.38 | 5034.61 | 1046.85 | 4614.80 | 1512.58 | 1574.06 |
| TCGA-HT-7467-01A-11R-2027-07 | OD | 0.10 | 0 | 68.32 | 1910.94 | 292.09 | 2429.15 | 770.94 | 21575.59 | 3259.19 | 878.94 |
| TCGA-HT-7620-01A-11R-2256-07 | OD | 14.47 | 0 | 31.09 | 2504.22 | 512.64 | 3326.61 | 1290.83 | 20602.74 | 1336.14 | 1459.96 |
| TCGA-HT-7481-01A-11R-2027-07 | OD | 97.27 | 0 | 81.07 | 3101.72 | 239.36 | 4678.08 | 813.37 | 15218.13 | 1567.19 | 3012.20 |
| TCGA-FG-8191-01A-11R-2256-07 | OD | 33.07 | 0 | 113.41 | 2394.77 | 868.05 | 2500.17 | 1493.64 | 13114.87 | 1670.30 | 259.91 |
| TCGA-HT-7856-01A-11R-2403-07 | OD | 39.63 | 0 | 55.35 | 1840.85 | 243.25 | 1995.82 | 692.86 | 5099.10 | 2100.41 | 1166.14 |
| TCGA-HT-7605-01A-11R-2090-07 | OD | 4.63 | 0 | 35.21 | 2494.99 | 362.73 | 2004.56 | 1016.03 | 7888.63 | 1864.59 | 2336.67 |
| TCGA-HT-7602-01A-21R-2090-07 | OD | 30.27 | 0 | 47.30 | 2519.34 | 612.79 | 552.69 | 1194.05 | 28634.07 | 1584.82 | 1979.00 |
| TCGA-P5-A5EX-01A-12R-A28M-07 | OD | 8.10 | 0 | 25.75 | 2783.02 | 1440.63 | 1015.25 | 1786.36 | 52.46 | 3710.06 | 583.69 |
| TCGA-E1-A7Z2-01A-21R-A34R-07 | OD | 13.27 | 1 | 54.97 | 4388.88 | 273.61 | 7159.77 | 823.30 | 2628.24 | 1363.55 | 220.70 |
| TCGA-DU-A6S2-01A-21R-A32Q-07 | OD | 25.90 | 0 | 56.99 | 1619.77 | 146.48 | 2615.61 | 888.69 | 4911.84 | 1275.16 | 1284.95 |
| TCGA-DU-A76R-01A-11R-A32Q-07 | OD | 21.60 | 1 | 53.26 | 2706.54 | 309.51 | 2684.64 | 1717.34 | 495.91 | 1722.58 | 3318.13 |
| TCGA-DU-A76L-01A-11R-A32Q-07 | OD | 27.13 | 1 | 149.77 | 5361.57 | 1308.62 | 266.62 | 1868.76 | 3529.88 | 14007.56 | 583.96 |
| TCGA-HT-A5R5-01A-11R-A28M-07 | OD | 0.13 | 0 | 157.52 | 1797.85 | 818.88 | 1488.95 | 1250.90 | 375.06 | 3920.99 | 515.14 |
| TCGA-HW-7495-01A-11R-2027-07 | OD | 35.93 | 0 | 68.30 | 2021.49 | 154.64 | 2080.56 | 1166.54 | 8744.05 | 1404.84 | 1744.44 |
| TCGA-DH-A66G-01A-21R-A31N-07 | OD | 17.43 | 0 | 92.66 | 1914.95 | 741.71 | 1017.47 | 944.43 | 61.77 | 3394.02 | 528.11 |
| TCGA-HT-7688-01A-11R-2256-07 | OD | 32.13 | 0 | 33.34 | 2793.63 | 247.56 | 2659.18 | 823.83 | 2540.06 | 2102.06 | 1004.71 |
| TCGA-EZ-7264-01A-11R-2027-07 | OD | 40.03 | 0 | 51.18 | 2957.70 | 265.68 | 6090.13 | 702.51 | 490.53 | 1593.79 | 1356.92 |
| TCGA-DB-A64V-01A-11R-A29R-07 | OD | 29.63 | 0 | 10.79 | 2697.74 | 1877.20 | 298.26 | 913.35 | 7101.76 | 2556.60 | 1246.57 |
| TCGA-HT-7693-01A-11R-2256-07 | OD | 17.77 | 0 | 135.33 | 2880.06 | 1069.71 | 2044.38 | 2285.85 | 33.28 | 1628.79 | 468.49 |
| TCGA-DU-8165-01A-11R-2256-07 | OD | 19.40 | 0 | 513.14 | 3795.42 | 1987.61 | 798.42 | 1440.69 | 903.90 | 4884.76 | 887.76 |
| TCGA-HW-7491-01A-11R-2027-07 | OD | 69.27 | 0 | 111.35 | 3080.18 | 325.36 | 8146.11 | 1025.66 | 1878.79 | 2069.31 | 694.79 |
| TCGA-HT-7882-01A-11R-2403-07 | OD | 3.77 | 1 | 110.57 | 3039.31 | 6280.25 | 1440.46 | 1825.73 | 57.01 | 5119.85 | 1253.37 |
| TCGA-HW-7486-01A-11R-2027-07 | OD | 57.37 | 0 | 36.25 | 2787.85 | 638.95 | 4471.19 | 772.92 | 27087.42 | 2665.96 | 1944.56 |
| TCGA-HT-7695-01A-11R-2256-07 | OD | 14.73 | 0 | 47.96 | 2589.61 | 277.84 | 3881.10 | 743.95 | 4268.58 | 1874.08 | 2080.62 |
| TCGA-TQ-A7RW-01A-11R-A33Z-07 | OD | 27.37 | 1 | 157.39 | 2579.45 | 1389.47 | 3912.14 | 1192.48 | 1904.26 | 1885.71 | 585.96 |
| TCGA-FG-5964-01A-11R-1708-07 | OD | 52.93 | 0 | 76.99 | 2407.04 | 907.45 | 980.32 | 762.08 | 3071.25 | 1520.48 | 538.90 |
| TCGA-QH-A65V-01A-11R-A29R-07 | OD | 12.80 | 0 | 39.94 | 3185.92 | 422.73 | 335.24 | 1205.42 | 8175.94 | 1411.79 | 682.83 |
| TCGA-DH-5144-01A-01R-1470-07 | OD | 24.93 | 0 | 38.97 | 3657.48 | 301.65 | 2549.74 | 900.51 | 89.67 | 1554.50 | 1988.28 |
| TCGA-E1-A7YQ-01A-11R-A34R-07 | OD | 52.60 | 1 | 144.13 | 2838.09 | 1715.08 | 26504.46 | 1378.66 | 12.97 | 7399.04 | 365.32 |
| TCGA-DU-7309-01A-11R-2090-07 | OD | 2.80 | 0 | 68.63 | 1879.25 | 504.69 | 3142.09 | 622.53 | 385.24 | 2167.37 | 1299.77 |
| TCGA-S9-A7J1-01A-21R-A34R-07 | OD | 8.30 | 0 | 66.44 | 2519.99 | 644.44 | 2439.66 | 780.89 | 6428.52 | 1873.51 | 2227.67 |
| TCGA-HT-7472-01A-11R-2027-07 | OD | 0.03 | 0 | 110.45 | 3049.45 | 1455.28 | 3303.47 | 654.34 | 5001.76 | 2767.30 | 601.79 |
| TCGA-HT-7687-01A-11R-2256-07 | OD | 0.10 | 0 | 58.52 | 3107.38 | 584.45 | 4520.94 | 911.79 | 1350.12 | 1774.28 | 1215.71 |
| TCGA-HT-7877-01A-11R-2403-07 | OD | 0.13 | 0 | 100.59 | 2398.01 | 248.68 | 3683.60 | 991.00 | 4644.52 | 1411.36 | 1899.41 |
| TCGA-VM-A8C8-01A-11R-A36H-07 | OD | 46.57 | 0 | 34.79 | 2465.53 | 729.59 | 1194.32 | 1495.00 | 2630.66 | 1275.35 | 129.82 |
| TCGA-DU-A6S8-01A-12R-A32Q-07 | OD | 22.60 | 0 | 21.49 | 3151.75 | 246.93 | 1309.63 | 971.49 | 600.44 | 1336.84 | 512.72 |
| TCGA-HT-7689-01A-11R-2256-07 | OD | 15.17 | 0 | 174.64 | 3355.23 | 684.26 | 3140.75 | 1314.72 | 7088.34 | 2630.47 | 847.32 |
| TCGA-W9-A837-01A-11R-A36H-07 | OD | 51.77 | 0 | 34.82 | 2343.11 | 162.76 | 4170.71 | 896.26 | 636.36 | 1317.82 | 2142.60 |
| TCGA-HT-A4DV-01A-11R-A26U-07 | OD | 26.40 | 0 | 7.20 | 1853.60 | 284.00 | 372.00 | 746.40 | 15473.60 | 1277.60 | 1064.00 |
| TCGA-S9-A6WD-01A-12R-A33Z-07 | OD | 76.30 | 0 | 42.71 | 2957.70 | 320.33 | 2847.94 | 1073.92 | 3676.39 | 1855.85 | 1539.63 |
| TCGA-CS-6670-01A-11R-1896-07 | OD | 47.53 | 0 | 41.71 | 2351.75 | 86.06 | 3176.25 | 891.77 | 1048.85 | 1570.84 | 1721.16 |
| TCGA-R8-A6ML-01A-11R-A32Q-07 | OD | 95.33 | 0 | 31.98 | 2949.99 | 341.70 | 2627.97 | 809.44 | 1638.59 | 1826.36 | 1140.73 |
| TCGA-DU-7294-01A-11R-2027-07 | OD | 95.63 | 0 | 74.76 | 3333.89 | 334.56 | 3334.87 | 802.55 | 3346.97 | 2144.82 | 1916.19 |
| TCGA-FG-A6IZ-01A-11R-A31N-07 | OD | 15.23 | 0 | 1.30 | 3191.79 | 290.71 | 479.48 | 1133.91 | 353.78 | 1230.24 | 400.43 |
| TCGA-HT-7470-01A-12R-2090-07 | OD | 40.67 | 1 | 105.98 | 2385.87 | 459.57 | 1951.96 | 738.66 | 7354.48 | 1919.55 | 1133.68 |
| TCGA-FG-7638-01B-12R-2090-07 | OD | 22.87 | 0 | 60.71 | 2889.85 | 261.06 | 1483.94 | 853.43 | 3329.58 | 1472.68 | 2028.62 |
| TCGA-S9-A6TW-01A-12R-A32Q-07 | OD | 41.67 | 0 | 69.30 | 3393.05 | 411.84 | 1857.62 | 1009.46 | 472.29 | 1363.31 | 367.12 |
| TCGA-DU-5874-01A-11R-1708-07 | OD | 15.37 | 0 | 108.88 | 2812.52 | 202.62 | 5036.36 | 931.59 | 64.92 | 1655.31 | 929.84 |
| TCGA-S9-A7J3-01A-21R-A34R-07 | OD | 20.97 | 0 | 59.55 | 2164.69 | 464.58 | 2850.42 | 857.53 | 3398.20 | 2233.44 | 1465.95 |
| TCGA-RY-A83Y-01A-11R-A36H-07 | OD | 5.53 | 0 | 12.49 | 2765.75 | 584.07 | 1057.23 | 874.54 | 688.18 | 1286.31 | 759.50 |
| TCGA-P5-A5F4-01A-11R-A28M-07 | OD | 7.70 | 0 | 26.40 | 3109.24 | 156.58 | 1556.19 | 812.47 | 1839.78 | 1204.82 | 723.26 |
| TCGA-R8-A6MK-01A-11R-A32Q-07 | OD | 90.07 | 0 | 82.60 | 3182.36 | 287.31 | 2068.56 | 1137.27 | 1955.31 | 1369.11 | 943.74 |
| TCGA-S9-A6TY-01A-12R-A32Q-07 | OD | 35.87 | 0 | 69.05 | 4620.23 | 173.44 | 4242.38 | 886.27 | 2042.24 | 1609.67 | 764.01 |
| TCGA-CS-6668-01A-11R-1896-07 | OD | 50.63 | 0 | 64.42 | 2706.09 | 288.03 | 8562.72 | 589.35 | 577.76 | 1662.38 | 1347.76 |
| TCGA-DU-6397-01A-11R-1708-07 | OD | 46.70 | 1 | 41.79 | 2106.55 | 650.23 | 6325.15 | 631.02 | 1670.18 | 1497.36 | 1257.15 |
| TCGA-DH-A66F-01A-11R-A29R-07 | OD | 17.43 | 0 | 17.51 | 3104.59 | 496.45 | 1696.17 | 1104.59 | 1400.38 | 1541.41 | 2048.75 |
| TCGA-HT-A615-01A-11R-A29R-07 | OD | 17.17 | 0 | 30.55 | 3172.25 | 1611.79 | 1892.23 | 961.39 | 1873.99 | 3278.32 | 1284.26 |
| TCGA-DU-6400-01A-12R-1708-07 | OD | 1.23 | 1 | 77.22 | 2539.98 | 341.18 | 3291.31 | 710.30 | 168.67 | 2203.72 | 835.71 |
| TCGA-QH-A6X9-01A-12R-A32Q-07 | OD | 1.93 | 0 | 75.12 | 3514.71 | 359.30 | 4205.72 | 1854.13 | 573.93 | 1260.33 | 943.16 |
| TCGA-E1-A7YO-01A-11R-A34F-07 | OD | 76.07 | 1 | 78.32 | 2259.18 | 100.64 | 7116.27 | 889.35 | 355.40 | 1394.25 | 2009.05 |
| TCGA-DH-A7UR-01A-11R-A33Z-07 | OD | 55.00 | 0 | 73.46 | 3019.35 | 143.36 | 1820.24 | 764.61 | 569.12 | 1133.49 | 549.37 |
| TCGA-DU-7015-01A-11R-2027-07 | OD | 92.03 | 0 | 188.04 | 4216.46 | 1459.58 | 6053.28 | 864.67 | 15.40 | 2657.68 | 446.55 |
| TCGA-FG-A711-01A-21R-A33Z-07 | OD | 49.37 | 1 | 16.23 | 2660.53 | 375.00 | 994.29 | 1079.39 | 1213.60 | 1103.95 | 493.42 |
| TCGA-DU-8168-01A-11R-2256-07 | OD | 14.37 | 0 | 196.27 | 3815.45 | 334.86 | 9229.60 | 805.96 | 88.17 | 2327.82 | 311.41 |
| TCGA-FG-6690-01A-11R-1896-07 | OD | 43.13 | 0 | 79.93 | 2431.40 | 1077.87 | 6332.89 | 944.79 | 512.57 | 1281.83 | 412.44 |
| TCGA-S9-A6WE-01A-12R-A33Z-07 | OD | 137.10 | 0 | 26.97 | 3505.09 | 436.96 | 1087.34 | 830.76 | 1888.08 | 1421.91 | 1154.43 |
| TCGA-DU-6393-01A-11R-1708-07 | OD | 52.83 | 1 | 193.52 | 2687.25 | 134.87 | 15924.34 | 783.10 | 789.56 | 1971.58 | 543.86 |
| TCGA-TQ-A7RQ-01A-11R-A33Z-07 | OD | 26.50 | 0 | 83.39 | 2504.59 | 124.59 | 6136.70 | 650.43 | 58.21 | 1281.89 | 1233.16 |
| TCGA-DU-5870-01A-11R-1708-07 | OD | 184.87 | 0 | 61.06 | 2560.06 | 572.78 | 4796.99 | 694.89 | 1905.51 | 1970.93 | 2963.47 |
| TCGA-DU-6394-01A-11R-1708-07 | OD | 22.73 | 1 | 97.52 | 3287.48 | 363.40 | 3552.01 | 844.40 | 6.34 | 2922.76 | 654.38 |
| TCGA-S9-A7R1-01A-12R-A34R-07 | OD | 172.20 | 1 | 44.63 | 3295.80 | 120.91 | 6568.15 | 1360.66 | 52.93 | 1380.38 | 884.28 |
| TCGA-RY-A847-01A-11R-A36H-07 | OD | 31.10 | 0 | 25.86 | 2727.00 | 796.62 | 3149.64 | 820.98 | 2254.10 | 2806.56 | 2100.94 |
| TCGA-DU-7301-01A-11R-2090-07 | OD | 26.27 | 1 | 225.88 | 2903.89 | 1153.37 | 2884.79 | 820.61 | 3689.80 | 3806.38 | 1381.68 |
| TCGA-R8-A6MO-01A-11R-A33Z-07 | OD | 33.10 | 0 | 24.72 | 3003.66 | 560.27 | 1051.81 | 927.98 | 7419.29 | 1736.95 | 1017.09 |
| TCGA-QH-A65Z-01A-11R-A29R-07 | OD | 14.23 | 0 | 24.92 | 3338.43 | 210.76 | 1085.25 | 887.19 | 3267.60 | 1434.63 | 1046.35 |
| TCGA-E1-5311-01A-01R-1470-07 | OD | 136.13 | 1 | 147.28 | 2365.73 | 150.06 | 6957.26 | 748.44 | 453.57 | 1696.64 | 926.59 |
| TCGA-HT-7471-01A-11R-2256-07 | OD | 0.13 | 0 | 147.13 | 2702.64 | 1078.80 | 5611.77 | 739.91 | 612.97 | 6840.06 | 1357.92 |
| TCGA-DU-A76K-01A-11R-A33Z-07 | OD | 11.57 | 1 | 31.04 | 3145.42 | 173.96 | 1493.78 | 1363.83 | 1196.02 | 2136.61 | 482.74 |
| TCGA-P5-A5F0-01A-11R-A28M-07 | OD | 9.57 | 0 | 41.56 | 3505.31 | 122.88 | 4163.46 | 831.71 | 673.14 | 1427.60 | 2177.10 |
| TCGA-HT-7676-01A-11R-2403-07 | OD | 0.17 | 0 | 63.63 | 3452.58 | 1294.52 | 1796.29 | 768.71 | 2529.81 | 1832.73 | 302.92 |
| TCGA-DU-5849-01A-11R-1708-07 | OD | 14.77 | 0 | 67.85 | 2463.11 | 483.13 | 3324.94 | 936.14 | 3587.88 | 1826.04 | 1149.72 |
| TCGA-VM-A8CB-01A-11R-A36H-07 | OD | 0.10 | 0 | 9.48 | 3402.30 | 169.21 | 1913.92 | 915.27 | 135.56 | 1400.17 | 88.16 |
| TCGA-DB-A64P-01A-11R-A29R-07 | OD | 30.53 | 0 | 69.08 | 3528.64 | 720.72 | 1896.36 | 899.33 | 1897.64 | 2250.63 | 809.18 |
| TCGA-TQ-A7RF-01A-11R-A33Z-07 | OD | 21.67 | 0 | 185.47 | 3101.99 | 551.90 | 2112.28 | 1597.92 | 18.50 | 1125.45 | 107.85 |
| TCGA-TM-A84J-01A-11R-A36H-07 | OD | 24.50 | 0 | 210.68 | 4099.71 | 201.08 | 89.70 | 1014.60 | 36.71 | 3049.65 | 196.50 |
| TCGA-VW-A7QS-01A-12R-A33Z-07 | OD | 23.53 | 0 | 68.21 | 3098.95 | 138.74 | 3133.87 | 1817.13 | 0.00 | 1930.05 | 512.57 |
| TCGA-TQ-A7RI-01A-11R-A33Z-07 | OD | 2.07 | 0 | 57.15 | 3237.38 | 606.59 | 5121.55 | 1151.44 | 4319.98 | 2253.65 | 2190.24 |
| TCGA-S9-A6UB-01A-21R-A33Z-07 | OD | 125.37 | 0 | 33.62 | 2501.54 | 132.72 | 3797.65 | 784.52 | 3467.67 | 934.70 | 1322.81 |
| TCGA-DB-A4XG-01A-11R-A27Q-07 | OD | 73.97 | 0 | 85.11 | 3124.41 | 100.61 | 8595.34 | 958.17 | 25.96 | 1598.99 | 1450.05 |
| TCGA-TM-A84S-01A-11R-A36H-07 | OD | 15.13 | 0 | 60.30 | 3290.04 | 607.96 | 2436.77 | 997.39 | 2856.62 | 1847.44 | 1274.40 |
| TCGA-DU-7008-01A-11R-2027-07 | OD | 158.40 | 0 | 100.47 | 3876.24 | 240.19 | 1927.24 | 934.33 | 68.29 | 1928.31 | 360.81 |
| TCGA-DB-5278-01A-01R-1470-07 | OD | 92.40 | 0 | 37.81 | 2716.36 | 459.60 | 994.18 | 624.47 | 2504.07 | 1533.12 | 2069.88 |
| TCGA-F6-A8O3-01A-11R-A36H-07 | OD | 0.23 | 0 | 57.51 | 3002.43 | 151.46 | 4028.82 | 843.95 | 98.00 | 1819.11 | 446.81 |
| TCGA-FG-7634-01A-11R-2090-07 | OD | 15.57 | 0 | 159.58 | 3103.17 | 1255.20 | 4830.14 | 599.10 | 6229.86 | 2522.78 | 1976.47 |
| TCGA-P5-A72U-01A-31R-A32Q-07 | OD | 0.23 | 0 | 34.27 | 2006.55 | 829.64 | 1149.16 | 458.17 | 24.19 | 1582.16 | 247.48 |
| TCGA-S9-A6U2-01A-21R-A33Z-07 | OD | 30.27 | 0 | 85.66 | 2584.48 | 456.22 | 4006.04 | 912.92 | 147.66 | 1698.53 | 2155.70 |
| TCGA-TM-A84O-01A-11R-A36H-07 | OD | 33.70 | 1 | 31.46 | 2947.10 | 605.11 | 479.18 | 829.00 | 657.55 | 1580.03 | 572.73 |
| TCGA-S9-A7QZ-01A-12R-A34R-07 | OD | 27.53 | 0 | 66.62 | 2470.98 | 112.87 | 3237.79 | 733.26 | 1222.76 | 1089.51 | 980.16 |
| TCGA-TQ-A7RU-01A-21R-A34F-07 | OD | 34.40 | 0 | 33.33 | 2823.21 | 184.39 | 1270.37 | 762.87 | 459.92 | 1295.78 | 748.52 |
| TCGA-DU-A7T6-01A-11R-A33Z-07 | OD | 18.23 | 1 | 43.85 | 4732.44 | 278.08 | 102.69 | 744.80 | 1741.78 | 1188.55 | 937.25 |
| TCGA-TM-A84G-01A-11R-A36H-07 | OD | 41.20 | 0 | 66.31 | 2786.66 | 97.76 | 2583.49 | 642.67 | 23.49 | 2158.77 | 1298.98 |
| TCGA-DH-5141-01A-01R-1470-07 | OD | 32.27 | 0 | 66.20 | 3024.73 | 475.41 | 2164.17 | 591.26 | 2006.96 | 2111.52 | 1271.65 |
| TCGA-DU-7014-01A-11R-2027-07 | OD | 119.03 | 1 | 93.56 | 4338.28 | 469.35 | 3197.39 | 1215.71 | 671.51 | 1399.77 | 229.46 |
| TCGA-QH-A65R-01A-21R-A31N-07 | OD | 15.37 | 0 | 37.73 | 3573.80 | 326.70 | 2018.22 | 1001.66 | 69.24 | 1078.36 | 653.81 |
| TCGA-DB-A64R-01A-11R-A29R-07 | OD | 31.83 | 0 | 50.39 | 3142.44 | 191.86 | 2312.49 | 677.81 | 309.11 | 2150.68 | 1477.23 |
| TCGA-QH-A86X-01A-11R-A36H-07 | OD | 11.23 | 0 | 89.19 | 2687.86 | 342.35 | 3394.65 | 821.81 | 379.19 | 1287.15 | 1982.66 |
| TCGA-FG-A70Y-01A-12R-A34R-07 | OD | 28.93 | 0 | 68.68 | 2805.70 | 738.27 | 1417.46 | 896.15 | 8082.91 | 1008.79 | 394.89 |
| TCGA-FG-A710-01A-12R-A33Z-07 | OD | 37.17 | 0 | 61.12 | 2492.45 | 1231.82 | 1303.12 | 784.69 | 484.37 | 4650.16 | 1276.78 |
| TCGA-DU-A7TA-01A-11R-A33Z-07 | OD | 85.50 | 0 | 92.73 | 5280.27 | 971.52 | 2767.60 | 2177.06 | 5.57 | 4057.61 | 85.57 |
| TCGA-HT-8108-01A-11R-2404-07 | OD | 2.53 | 0 | 104.82 | 3232.94 | 1246.98 | 3575.46 | 1391.11 | 1509.41 | 5099.11 | 347.40 |
| TCGA-RY-A840-01A-11R-A36H-07 | OD | 28.47 | 0 | 19.21 | 3121.09 | 222.55 | 1760.28 | 1188.73 | 6.68 | 1331.94 | 1703.55 |
| TCGA-FG-A6J1-01A-11R-A31N-07 | OD | 16.67 | 0 | 3.53 | 2872.98 | 382.06 | 263.08 | 834.17 | 150.20 | 1154.74 | 757.06 |
| TCGA-FG-8182-01A-11R-2256-07 | OD | 13.87 | 0 | 82.17 | 3074.90 | 963.11 | 11214.48 | 1085.34 | 8140.72 | 1393.48 | 1129.85 |
| TCGA-QH-A6X8-01A-12R-A32Q-07 | OD | 16.97 | 0 | 71.86 | 2459.55 | 343.42 | 2104.15 | 945.31 | 5617.47 | 1413.40 | 389.08 |
| TCGA-DU-6410-01A-11R-1896-07 | OD | 8.07 | 0 | 80.36 | 4824.14 | 88.30 | 21371.39 | 1348.00 | 93.26 | 2027.95 | 753.70 |
| TCGA-DU-7009-01A-11R-2027-07 | OD | 156.50 | 1 | 85.94 | 3202.82 | 223.26 | 10879.47 | 1321.21 | 5559.50 | 2298.98 | 1429.39 |
| TCGA-HT-7468-01A-11R-2027-07 | OD | 6.77 | 0 | 159.54 | 3297.52 | 479.25 | 4647.74 | 834.03 | 1416.21 | 2233.84 | 710.20 |
| TCGA-HT-A5R9-01A-11R-A28M-07 | OD | 0.07 | 0 | 120.35 | 4843.76 | 233.04 | 4533.61 | 1372.99 | 35.16 | 1400.03 | 933.95 |
| TCGA-HT-7480-01A-11R-2090-07 | OD | 76.23 | 0 | 70.07 | 2556.52 | 225.02 | 3811.78 | 721.59 | 1815.30 | 1937.93 | 1335.95 |
| TCGA-R8-A73M-01A-11R-A32Q-07 | OD | 60.20 | 0 | 3.27 | 2651.61 | 142.62 | 308.63 | 795.86 | 143.17 | 1154.06 | 1018.51 |
| TCGA-DU-7018-01A-11R-2027-07 | OD | 31.10 | 1 | 95.15 | 3431.32 | 137.86 | 5277.18 | 851.65 | 16.48 | 2421.83 | 642.86 |
| TCGA-HT-7677-01A-11R-2256-07 | OD | 16.47 | 0 | 57.40 | 3029.14 | 333.04 | 6202.74 | 841.78 | 4168.71 | 1557.98 | 1451.63 |
| TCGA-HT-7875-01A-11R-2403-07 | OD | 0.33 | 0 | 69.27 | 3044.12 | 150.91 | 3372.81 | 796.90 | 535.91 | 1520.82 | 1538.76 |
| TCGA-HT-8012-01A-11R-2403-07 | OD | 9.53 | 0 | 63.44 | 2289.38 | 361.88 | 2939.96 | 665.94 | 677.50 | 1222.81 | 1448.75 |
| TCGA-DH-A669-01A-12R-A31N-07 | OD | 30.63 | 1 | 98.85 | 4331.98 | 766.12 | 439.07 | 1302.40 | 193.59 | 13457.82 | 438.80 |
| TCGA-HW-A5KJ-01A-12R-A27Q-07 | OD | 32.07 | 1 | 8.45 | 4499.75 | 148.19 | 908.00 | 1361.01 | 60.17 | 1199.90 | 739.43 |
| TCGA-HT-A619-01A-11R-A29R-07 | OD | 21.70 | 0 | 82.85 | 3947.03 | 162.08 | 2490.54 | 1738.09 | 1.36 | 1387.21 | 178.83 |
| TCGA-RY-A83X-01A-11R-A36H-07 | OD | 31.30 | 0 | 52.60 | 2469.91 | 279.98 | 1336.61 | 682.15 | 5833.71 | 1186.18 | 1874.32 |
| TCGA-CS-5395-01A-01R-1470-07 | OD | 21.30 | 1 | 213.64 | 3595.93 | 525.39 | 11307.96 | 1071.15 | 3441.16 | 2285.59 | 3090.90 |
| TCGA-E1-A7YS-01A-11R-A34F-07 | OD | 15.53 | 1 | 256.57 | 2930.29 | 153.28 | 10766.49 | 1105.84 | 157.66 | 3860.95 | 298.54 |
| TCGA-TQ-A8XE-01A-11R-A36H-07 | OD | 31.80 | 1 | 58.57 | 2396.05 | 384.70 | 3212.98 | 1020.13 | 666.91 | 1507.69 | 80.16 |
| TCGA-HT-A74K-01A-11R-A32Q-07 | OD | 15.57 | 0 | 1.77 | 2286.30 | 369.54 | 280.99 | 742.62 | 3847.70 | 920.90 | 325.27 |
| TCGA-DH-A7US-01A-11R-A33Z-07 | OD | 23.53 | 0 | 41.97 | 2746.17 | 375.30 | 1652.51 | 804.68 | 8601.69 | 1612.59 | 797.82 |
| TCGA-DU-6399-01A-12R-1708-07 | OD | 66.67 | 1 | 154.35 | 2903.49 | 857.12 | 6416.62 | 913.37 | 1336.90 | 3089.31 | 441.28 |
| TCGA-VM-A8CE-01A-11R-A36H-07 | OD | 39.70 | 0 | 71.26 | 2447.56 | 339.87 | 1080.45 | 1003.20 | 8122.90 | 1237.79 | 1337.87 |
| TCGA-IK-7675-01A-11R-2090-07 | OD | 19.27 | 1 | 206.05 | 2624.65 | 368.28 | 2709.41 | 965.16 | 1569.65 | 1509.64 | 83.82 |
| TCGA-DU-A5TS-01A-11R-A28M-07 | OD | 32.13 | 0 | 28.52 | 3191.44 | 454.50 | 182.30 | 1038.60 | 10051.77 | 1194.85 | 272.83 |
| TCGA-P5-A72Z-01A-11R-A32Q-07 | OD | 11.80 | 0 | 46.82 | 2388.85 | 75.39 | 1003.84 | 569.41 | 88.13 | 937.09 | 132.89 |
| TCGA-TM-A84M-01A-11R-A36H-07 | OD | 25.13 | 0 | 95.99 | 3674.39 | 204.80 | 7531.44 | 1010.34 | 86.88 | 1390.57 | 2489.04 |
| TCGA-HT-A61C-01A-11R-A29R-07 | OD | 17.90 | 1 | 88.77 | 6449.10 | 796.96 | 52.21 | 1676.00 | 1648.12 | 4631.30 | 350.75 |
| TCGA-S9-A6TX-01A-21R-A32Q-07 | OD | 41.50 | 0 | 57.35 | 2329.78 | 278.16 | 3488.61 | 770.18 | 6607.13 | 1124.13 | 684.97 |
| TCGA-DU-6408-01A-11R-1708-07 | OD | 115.67 | 1 | 105.46 | 3093.91 | 692.41 | 2039.69 | 1113.85 | 171.42 | 2339.49 | 381.16 |
| TCGA-TQ-A7RN-01A-11R-A33Z-07 | OD | 34.20 | 0 | 56.00 | 3104.14 | 207.01 | 8783.40 | 928.69 | 1064.29 | 1644.72 | 1279.16 |
| TCGA-DU-A6S3-01A-12R-A32Q-07 | OD | 21.87 | 0 | 28.03 | 2483.98 | 111.11 | 2681.62 | 590.09 | 2953.45 | 1051.55 | 1038.04 |
| TCGA-E1-5318-01A-01R-1470-07 | OD | 79.30 | 1 | 106.79 | 2953.37 | 335.42 | 3427.88 | 629.86 | 31.21 | 1519.55 | 561.79 |
| TCGA-FG-6692-01A-11R-1896-07 | OD | 18.70 | 1 | 84.89 | 4992.12 | 574.61 | 14585.90 | 2368.21 | 314.80 | 6251.32 | 439.89 |
| TCGA-S9-A7J2-01A-11R-A34F-07 | OD | 2.07 | 0 | 125.35 | 2603.68 | 150.11 | 5305.95 | 878.94 | 129.92 | 1302.98 | 1694.26 |
| TCGA-DU-8164-01A-11R-2256-07 | OD | 21.70 | 0 | 81.15 | 3117.34 | 262.99 | 4751.84 | 656.49 | 481.63 | 1616.68 | 2191.73 |
| TCGA-DU-6401-01A-11R-1708-07 | OD | 88.67 | 1 | 200.81 | 3144.32 | 314.19 | 5989.53 | 1034.00 | 117.77 | 2267.61 | 154.50 |
| TCGA-DU-6404-01A-11R-1708-07 | OD | 135.60 | 1 | 179.03 | 2870.34 | 409.93 | 5411.26 | 588.41 | 13.24 | 3305.93 | 685.24 |
| TCGA-FG-A4MT-01A-11R-A26U-07 | OD | 38.80 | 0 | 19.00 | 3147.72 | 406.81 | 3302.31 | 797.25 | 265.31 | 1070.42 | 76.65 |
| TCGA-HW-8322-01A-11R-2404-07 | OD | 25.27 | 0 | 134.93 | 3278.04 | 654.21 | 3809.24 | 1230.43 | 2949.77 | 1496.20 | 2522.78 |
| TCGA-DU-6407-01A-13R-1708-07 | OD | 95.83 | 1 | 160.19 | 4042.80 | 557.13 | 7771.08 | 1070.67 | 1946.21 | 1381.63 | 361.21 |
| TCGA-HT-7469-01A-11R-2256-07 | OD | 11.70 | 1 | 334.47 | 3234.98 | 430.22 | 6209.69 | 1395.86 | 279.11 | 3130.43 | 57.32 |
| TCGA-S9-A7J0-01A-11R-A34F-07 | OD | 20.30 | 0 | 313.42 | 4657.83 | 337.70 | 864.48 | 1440.58 | 1413.42 | 1295.21 | 388.82 |
| TCGA-06-0675-11A-32R-A36H-07 | NG |  |  | 22.49 | 1382.82 | 384.62 | 388.17 | 554.21 | 1085.92 | 3306.81 | 2535.77 |
| TCGA-06-0678-11A-32R-A36H-07 | NG |  |  | 31.26 | 1180.31 | 252.86 | 248.19 | 742.24 | 3550.73 | 2107.30 | 976.91 |
| TCGA-06-0680-11A-32R-A36H-07 | NG |  |  | 21.31 | 1318.60 | 223.32 | 436.36 | 708.23 | 1197.47 | 2024.86 | 2033.93 |
| TCGA-06-0681-11A-41R-A36H-07 | NG |  |  | 11.11 | 1450.00 | 243.16 | 171.37 | 645.73 | 4094.87 | 1868.38 | 1727.35 |
| TCGA-06-AABW-11A-31R-A36H-07 | NG |  |  | 36.68 | 1705.53 | 293.97 | 122.10 | 1315.58 | 29128.64 | 1846.73 | 452.76 |

Abbreviations: GBM, glioblastoma; A, astrocytoma; OA, oligoastrocytoma; OD, oligodendroglioma; NG, non-glioma.

**Table S6.** Eight differentially expressed genes validation results in CGGA dataset

| **sample ID** | **Histology type** | **Overall survival (months)** | **vital state** | **BARD1** | **CBX3** | **CTSS** | **EGFR** | **GUCY1A3** | **IFRD1** | **MOBP** | **STAT1** |
| --- | --- | --- | --- | --- | --- | --- | --- | --- | --- | --- | --- |
| 784.00 | A | 19.30 | 0 | -1.26 | -0.83 | -0.91 | -1.64 | 1.25 | -1.14 | 1.99 | -0.40 |
| 412.00 | A | 9.33 | 0 | -1.56 | -0.80 | -0.75 | -2.48 | 0.48 | -1.24 | 1.73 | 0.02 |
| 542.00 | A | 29.57 | 0 | -1.40 | -0.44 | -0.87 | -2.15 | 1.80 | -1.38 | 0.61 | -0.36 |
| 433.00 | A | 33.77 | 0 | -0.80 | -0.69 | 0.15 | -1.98 | 0.52 | -0.78 | 0.51 | -0.29 |
| 353.00 | A | 17.67 | 1 | -1.29 | -0.76 | -0.69 | 1.18 | 0.42 | -0.36 | 2.53 | -0.74 |
| 626.00 | A | 26.13 | 0 | -0.70 | -0.39 | -0.10 | -1.71 | 0.15 | -0.62 | 3.17 | -0.20 |
| 692.00 | A | 22.97 | 0 | -1.27 | -0.75 | -0.20 | -1.12 | 0.89 | -0.38 | 1.50 | 0.22 |
| 595.00 | A | 27.50 | 0 | -0.89 | -0.62 | -0.66 | -0.91 | 0.77 | -1.08 | 1.93 | -0.67 |
| 552.00 | A | 29.10 | 0 | 0.53 | -0.46 | 1.18 | -3.15 | -1.22 | -0.59 | -1.54 | 0.04 |
| 673.00 | A | 14.57 | 1 | -0.56 | -0.54 | -0.20 | -2.15 | 0.23 | -0.42 | 4.29 | -0.31 |
| 461.00 | A | 33.03 | 0 | -1.03 | -0.32 | -1.15 | -0.96 | 1.32 | -1.12 | 0.71 | -0.51 |
| 718.00 | A | 21.83 | 0 | 0.49 | -0.53 | 0.58 | -0.92 | -0.69 | -0.71 | 1.74 | -0.22 |
| 365.00 | A | 8.67 | 1 | -1.10 | -0.52 | -0.99 | -0.86 | 0.83 | -0.98 | 1.47 | -0.69 |
| 770.00 | A | 19.80 | 0 | -0.79 | -0.34 | -1.12 | 0.71 | 0.26 | -0.76 | 1.37 | -0.70 |
| 271.00 | A | 41.87 | 0 | -0.62 | -0.12 | -0.60 | 0.71 | -1.21 | 0.57 | 2.88 | 0.01 |
| 688.00 | A | 23.07 | 0 | 0.15 | 0.15 | 0.82 | -2.16 | -0.66 | 0.01 | -2.42 | 0.07 |
| 321.00 | A | 39.10 | 0 | -0.04 | -0.20 | -0.16 | 1.44 | -0.65 | 0.02 | -1.58 | 0.53 |
| 258.00 | A | 38.77 | 1 | 0.04 | -0.07 | -0.02 | -1.49 | 0.96 | -0.99 | 2.18 | -0.35 |
| 663.00 | A | 24.03 | 0 | -0.39 | -0.10 | -0.66 | 0.98 | 0.78 | -1.00 | 1.17 | -0.55 |
| 746.00 | A | 20.63 | 0 | 0.26 | -0.66 | 0.21 | -0.45 | -0.15 | -0.70 | 1.70 | 0.71 |
| 610.00 | A | 26.33 | 0 | -0.47 | -0.05 | -0.30 | 0.05 | 0.29 | 0.23 | -0.09 | 0.52 |
| 415.00 | A | 34.67 | 0 | -1.07 | -1.09 | 0.19 | 0.53 | 1.67 | 0.04 | 1.78 | -0.18 |
| 331.00 | A | 43.47 | 0 | 0.22 | 0.48 | 0.19 | -1.92 | -1.01 | -0.18 | -3.65 | -0.07 |
| 522.00 | A | 30.27 | 0 | -0.42 | -0.37 | 0.08 | 0.22 | 1.18 | -0.57 | 0.74 | -0.74 |
| 711.00 | A | 22.07 | 0 | 0.61 | -0.10 | -0.39 | 0.46 | 1.02 | -0.43 | 1.03 | 0.08 |
| 440.00 | A | 33.10 | 0 | 0.82 | -0.89 | 1.13 | 1.03 | 0.53 | 0.61 | 1.20 | 0.82 |
| 354.00 | A | 4.83 | 0 | 0.08 | 0.55 | 0.03 | 2.76 | 0.15 | 0.50 | 1.19 | 0.01 |
| 434.00 | A | 33.73 | 0 | 0.18 | 0.07 | 0.13 | 0.34 | -0.60 | -0.23 | 1.60 | 0.01 |
| 792.00 | A | 18.57 | 0 | 0.04 | -0.38 | 0.06 | 0.80 | -1.19 | 0.26 | 1.35 | -0.10 |
| 285.00 | A | 40.83 | 0 | -0.11 | -0.33 | -0.97 | 1.27 | -0.48 | -0.38 | 1.18 | -0.32 |
| 469.00 | A | 32.60 | 0 | -0.21 | 0.04 | 0.17 | 0.23 | -0.10 | 0.04 | 1.81 | -0.41 |
| 402.00 | A | 35.37 | 0 | 0.00 | -0.28 | -0.59 | -0.01 | 0.11 | -0.50 | -0.05 | -0.13 |
| 566.00 | A | 28.17 | 0 | -0.19 | -0.27 | -0.34 | 0.09 | -0.69 | -0.43 | 1.26 | 0.07 |
| 253.00 | A | 55.27 | 0 | -0.13 | 0.19 | 0.30 | 0.08 | -1.72 | 0.33 | -0.22 | -0.02 |
| 601.00 | A | 27.27 | 0 | 1.19 | -0.06 | 0.22 | -0.09 | -0.08 | 0.03 | 1.98 | -0.45 |
| 476.00 | A | 36.30 | 1 | 0.08 | -0.45 | 1.56 | 4.03 | -1.32 | 0.27 | -0.05 | 0.96 |
| 648.00 | A | 25.13 | 0 | 0.09 | -0.21 | -0.36 | 1.04 | -0.76 | -0.03 | 0.06 | -0.45 |
| 395.00 | A | 35.87 | 0 | -0.12 | -0.72 | 0.01 | 0.74 | -0.04 | 0.61 | -0.49 | -0.05 |
| 505.00 | A | 30.77 | 0 | 0.06 | -0.04 | 0.79 | -0.58 | -0.49 | -0.60 | 2.20 | 1.36 |
| 441.00 | A | 33.10 | 0 | 0.14 | -0.17 | -0.84 | 0.87 | 0.29 | -0.44 | 0.53 | 0.18 |
| 407.00 | A | 35.13 | 0 | 0.46 | -0.05 | 0.39 | 0.18 | -0.78 | 0.06 | 0.89 | -0.83 |
| 379.00 | A | 36.37 | 0 | 0.09 | 0.17 | 0.39 | 0.02 | 0.30 | -0.03 | 0.58 | 0.46 |
| 447.00 | A | 33.57 | 0 | 0.36 | 0.02 | 1.06 | 0.70 | 0.04 | -0.30 | 1.22 | 1.11 |
| 281.00 | A | 41.17 | 0 | -0.10 | -0.05 | 0.00 | 0.56 | -0.14 | -0.17 | 2.56 | -0.55 |
| 316.00 | A | 39.37 | 0 | 1.07 | -0.01 | -0.04 | 1.31 | -0.59 | -0.36 | -1.07 | -0.52 |
| 767.00 | A | 19.93 | 0 | 0.99 | 0.29 | 1.20 | -0.12 | -0.47 | -0.30 | -2.97 | 0.22 |
| 590.00 | A | 27.67 | 0 | -0.01 | 0.00 | 0.26 | -0.20 | -0.53 | -0.77 | -0.75 | 0.42 |
| 396.00 | A | 12.53 | 1 | 0.54 | 0.22 | -0.86 | -0.43 | -0.14 | -0.77 | -1.11 | -0.34 |
| 459.00 | A | 32.57 | 0 | 0.05 | 0.13 | 0.61 | 0.87 | -0.97 | -0.31 | 1.45 | -0.48 |
| 592.00 | A | 27.53 | 0 | -0.94 | 0.28 | -0.40 | 0.72 | -1.57 | -0.02 | -0.73 | -0.85 |
| 736.00 | A | 20.97 | 0 | -0.10 | -0.33 | -0.55 | -0.54 | -0.18 | 0.28 | 0.14 | 0.27 |
| 571.00 | A | 28.47 | 0 | -0.51 | 0.68 | -0.60 | 0.83 | -1.17 | -0.37 | 1.02 | -0.41 |
| 249.00 | A | 10.40 | 1 | 0.34 | -0.06 | 0.11 | 4.58 | -1.73 | -0.42 | -2.71 | -0.75 |
| 776.00 | A | 19.57 | 0 | 0.00 | -0.06 | -0.01 | 0.59 | -0.60 | 0.11 | -1.90 | 0.84 |
| 743.00 | A | 20.73 | 0 | 0.45 | 0.03 | -0.66 | 0.90 | -0.93 | 0.17 | -2.15 | 0.01 |
| 708.00 | A | 22.10 | 0 | -0.92 | 0.12 | -0.24 | 0.56 | -1.16 | -0.09 | 0.40 | -0.94 |
| 544.00 | A | 29.40 | 0 | -0.17 | 0.36 | -0.28 | -0.70 | -1.09 | -0.23 | 1.83 | -0.65 |
| 583.00 | A | 28.00 | 0 | -0.11 | 0.47 | 0.02 | 0.73 | -0.65 | 0.07 | 1.24 | -0.72 |
| 334.00 | A | 43.30 | 0 | -0.50 | -0.34 | -0.46 | -0.45 | 0.04 | -0.93 | -0.02 | -0.93 |
| 405.00 | A | 16.57 | 1 | 0.32 | 0.46 | 0.08 | -0.96 | -1.24 | -0.52 | -2.97 | -0.46 |
| 399.00 | A | 35.43 | 0 | 0.32 | 0.45 | -0.12 | 1.65 | -1.84 | -0.29 | -0.76 | 0.50 |
| 753.00 | A | 20.43 | 0 | 0.36 | -0.19 | 0.11 | 0.42 | -1.00 | -0.27 | -0.83 | -0.19 |
| 712.00 | A | 22.03 | 0 | 0.71 | -0.50 | -0.07 | 1.46 | -1.31 | -0.04 | 0.22 | -0.60 |
| 357.00 | A | 37.73 | 0 | -0.51 | 0.35 | -0.70 | 1.12 | -0.62 | 0.67 | -1.89 | 0.05 |
| 548.00 | A | 29.17 | 0 | -0.53 | 0.17 | -0.12 | 0.99 | -0.60 | -0.55 | -2.97 | -0.17 |
| 317.00 | A | 39.33 | 0 | 0.04 | 0.05 | -0.61 | 1.14 | -1.24 | 0.20 | -3.14 | -0.72 |
| 628.00 | GBM | 0.00 | 0 | 0.31 | 0.48 | 2.12 | -2.85 | -0.36 | -0.26 | -0.96 | 1.49 |
| 444.00 | GBM | 7.50 | 1 | -1.03 | -0.08 | -1.09 | -1.96 | 0.53 | -0.59 | 2.45 | -0.60 |
| 530.00 | GBM | 16.87 | 1 | -1.21 | -0.73 | 0.34 | 0.18 | 0.65 | 0.12 | 3.01 | 0.46 |
| 462.00 | GBM | 12.13 | 1 | -1.84 | -0.62 | -0.52 | -2.12 | 0.29 | -0.98 | 2.16 | 0.01 |
| 649.00 | GBM | 4.90 | 1 | -0.54 | 0.36 | -0.26 | -1.71 | 0.57 | -0.06 | 1.69 | -0.41 |
| 308.00 | GBM | 25.23 | 0 | -1.12 | -0.75 | -0.20 | -0.96 | 1.53 | 0.26 | 2.85 | 0.54 |
| 719.00 | GBM | 2.00 | 1 | -0.18 | -0.39 | -0.30 | -1.44 | 0.81 | 0.10 | 0.97 | -0.12 |
| 168.00 | GBM | 33.17 | 0 | -0.83 | -0.05 | 1.14 | -2.93 | 0.83 | 0.07 | -0.48 | 1.16 |
| 594.00 | GBM | 17.73 | 0 | -0.60 | -0.16 | 0.62 | -1.99 | 0.52 | -0.16 | 1.41 | 0.25 |
| 680.00 | GBM | 23.80 | 0 | 0.90 | 0.46 | 0.43 | -1.24 | -0.86 | -0.32 | -2.04 | -0.24 |
| 700.00 | GBM | 22.30 | 0 | 0.78 | 0.30 | 1.31 | -1.18 | -1.44 | 0.00 | -2.27 | 0.61 |
| 370.00 | GBM | 11.27 | 1 | -0.27 | -0.13 | -0.40 | -0.41 | -0.31 | 0.12 | 2.57 | 0.19 |
| 512.00 | GBM | 19.60 | 0 | -0.59 | -0.54 | 0.33 | -2.40 | 1.15 | -0.02 | 3.63 | -0.32 |
| 178.00 | GBM | 32.20 | 0 | -0.17 | -0.09 | 0.98 | 1.72 | -0.22 | 0.18 | -4.57 | 1.59 |
| 464.00 | GBM | 13.43 | 1 | 0.48 | -0.01 | 0.02 | -1.08 | -0.57 | -0.54 | -3.84 | -0.35 |
| 375.00 | GBM | 21.90 | 1 | 0.02 | 0.05 | 1.08 | 1.26 | -0.46 | -0.23 | -2.86 | -0.04 |
| 504.00 | GBM | 18.77 | 1 | 0.26 | -0.29 | 0.76 | -1.25 | -0.72 | -0.10 | -1.42 | 0.95 |
| 693.00 | GBM | 22.70 | 0 | 0.37 | -0.26 | 1.44 | -2.52 | 0.64 | -0.14 | 1.45 | 0.41 |
| 255.00 | GBM | 19.70 | 1 | -0.37 | -0.20 | 1.99 | -2.10 | 0.04 | 0.37 | -3.17 | 1.89 |
| 126.00 | GBM | 26.53 | 0 | -0.87 | -0.08 | 0.20 | -0.64 | -1.26 | -1.10 | -2.57 | -0.57 |
| 575.00 | GBM | 18.47 | 0 | -1.04 | -0.11 | 1.96 | -0.43 | 0.06 | 0.77 | -1.50 | 2.07 |
| 287.00 | GBM | 18.77 | 1 | -1.15 | 0.45 | 1.33 | -2.43 | -0.50 | 0.27 | -2.39 | -0.24 |
| 205.00 | GBM | 9.73 | 1 | 1.46 | -0.18 | 1.33 | -0.44 | 0.54 | 0.10 | -1.33 | 0.38 |
| 646.00 | GBM | 12.10 | 1 | -0.36 | 0.06 | 0.66 | -2.30 | 0.08 | -0.30 | 0.19 | -0.30 |
| 741.00 | GBM | 13.20 | 1 | 0.38 | 0.26 | 0.51 | 2.55 | -0.18 | 0.37 | 1.07 | -0.31 |
| 549.00 | GBM | 13.77 | 1 | -0.03 | -0.65 | -0.14 | -0.56 | 1.12 | 0.44 | 3.37 | 0.06 |
| 734.00 | GBM | 16.00 | 0 | -0.05 | 0.67 | 0.91 | 3.26 | -1.87 | 0.02 | -1.67 | 0.15 |
| 221.00 | GBM | 9.57 | 1 | -0.29 | 0.22 | 2.61 | -1.92 | 1.11 | 0.25 | -2.39 | 0.87 |
| 124.00 | GBM | 13.80 | 1 | -0.84 | 0.06 | -0.11 | 1.70 | -1.72 | 0.43 | -0.28 | -0.11 |
| 612.00 | GBM | 16.80 | 0 | 0.25 | 0.53 | 0.24 | 0.35 | -0.43 | 0.93 | -0.98 | 3.14 |
| 609.00 | GBM | 17.07 | 0 | -0.26 | 0.14 | -0.07 | 1.25 | -1.20 | 1.62 | -1.90 | 1.40 |
| 570.00 | GBM | 18.70 | 0 | 0.60 | -0.03 | 1.23 | -2.43 | 0.17 | -0.61 | 2.98 | 1.69 |
| 195.00 | GBM | 15.17 | 1 | 0.13 | 0.03 | 0.57 | 0.89 | -0.73 | 1.00 | -3.23 | 1.91 |
| 778.00 | GBM | 5.83 | 1 | -0.04 | 0.05 | -0.24 | 0.51 | 0.52 | -0.27 | 1.31 | -0.49 |
| 451.00 | GBM | 18.60 | 0 | 0.14 | 0.58 | 1.26 | 5.09 | 0.29 | 0.43 | -3.07 | -0.07 |
| 324.00 | GBM | 11.20 | 1 | 0.11 | 0.20 | 1.08 | -3.34 | -0.41 | -0.05 | 2.06 | 1.12 |
| 437.00 | GBM | 23.13 | 0 | 0.25 | 0.60 | 0.66 | -0.13 | -0.50 | 0.17 | 2.11 | 0.70 |
| 729.00 | GBM | 23.67 | 0 | 0.76 | 0.10 | 1.09 | -2.17 | -1.04 | -0.28 | -3.55 | 0.86 |
| 419.00 | GBM | 6.60 | 1 | 0.72 | 0.51 | 1.15 | -2.52 | -1.15 | 0.94 | -4.13 | -0.14 |
| 699.00 | GBM | 19.47 | 1 | 0.29 | 0.18 | 1.66 | 3.17 | -0.80 | 0.41 | -4.24 | -0.05 |
| 709.00 | GBM | 14.00 | 1 | 1.43 | 0.74 | 0.97 | 4.93 | -1.50 | 0.04 | -0.69 | -0.18 |
| 547.00 | GBM | 19.33 | 0 | -0.16 | 0.14 | 1.21 | 0.16 | 0.51 | 0.60 | 1.29 | 0.60 |
| 311.00 | GBM | 7.67 | 1 | -1.00 | -0.33 | -0.57 | -0.11 | 0.55 | 0.49 | 2.43 | -0.06 |
| 713.00 | GBM | 0.90 | 1 | 0.11 | 0.58 | 0.84 | -0.51 | -0.14 | 0.70 | -3.02 | 1.53 |
| 683.00 | GBM | 12.10 | 1 | 0.47 | 0.48 | 1.46 | 2.09 | 0.30 | 0.13 | 0.53 | 0.24 |
| 439.00 | GBM | 18.83 | 0 | 0.62 | 0.07 | -0.29 | -0.21 | 0.25 | 0.01 | -0.33 | -0.35 |
| 573.00 | GBM | 18.47 | 0 | 0.89 | 0.30 | 0.32 | -0.54 | -0.57 | 0.80 | -4.16 | 0.50 |
| 346.00 | GBM | 3.47 | 1 | 0.71 | 0.21 | 0.97 | 0.95 | 1.58 | 0.54 | 0.96 | 0.57 |
| 664.00 | GBM | 26.33 | 0 | 0.70 | 0.00 | 0.02 | 1.20 | -2.45 | 0.23 | -4.36 | 0.04 |
| 203.00 | GBM | 6.27 | 1 | 0.56 | 0.66 | 0.27 | 0.47 | -0.57 | 0.67 | -2.84 | 1.18 |
| 404.00 | GBM | 0.00 | 0 | 0.82 | 0.58 | 0.75 | 0.66 | -0.93 | 0.44 | 0.66 | -0.02 |
| 366.00 | GBM | 8.50 | 1 | 1.19 | 0.37 | -0.14 | 0.00 | 2.09 | 2.12 | -0.96 | 1.02 |
| 654.00 | GBM | 21.33 | 1 | 0.87 | 0.04 | 2.30 | -0.95 | -0.04 | 0.69 | -5.54 | 2.09 |
| 292.00 | GBM | 15.93 | 1 | 0.89 | 0.49 | -0.29 | 0.84 | -0.57 | 0.25 | 0.86 | 0.41 |
| 593.00 | GBM | 8.10 | 1 | 1.15 | 0.64 | 0.62 | -0.43 | -0.43 | 2.55 | -4.15 | 0.26 |
| 604.00 | GBM | 12.70 | 1 | 0.17 | 0.19 | 1.90 | -1.89 | 0.49 | 0.85 | 1.01 | 1.50 |
| 597.00 | GBM | 17.50 | 0 | 1.64 | 0.05 | -0.14 | -1.22 | 0.28 | 0.60 | -2.98 | 1.11 |
| 373.00 | GBM | 9.37 | 1 | 1.45 | 0.33 | 0.64 | 4.00 | 0.04 | 1.58 | -5.01 | 1.74 |
| 454.00 | GBM | 13.73 | 1 | 0.57 | 0.68 | 0.99 | -1.73 | 0.05 | 0.40 | -3.18 | 1.42 |
| 371.00 | GBM | 22.37 | 0 | 0.18 | 0.28 | 0.42 | 1.07 | 0.35 | 1.81 | -4.38 | 1.06 |
| 210.00 | GBM | 10.00 | 1 | 0.53 | 0.17 | 0.64 | -2.29 | 0.35 | 0.25 | 1.37 | 0.29 |
| 640.00 | GBM | 27.43 | 0 | 1.10 | 0.94 | 1.91 | 0.25 | 0.80 | 1.52 | -3.51 | 0.86 |
| 606.00 | GBM | 10.83 | 1 | 1.01 | 0.24 | 1.09 | 2.22 | 0.38 | 1.26 | -1.87 | 0.65 |
| 775.00 | GBM | 8.43 | 1 | 0.58 | 0.82 | 1.80 | 1.46 | -0.74 | 0.42 | -0.07 | 0.79 |
| 588.00 | GBM | 17.77 | 0 | 0.95 | 0.04 | 1.19 | -0.19 | -1.54 | 2.12 | -0.67 | 2.22 |
| 684.00 | GBM | 23.80 | 1 | 1.02 | 0.02 | 1.02 | 3.23 | -0.53 | 0.71 | -2.52 | 0.82 |
| 345.00 | GBM | 23.80 | 0 | 0.76 | 0.63 | 0.46 | 3.77 | -0.14 | 1.42 | -3.94 | 1.33 |
| 442.00 | GBM | 18.63 | 0 | 0.60 | 0.14 | -0.24 | -1.53 | -0.46 | -0.29 | -1.06 | 0.94 |
| 335.00 | GBM | 12.83 | 1 | 1.21 | 0.68 | -0.57 | -1.69 | -1.28 | 0.69 | -2.77 | -0.39 |
| 557.00 | GBM | 8.57 | 1 | 0.84 | 0.21 | 0.35 | 2.73 | 0.43 | 2.35 | -4.21 | 1.50 |
| 172.00 | GBM | 15.40 | 1 | 1.13 | 0.00 | 2.30 | 4.49 | 0.88 | 1.18 | 0.51 | 1.10 |
| 764.00 | GBM | 2.23 | 1 | 0.76 | 0.51 | 1.12 | -0.48 | 0.20 | 0.50 | -2.97 | 0.81 |
| 436.00 | GBM | 18.93 | 0 | 0.96 | 0.31 | 1.12 | 3.01 | 0.00 | 0.83 | -4.19 | 0.80 |
| 104.00 | GBM | 5.40 | 1 | 0.07 | 0.18 | -0.62 | -3.39 | -1.57 | -0.85 | -1.58 | -1.19 |
| 225.00 | GBM | 34.83 | 0 | 1.91 | 0.90 | -0.87 | -4.71 | 0.17 | -0.11 | -4.78 | 0.23 |
| 377.00 | GBM | 13.30 | 1 | 1.97 | 0.92 | -0.80 | 1.08 | -1.25 | 0.82 | 1.29 | 0.15 |
| 342.00 | GBM | 17.53 | 1 | 1.04 | 0.26 | -0.12 | 4.20 | 1.55 | 1.37 | -4.93 | 1.15 |
| 798.00 | GBM | 4.53 | 1 | 0.51 | 0.41 | 0.20 | 1.43 | -1.82 | 0.98 | 0.62 | -0.59 |
| 822.00 | GBM | 19.77 | 1 | -0.10 | 0.21 | -0.74 | 1.72 | -1.74 | 0.39 | -3.58 | -1.14 |
| 264.00 | GBM | 12.77 | 1 | 1.06 | -0.09 | 0.20 | -2.27 | -0.19 | 0.33 | 1.44 | 0.97 |
| 380.00 | GBM | 5.50 | 1 | 0.42 | 0.71 | 0.36 | 1.13 | -0.97 | 0.80 | -3.92 | -0.06 |
| 714.00 | GBM | 13.80 | 1 | 1.53 | 0.70 | 0.52 | -0.77 | -0.64 | 1.59 | -1.66 | -0.10 |
| 156.00 | GBM | 5.97 | 1 | -0.70 | 0.33 | -0.74 | 3.60 | -1.95 | -0.44 | -2.88 | -0.20 |
| 11.00 | GBM | 5.17 | 1 | -1.33 | -0.26 | -0.67 | -2.34 | -1.51 | -1.80 | -2.04 | -0.61 |
| 518.00 | GBM | 6.80 | 1 | 0.52 | -0.04 | 0.28 | -0.05 | -0.38 | 0.00 | 1.37 | 0.30 |
| 240.00 | GBM | 12.87 | 1 | 1.10 | 0.15 | -0.17 | 5.42 | -0.48 | 1.75 | -4.20 | 0.84 |
| 401.00 | GBM | 5.60 | 1 | 1.74 | 0.07 | -1.02 | -0.70 | -0.97 | 1.14 | -1.54 | 0.87 |
| 218.00 | GBM | 10.43 | 1 | 0.46 | 0.69 | -0.77 | 1.11 | -1.81 | 0.88 | -3.04 | -0.11 |
| 527.00 | GBM | 14.63 | 1 | 1.20 | 0.42 | 0.05 | -1.01 | -0.98 | 0.07 | -0.38 | 1.11 |
| 635.00 | OA | 25.30 | 0 | -0.88 | -1.24 | -0.21 | -0.38 | 2.32 | 0.09 | 1.20 | -0.01 |
| 523.00 | OA | 29.60 | 0 | -1.84 | 0.09 | -1.01 | -1.29 | 0.88 | -0.53 | 1.66 | -0.74 |
| 498.00 | OA | 11.87 | 1 | -0.66 | -0.03 | -0.42 | -1.17 | 0.57 | -1.08 | 2.25 | -1.00 |
| 513.00 | OA | 35.40 | 0 | -0.23 | -0.22 | -0.32 | 1.30 | 0.57 | -0.11 | 1.21 | -0.43 |
| 674.00 | OA | 23.67 | 0 | -1.13 | -0.72 | -0.70 | -1.04 | 0.78 | -0.96 | 1.35 | -0.68 |
| 266.00 | OA | 42.17 | 0 | 0.55 | -0.47 | 1.32 | -1.71 | 2.35 | 1.58 | -4.17 | 0.48 |
| 291.00 | OA | 40.77 | 0 | -0.81 | -0.35 | 0.27 | 0.47 | 1.39 | -0.16 | 0.57 | 0.14 |
| 418.00 | OA | 34.50 | 0 | -1.41 | -1.07 | 0.03 | -0.17 | 1.91 | -0.31 | 0.65 | -0.09 |
| 352.00 | OA | 43.03 | 0 | -1.62 | -0.66 | -1.53 | -0.05 | 0.67 | -1.38 | 0.46 | -1.18 |
| 191.00 | OA | 45.90 | 0 | -0.40 | 0.20 | -1.13 | -2.04 | 1.21 | -1.08 | 1.87 | -0.91 |
| 315.00 | OA | 39.40 | 0 | -0.05 | -0.47 | 0.30 | 0.79 | 1.45 | 0.06 | 1.70 | 0.37 |
| 596.00 | OA | 32.07 | 0 | 0.64 | 0.23 | 0.68 | -1.17 | 0.28 | -0.13 | -1.19 | 0.35 |
| 232.00 | OA | 14.30 | 1 | 0.52 | 0.22 | 1.02 | -2.08 | -1.52 | 0.05 | -2.88 | -0.30 |
| 296.00 | OA | 40.50 | 0 | -0.57 | -0.79 | 1.40 | 0.48 | -0.08 | 0.60 | -1.08 | 0.26 |
| 378.00 | OA | 36.50 | 0 | -0.20 | -0.51 | -0.23 | 1.69 | 1.34 | 0.13 | -0.27 | 0.52 |
| 348.00 | OA | 38.13 | 0 | -0.12 | -0.16 | 0.70 | 0.39 | 1.24 | 0.75 | 1.52 | 0.47 |
| 231.00 | OA | 4.83 | 1 | 0.62 | 0.30 | 0.21 | 4.27 | -1.16 | -0.13 | -1.69 | 0.52 |
| 351.00 | OA | 24.73 | 1 | 0.81 | 0.06 | 0.60 | -2.75 | -0.49 | -0.69 | 2.12 | 0.01 |
| 671.00 | OA | 23.70 | 0 | -1.01 | -0.32 | -0.50 | 0.64 | 2.06 | -0.02 | 0.46 | -0.51 |
| 411.00 | OA | 35.03 | 0 | -0.96 | -0.70 | -0.32 | 1.08 | 1.06 | 0.02 | 2.56 | 0.22 |
| 528.00 | OA | 29.53 | 0 | 0.16 | -0.24 | -0.64 | 0.57 | 0.47 | -0.34 | -1.60 | -0.50 |
| 273.00 | OA | 41.73 | 0 | -0.74 | -0.23 | 0.35 | 0.90 | -0.94 | 0.63 | -1.56 | 0.78 |
| 312.00 | OA | 28.00 | 1 | 0.01 | 0.02 | -0.02 | 0.68 | -1.04 | 0.37 | -3.00 | -0.79 |
| 259.00 | OA | 18.30 | 1 | 0.55 | 0.03 | -0.18 | -0.68 | -0.14 | -1.15 | -0.43 | -0.70 |
| 623.00 | OA | 26.80 | 0 | -1.54 | -0.09 | -0.03 | 0.67 | 1.94 | 0.36 | 1.56 | 0.21 |
| 519.00 | OA | 29.83 | 0 | -0.84 | 0.08 | -0.27 | 1.18 | 1.36 | -0.08 | 2.04 | -0.65 |
| 492.00 | OA | 21.73 | 0 | -0.42 | 0.67 | 2.02 | -3.69 | 0.41 | -0.28 | 0.40 | 0.38 |
| 514.00 | OA | 30.03 | 0 | -0.29 | -0.33 | -0.74 | 1.05 | 1.64 | 0.89 | -4.35 | 0.15 |
| 662.00 | OA | 10.00 | 1 | -0.76 | -0.10 | -1.03 | 1.31 | 1.99 | -0.29 | -0.86 | -0.66 |
| 393.00 | OA | 15.43 | 0 | -0.57 | -0.39 | -0.39 | -2.86 | 0.17 | -1.08 | -4.80 | -0.69 |
| 438.00 | OA | 25.90 | 1 | 0.06 | 0.44 | 0.03 | -2.33 | -1.35 | 0.32 | -0.44 | -0.54 |
| 615.00 | OA | 26.07 | 0 | -0.20 | 0.41 | -0.39 | 2.19 | 0.30 | 0.15 | -0.68 | -0.83 |
| 391.00 | OA | 41.13 | 0 | -1.17 | -0.29 | -0.43 | -0.33 | 0.38 | -0.78 | -4.39 | -1.14 |
| 562.00 | OA | 9.67 | 1 | -0.12 | 0.27 | 0.32 | -2.67 | -1.22 | -0.49 | -0.62 | 0.43 |
| 329.00 | OA | 13.97 | 1 | 0.46 | 0.86 | 0.04 | -2.11 | -1.62 | 0.82 | -4.55 | -0.73 |
| 282.00 | OA | 8.00 | 1 | 0.56 | 0.56 | 0.98 | -0.03 | -1.37 | 1.03 | -5.28 | 1.66 |
| 406.00 | OA | 9.67 | 1 | 0.12 | 1.09 | -0.05 | -3.53 | -2.25 | -0.44 | -2.74 | -0.87 |
| 275.00 | OD | 40.70 | 0 | -1.43 | -0.88 | -0.84 | -1.39 | 1.41 | 0.20 | 1.15 | 0.26 |
| 577.00 | OD | 17.53 | 0 | -1.03 | 0.44 | -0.88 | -1.84 | 0.76 | -0.84 | 2.04 | -0.73 |
| 323.00 | OD | 39.07 | 0 | -1.29 | -0.46 | -0.47 | -1.13 | 1.54 | -1.18 | 1.93 | -0.63 |
| 485.00 | OD | 31.23 | 0 | -1.23 | -1.10 | -0.56 | -0.98 | 1.76 | -0.39 | 0.32 | -0.34 |
| 387.00 | OD | 36.13 | 0 | -0.73 | 0.01 | 0.09 | -0.19 | -0.13 | -0.29 | 2.19 | -0.66 |
| 484.00 | OD | 31.37 | 0 | -1.45 | -0.20 | -0.48 | 0.29 | 1.44 | -0.07 | 1.12 | 0.40 |
| 579.00 | OD | 27.70 | 0 | -0.57 | -0.54 | -0.24 | 0.17 | 1.70 | -0.06 | -2.94 | 0.07 |
| 659.00 | OD | 24.17 | 0 | -0.54 | -0.46 | -0.12 | -1.18 | 1.30 | 0.07 | 4.23 | -0.16 |
| 314.00 | OD | 44.70 | 0 | -0.75 | 0.14 | 0.64 | -0.70 | 1.45 | -0.72 | 3.59 | -0.50 |
| 482.00 | OD | 31.40 | 0 | -1.14 | -0.70 | 0.71 | -0.17 | 0.78 | 0.25 | 2.98 | 2.76 |
| 639.00 | OD | 25.07 | 0 | -0.50 | -0.32 | -0.33 | 1.11 | 1.07 | 0.22 | 0.47 | 1.07 |
| 398.00 | OD | 35.43 | 0 | -0.82 | -0.07 | -0.05 | 0.49 | 0.82 | -0.09 | 1.43 | -0.15 |
| 543.00 | OD | 28.90 | 0 | -0.88 | -0.25 | -0.66 | 1.20 | 1.39 | 0.13 | 0.27 | 0.25 |
| 558.00 | OD | 20.43 | 1 | -0.39 | 0.37 | -0.23 | 1.06 | 1.65 | -0.43 | 0.75 | -0.53 |
| 260.00 | OD | 18.90 | 1 | 0.84 | 0.33 | -0.25 | -2.79 | -0.62 | -1.28 | 0.82 | -0.74 |
| 455.00 | OD | 32.80 | 0 | -0.54 | 0.26 | -0.68 | 0.94 | 1.56 | 0.06 | 0.23 | -0.57 |
| 490.00 | OD | 36.57 | 0 | 0.44 | 0.12 | -0.48 | 1.50 | 0.89 | -0.04 | 0.58 | -0.13 |
| 672.00 | OD | 23.70 | 0 | 0.01 | -0.49 | -0.54 | 1.34 | 1.12 | 0.49 | 2.17 | -0.15 |
| 633.00 | OD | 25.37 | 0 | 0.13 | 0.06 | -0.81 | 0.49 | 0.16 | -0.45 | -1.62 | -0.12 |
| 489.00 | OD | 21.77 | 0 | -1.10 | 0.23 | 0.87 | -0.49 | -0.03 | -1.36 | -2.55 | -0.44 |
| 589.00 | OD | 27.20 | 0 | -0.45 | -0.51 | 0.73 | 0.80 | 0.99 | 0.27 | 0.47 | 0.62 |
| 500.00 | OD | 30.87 | 0 | -0.30 | -0.41 | -0.65 | 0.47 | 1.20 | -0.60 | -1.76 | -0.26 |
| 403.00 | OD | 38.83 | 0 | -1.10 | 0.45 | 0.49 | 1.87 | -0.03 | 0.00 | -3.44 | -1.45 |
| 446.00 | OD | 33.23 | 0 | -1.13 | -0.13 | -0.66 | 0.78 | 0.33 | -0.14 | -0.98 | 0.17 |
| 598.00 | OD | 32.03 | 0 | -1.08 | 0.55 | -0.89 | 1.04 | 0.57 | 0.59 | -0.76 | -0.52 |
| 474.00 | OD | 36.80 | 0 | 0.23 | 0.00 | -0.06 | 0.16 | -0.80 | -0.36 | -3.00 | -0.81 |
| 364.00 | OD | 29.27 | 1 | 1.32 | 0.56 | -0.42 | -0.71 | -1.57 | 0.38 | -0.70 | 0.74 |
| 508.00 | OD | 9.50 | 1 | 0.65 | -0.68 | -0.66 | 3.88 | -2.13 | 0.89 | -2.60 | -0.31 |
| E166 | NG |  |  | -1.68 | -0.70 | -0.58 | -1.48 | 1.62 | -1.15 | 0.69 | -0.59 |
| E207 | NG |  |  | -0.81 | -0.68 | -0.06 | -0.64 | 1.06 | -0.08 | 2.34 | -0.37 |
| NB05 | NG |  |  | -1.92 | -0.23 | -1.11 | -2.26 | 0.36 | -1.05 | 1.11 | -1.20 |
| NB06 | NG |  |  | -1.71 | -0.92 | -0.69 | -2.26 | 0.74 | -1.81 | 1.16 | -1.15 |
| NB07 | NG |  |  | -2.04 | -0.09 | -1.07 | -1.96 | 0.81 | -0.97 | 0.24 | -0.66 |

Abbreviations: GBM, glioblastoma; A, astrocytoma; OA, oligoastrocytoma; OD, oligodendroglioma; NG, nonglioma.
