# Supplementary figures and images for: Integrated analysis of 34 microarray datasets reveals CBX3 as a diagnostic and prognostic biomarker in glioblastoma
Source: J Transl Med. 2019 May 28;17:179. doi: 10.1186/s12967-019-1930-3 (PMC6540543; doi:10.1186/s12967-019-1930-3)

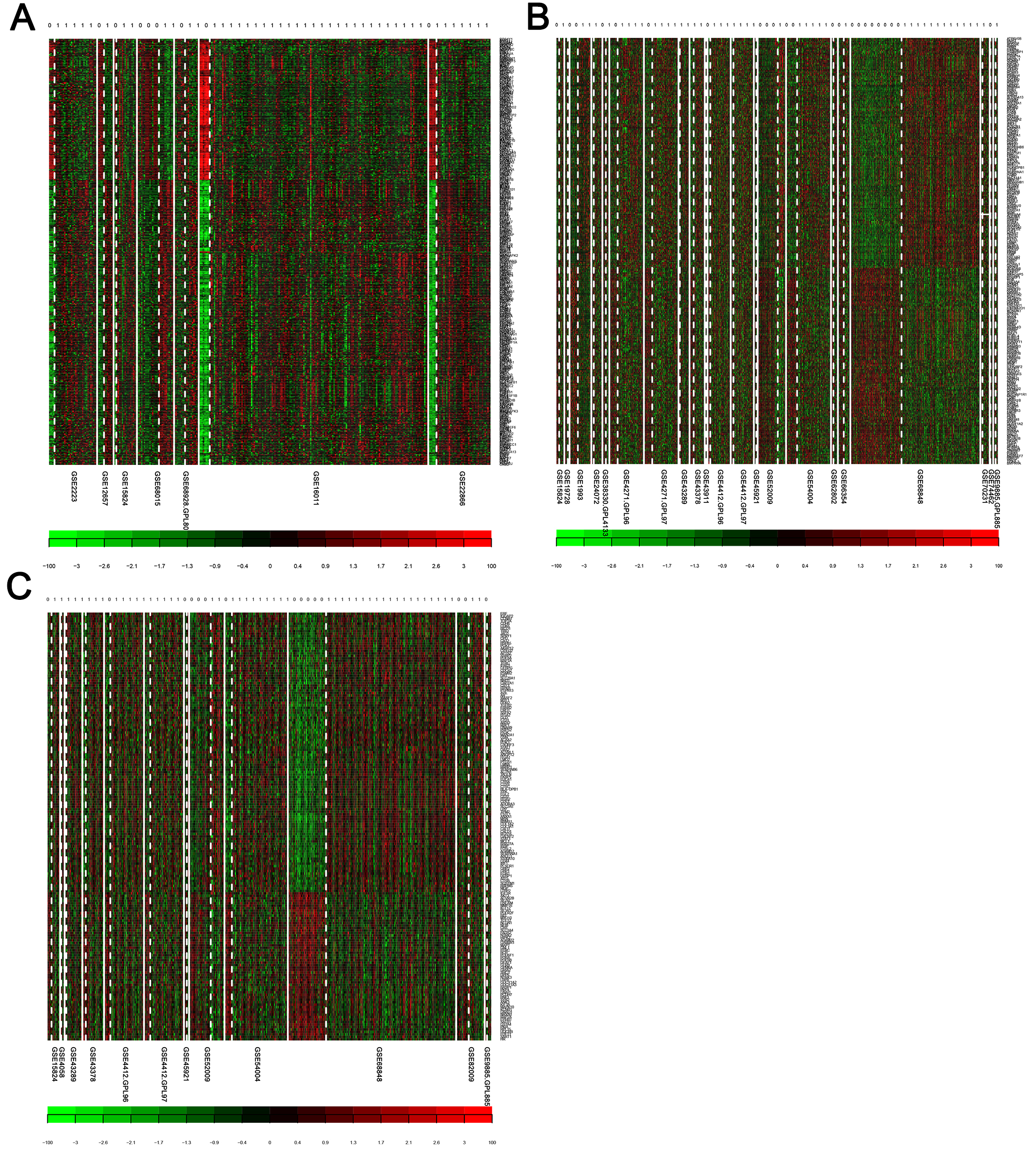

Supplement: Supplementary file 2 — Additional file 2: Figure S1. Clustering analyses. (A) Clustering analyses performed with 7 datasets in the validation cohorts of GBM vs NG. (B) Clustering analyses performed with 21 datasets in the validation cohorts of GBM vs A. (C) Clustering analyses performed with 12 datasets in the validation cohorts of GBM vs OD. Abbreviations: GBM, glioblastoma; NG, nonglioma; A, astrocytoma; OD, oligodendroglioma. [file 12967_2019_1930_MOESM2_ESM.jpg]

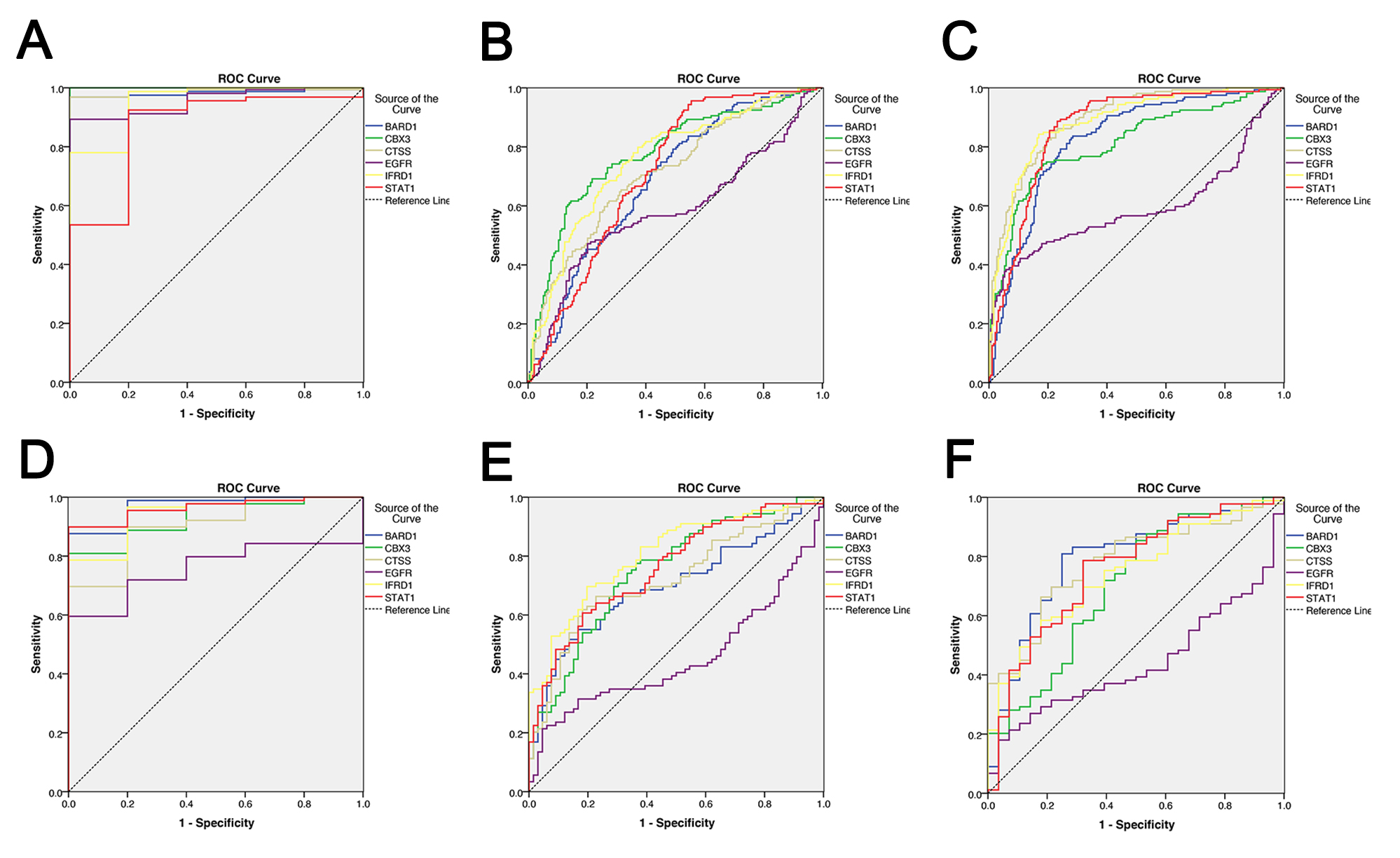

Supplement: Supplementary file 3 — Additional file 3: Figure S2. ROC analysis in the TCGA-GBMLGG and CGGA datasets. Expression levels of the six upregulated DEGs in GBM vs. NG (A), GBM vs. A (B), and GBM vs. OD (C) tissues in the TCGA-GBMLGG cohort. Expression levels of the six upregulated DEGs in GBM vs. NG (D), GBM vs. A (E), and GBM vs. OD (F) tissues in the CGGA cohort. Abbreviations: TCGA, The Cancer Genome Atlas; CGGA, the Chinese Glioma Genome Atlas; GBM, glioblastoma; NG, nonglioma; A, astrocytoma; OD, oligodendroglioma. [file 12967_2019_1930_MOESM3_ESM.jpg]

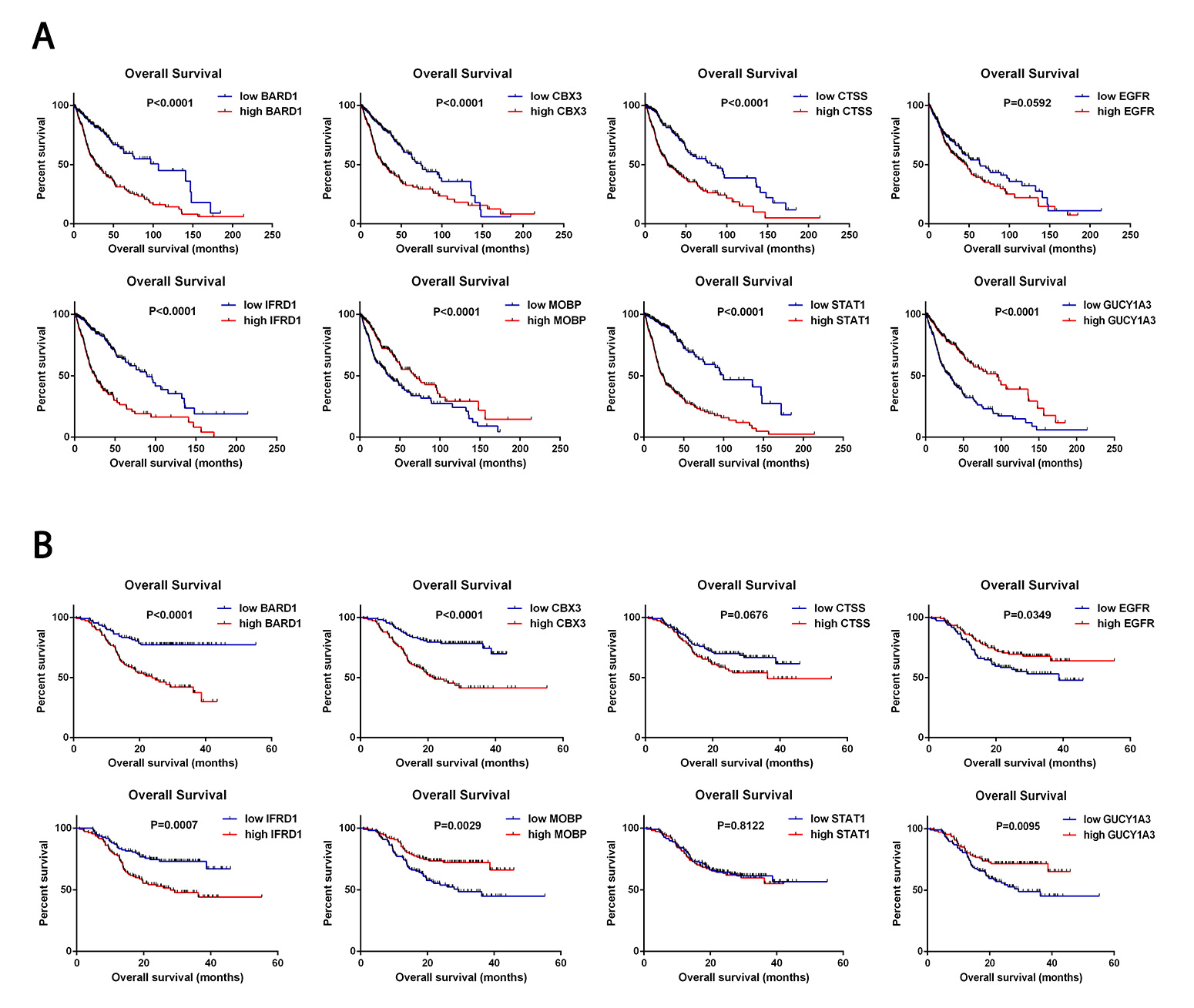

Supplement: Supplementary file 4 — Additional file 4: Figure S3. Survival analysis results in TCGA-GBMLGG and CGGA datasets excluding G-CIMP positive patients. Kaplan-Meier analyses were performed based on the median expression levels of the eight DEGs in the TCGA-GBMLGG (A) and CGGA (B) cohorts. The tick marks on the Kaplan-Meier survival curves represent the censored subjects. Abbreviations: TCGA, The Cancer Genome Atlas; CGGA, the Chinese Glioma Genome Atlas. [file 12967_2019_1930_MOESM4_ESM.jpg]
